# Duplicated Leptin Receptors in Two Species of Eel Bring New Insights into the Evolution of the Leptin System in Vertebrates

**DOI:** 10.1371/journal.pone.0126008

**Published:** 2015-05-06

**Authors:** Marina Morini, Jérémy Pasquier, Ron Dirks, Guido van den Thillart, Jonna Tomkiewicz, Karine Rousseau, Sylvie Dufour, Anne-Gaëlle Lafont

**Affiliations:** 1 Muséum National d'Histoire Naturelle, Sorbonne Universités, Research Unit BOREA, Biology of Aquatic Organisms and Ecosystems, CNRS 7208, IRD207, UPMC, UCBN, Paris, France; 2 ZF-screens B.V., Leiden, The Netherlands; 3 Institute of Biology, Leiden University, Leiden, The Netherlands; 4 Technical University of Denmark, National Institute of Aquatic Resources, Charlottenlund, Denmark; Institute of Marine Research, NORWAY

## Abstract

Since its discovery in mammals as a key-hormone in reproduction and metabolism, leptin has been identified in an increasing number of tetrapods and teleosts. Tetrapods possess only one leptin gene, while most teleosts possess two leptin genes, as a result of the teleost third whole genome duplication event (3R). Leptin acts through a specific receptor (LEPR). In the European and Japanese eels, we identified two leptin genes, and for the first time in vertebrates, two LEPR genes. Synteny analyses indicated that eel LEPRa and LEPRb result from teleost 3R. LEPRb seems to have been lost in the teleost lineage shortly after the elopomorph divergence. Quantitative PCRs revealed a wide distribution of leptins and LEPRs in the European eel, including tissues involved in metabolism and reproduction. Noticeably, leptin1 was expressed in fat tissue, while leptin2 in the liver, reflecting subfunctionalization. Four-month fasting had no impact on the expression of leptins and LEPRs in control European eels. This might be related to the remarkable adaptation of silver eel metabolism to long-term fasting throughout the reproductive oceanic migration. In contrast, sexual maturation induced differential increases in the expression of leptins and LEPRs in the BPG-liver axis. Leptin2 was strikingly upregulated in the liver, the central organ of the reproductive metabolic challenge in teleosts. LEPRs were differentially regulated during sexual maturation, which may have contributed to the conservation of the duplicated LEPRs in this species. This suggests an ancient and positive role of the leptin system in the vertebrate reproductive function. This study brings new insights on the evolutionary history of the leptin system in vertebrates. Among extant vertebrates, the eel represents a unique case of duplicated leptins and leptin receptors as a result of 3R.

## Introduction

Leptin was first characterized in mouse, as the 16kDa amino acid product of the *obese* (*ob*) gene [1]. This protein belongs to the class I cytokine superfamily, and more particularly to the long-chain class-I helical cytokines, as growth hormone (GH) or prolactin (PRL) [2]. The absence of leptin expression, as a result of a mutation in the *ob* gene, induces severe obesity in mice, concomitant with multiple hormonal and metabolic alterations [1]. The characterization of the *obese* gene in human, and its location on chromosome 7, was published soon afterward [3]. Mutation in this gene also results in severe obesity in human [4].

The amino acid sequence of leptin is highly variable among vertebrates [5]. As a consequence, the first non-mammalian leptin was only characterized a decade after the discovery of the *obese* gene in mammals, by the means of gene synteny. This first non-mammalian leptin, characterized in a teleost, the fugu, *Takifugu rubripes*, shares only 13.2% of identity with the human leptin [6]. Since then, leptins have been identified and cloned in an increasing number of tetrapod and teleost species. In tetrapods, only one leptin gene has been characterized (salamander (*Ambystoma tigrinum*) [7], African clawed frog (*Xenopus laevi*s) [8], sauropsids: [9–11]), whereas two leptin genes have been identified in most of the teleost species investigated so far, such as zebrafish (*Danio rerio*) [12], medaka (*Oryzias latipes*) [13], orange spotted grouper (*Epinephelus coioides*) [14], *Schizothorax prenanti* [15], striped bass (*Morone saxatilis*) [16]. The presence of two leptin genes in teleosts was shown to be related to the third round of whole genome duplication (3R) that occurred specifically in this lineage [12, 13, 17]. Vertebrate leptins share limited primary sequence identity but a highly conserved gene structure, formed by two small exons separated by an intron [2]. The predicted secondary and tertiary structures of vertebrate leptins are also highly conserved. As all the class-I helical cytokines, leptin tertiary structure is characterized by four tightly packed alpha-helices [2]. Two cysteines, conserved among vertebrate species, form a disulphide bridge responsible for the stabilization of the tertiary structure of leptin [2, 5].

In mammals, leptin, secreted by adipocytes, is a circulating hormone that can cross the blood-brain barrier to reach the hypothalamus and regulate food intake and energy metabolism [5, 18]. This hormone is also involved in numerous other physiological processes, such as reproduction, growth, development, hematopoiesis, immune response, stress, and bone formation (for review [5, 19–25]). In teleosts, leptin has also been suggested to be involved in various functions, such as reproduction, modulation of feeding behavior, fat metabolism, and stress (for review [22]).

The specific receptor of leptin (LEPR) was first characterized in mice, shortly after the discovery of leptin [26]. In human, LEPR was shown to be localized on the chromosome 1 [27]. LEPR belongs to the class-I helical cytokine receptor superfamily and forms homodimers located at the cell membrane [2]. The intracellular signaling occurs through the JAK/STAT pathway [28–30]. In non mammalian vertebrates, LEPR sequences have been identified in some species, such as in the chicken (*Gallus gallus*) [31], African clawed frog [8], zebrafish [32], medaka [13], goldfish (*Carassius auratus*) [33], Atlantic salmon (*Salmo salar*) [34], orange spotted grouper [14], and fugu [35]. Contrary to the duplicated leptin genes, only one leptin receptor gene had been described so far in teleosts, as in tetrapods.

In this study, we investigated the leptin system, hormone and receptor, in two eel species (*Anguilla anguilla* and *Anguilla japonica*). These two eel species, which share a similar diadromous life cycle with long migrations, belong to two distinct clusters (Atlantic and Indo-Pacific) among the genus *Anguilla* [36]. Due to their phylogenetic position, as members of an early-emerging group among teleosts (elopomorphs) [37], eels may provide insights into ancestral regulatory functions in teleosts, the largest group of vertebrates [38, 39]. Furthermore, due to their striking life cycle, eels are a particularly interesting model to study the leptin system. Eels accumulate metabolic stores during the growth phase in continental waters, stop feeding at the pre-pubertal stage, and fast during the reproductive oceanic migration. Their metabolic stores will be mobilized to perform both the long oceanic journey and the sexual maturation [40].

In the present study, we characterized two leptin genes in the European and Japanese eels, as in most other teleost species, and report, for the first time in vertebrates, the presence of two leptin receptor genes. We investigated the origin of duplicated leptin and leptin receptor genes, by means of phylogenetic and synteny analyses, with a special focus on vertebrate species of key-phylogenetic positions. Finally, we compared the tissue distribution of these four genes in the European eel, *Anguilla anguilla*, and examined their regulation in the Brain-Pituitary-Gonad (BPG)-liver axis, during experimental maturation.

## Materials and Methods

### *In silico* prediction of leptin and LEPR genes

#### Eel genome

The TBLASTN algorithm of the CLC DNA Workbench 6 software (CLC bio, Aarhus, Denmark) was used to identify the leptin and LEPR genomic sequences in the European and Japanese eel, *Anguilla japonica*, genome databases [38, 39]. The two leptin peptide sequences of zebrafish (CAP47064.1, CAP15930.1), on one hand, and the zebrafish and Atlantic salmon LEPR peptidic sequences (NP_001106847, NP_001158237), on the other hand, were used as queries. The empirical nucleotide splicing signature was used to predict the exons and splicing junctions from the genomic databases, i.e. introns begin with “GT” and end with “AG”.

#### Spotted gar genome

The TBLASTN algorithm of the Ensembl Genome Browser (v75) website (http://www.ensembl.org/index.html) was used to retrieve the LEPR sequence from the spotted gar, *Lepisosteus oculatus*, genome database (LepOcu1). The partial spotted gar LEPR sequence (ENSLOCG00000006903), the zebrafish and Atlantic salmon LEPR peptidic sequences (NP_001106847, NP_001158237), in addition to the two eel LEPR sequences characterized in the present study (named eel LEPRa and LEPRb), were used as queries.

### Synteny analyses

#### Leptin genomic regions

Neighboring genomic regions of the duplicated eel leptins were characterized manually on the Japanese eel genomic database, using CLC DNA Workbench 6 software. The genes located in the same scaffolds as leptin were identified. Their potential paralogs were searched in the European and Japanese eel genomes. Their homologs were then identified in the other vertebrate genomes, using Genomicus PhyloView of Genomicus v77.01. Leptin genomic regions were compared in a representative sarcopterygian (human, *Homo sapiens*) and in representative actinopterygians, including spotted gar and teleosts (zebrafish, medaka, stickleback, *Gasterosteus aculeatus*, and fugu). For each leptin neighboring gene family, when only one gene was annotated in all the above-mentioned genomes, BLAST analyses were performed to search for potential additional paralogs.

#### LEPR genomic regions

Neighboring genomic regions of the duplicated eel LEPRs were characterized manually on the European and Japanese eel genomic databases, using CLC DNA Workbench 6 software. The genes located in the same scaffolds as LEPR were identified. Further analysis of the two eel draft genomes allowed us to assemble two or three scaffolds and increase the number of identified neighboring genes. Potential paralogs of the neighboring genes were searched in the eel genomes. Homologs of the eel LEPR neighboring genes were identified in the other vertebrate genomes, using Genomicus PhyloView of Genomicus v77.01. LEPR genomic regions were compared in representative sarcopterygian species including coelacanth and tetrapods (human, opossum, *Monodelphis domestica*, lizard, *Anolis carolinensis*, Chinese softshell turtle, *Pelodiscus sinensis*, chicken, Western clawed frog, *Xenopus tropicalis)*, and actinopterygian species including spotted gar and teleosts (medaka, tetraodon, *Tetraodon nigroviridis*, and stickleback). BLAST analyses were also performed to search for potential additional LEPR genes in all the above-mentioned genomes. For each teleost species, the spotted gar LEPR, the two eel LEPR identified in this study, and LEPR from various teleosts were used as queries. Similarly, BLAST searches were performed in all the above-mentioned genomes to identify non-annotated LEPR neighboring genes.

### Phylogenetic analyses

#### Sequence alignments

Multiple sequence alignments of the protein families were created using Clustal Omega [41] included in SeaView version 4.4.2. The alignments were manually edited to adjust poorly aligned sequence stretches. Amino acids highly relevant for secondary and tertiary structures of the proteins, such as cysteines, were particularly investigated. Manual adjustment also took into account conserved chemical properties of amino acids. In the case of the receptor, special focus was given to the alignment of conserved domains. Calculation of best amino acid substitution matrix was determined using the Protest software [42]. Phylogenetic analyses of the resulting protein alignments were performed using the Maximum Likelihood method with 1,000 bootstrap replicates (RaxML software [43], www.phylo.org).

#### Leptin

Amino acid sequences of 34 actinopterygian leptins (33 sequences from teleost species and one sequence from a non-teleost actinopterygian species, the spotted gar) were retrieved from NCBI and Ensembl databases. Human and bull, *Bos taurus*, leptins were used as outgroup (for references of sequences see S1 Table).

#### Leptin receptor (LEPR)

Amino acid sequences of 21 vertebrate LEPR were retrieved from NCBI and Ensembl databases. The vertebrate species included sarcopterygians (tetrapods and a basal sarcopterygian, the coelacanth, *Latimeria chalumnae*), and actinopterygians (teleosts and a non-teleost actinopterygian, the spotted gar). Human and zebrafish G-CSFR (Granulocyte colony-stimulating factor receptor) sequences, which belong to the class-I helical cytokine receptor superfamily [2], were used as outgroup (for references of sequences see S2 Table).

#### Leptin neighboring genes and LEPR neighboring genes

Amino acid sequences of the selected neighboring gene families were searched in NCBI and Ensembl databases (for references of sequences see S3 Table). These sequences were retrieved from vertebrate species including various sarcopterygians and actinopterygians. BLAST searches were performed to identify non-annotated genes in the genomes of the species included in this study. For LEPR neighboring genes, phylogenetic analyses were performed on gene families, for which duplicated genes are present in eels and in the other teleost species investigated in this study, *i*.*e*. PDE4B, ANGPTL3, KANK4, LRP8, and TMEM125. Phylogenetic analyses were also performed on two examples of gene families, *i*.*e*. DNAJC6 and JAK1, for which, like for LEPR, duplicated genes have been identified only in the eels. For leptin, phylogenetic analyses were performed on neighboring gene families for which duplicated genes are present in eels and some of the other teleost species investigated in this study, *i*.*e*. PRRT4, LRRC4, and si:dkey-5i3.5. When only partial sequences could be retrieved for one of the investigated species, the sequences of the other species were shortened to the same size before processing with sequence alignment, in order to avoid artificial bias.

### European eel samples

#### Ethics statement

Experimental maturations were conducted on farmed female European eels, at a DTU Aqua research facility at Lyksvad Fishfarm, Vamdrup, Denmark. All fish were handled in accordance with the European Union regulations concerning the protection of experimental animals (Dir 86/609/EEC). Eel experimental protocol was approved by the Animal Experiments Inspectorate (AEI), Danish Ministry of Food, Agriculture and Fisheries (permit number: 2010/561-1783). This study began in 2010.

Female European eels used in this study were at the pre-pubertal stage prior to experiments. As eels undergo a natural fasting period from the pre-pubertal silver stage to the end of the sexual maturation, they were not fed during treatment. All eels were anesthetized using ethyl p-aminobenzoate (benzocaine; Sigma-Aldrich, Germany), before tagging, handling and sacrifice. All efforts were made to minimize animal handling and stress.

#### Experimental maturation

Farmed female eels, raised from glass eels to large size ~65–85 cm at a commercial eel farm, using freshwater aquaculture recirculation systems (RAS) at ~25°C, were selected and transferred to the research facility. Selection criteria included size and weight comparable to natural silvering females. At the facility, eels were acclimated to saltwater (36‰) and temperature (20°C) in 300 L RAS systems. The experimental protocol was established according to [44]. Thus, female eels received weekly injections of salmon pituitary extract (SPE Argent Chemical Laboratories, Washington, USA) at 18.75 mg/kg body weight for four months to induce vitellogenesis, followed by one dihydroxyprogesterone (17α,20ß-dihydroxy-4-pregnen-3-one; Sigma-Aldrich Denmark A/S) injection at 2 mg per/kg body weight to induce final oocyte maturation and ovulation.

Two series of experimental maturation (Experiments 1 and 2) were performed on independent batches of eels. Experiment 1: 10 control eels sacrificed at the beginning of the experiment (T0 Control) and 10 matured eels sacrificed at the end of the experiment. Experiment 2: 6 control eels sacrificed at the beginning of the experiment (T0 Control), 6 control eels sacrificed at the end of the experiment (End control) and 6 matured eels sacrificed at the end of the experiment. For both experiments, anterior brain (including olfactory bulbs, telencephalon, and di-/mesencephalon), pituitary, liver and ovary were sampled in RNAlater (Ambion) and stored at -20°C until RNA extraction.

#### Tissue distribution

Leptin and LEPR tissue distributions were analyzed on eight farmed female silver eels. The following tissues were sampled in RNAlater (Ambion Inc., Austin, TX, USA) and stored at -20°C until RNA extraction: olfactory bulbs, telencephalon, di-/mesencephalon, *cerebellum* and *medulla oblongata*, pituitary, eye, gills, liver, heart, spleen, peri-visceral adipose tissue, intestine, muscle and ovary.

#### RNA extraction

Extraction of total RNA was performed using mechanical homogenization (TissueLyser II, Qiagen, Hilden, Germany). Brain parts, pituitary, gills, heart, spleen and intestine were extracted using the Qiagen RNeasy Mini Kit (Qiagen). A deoxyribonuclease I treatment, using the Qiagen RNase-free DNase Set, was applied during the procedure, according to the manufacturer instructions. Eye, liver, adipose tissue, muscle and ovary were extracted using Trizol reagent (Invitrogen SARL, Cergy Pontoise, France) according to the manufacturer’s instructions. After extraction, a final deoxyribonuclease I (Roche Ltd., Basel, Switzerland) treatment was applied to each sample.

### European eel leptins and LEPRs full length cDNA cloning

Pituitary, ovary and liver total RNA, from control and matured European eels, were used for cDNA cloning of European eel leptins and LEPRs. One microgram of RNA was reverse transcribed using SuperScript III First Strand cDNA Synthesis Kit (Invitrogen) for classical PCR, or using SMART RACE cDNA amplification kit (Clontech Laboratories Inc., Palo Alto, CA., USA) for 3’ and 5’ RACE PCR.

Primers were designed using the Primer3 Software (Whitehead Institute/Massachusetts Institute of Technology, Boston, MA), and purchased from Eurofins (Elsersberg, Germany) (S4 Table).

The classical PCR were performed as follows: an initial step of polymerase activation for 3 min at 94°C; then 2 cycles with 30 s at 94°C, 30 s at 66°C, 2 min at 72°C; 2 cycles with 30 s at 94°C, 30 s at 64°C, 2 min at 72°C; 2 cycles with 30 s at 94°C, 30 s at 62°C, 2 min at 72°C; 2 cycles with 30 s at 94°C, 30 s at 60°C, 2 min at 72°C; 3 cycles with 30 s at 94°C, 30 s at 58°C, 2 min at 72°C; 3 cycles with 30 s at 94°C, 30 s at 57°C, 2 min at 72°C; 2 cycles with 30 s at 94°C, 30 s at 56°C, 2 min at 72°C; 3 cycles with 30 s at 94°C, 30 s at 55°C, 2 min at 72°C; 20 cycles with 30 s at 94°C, 30 s at 54°C, 2 min at 72°C, and a single final extension step of 5 min at 72°C.

The RACE PCR with 5’cDNA or 3’cDNA as templates were performed as follows: an initial step of polymerase activation for 3 min at 94°C; then 10 cycles with 30 s at 94°C, 30 s at 70°C, 3 min at 72°C; then 25 cycles with 30 s at 94°C, 30 s at 68°C, 3 min at 72°C; and a single final extension step of 5 min at 72°C.

PCR products of appropriate estimated size were isolated with the QUIAquick gel extraction Kit (Qiagen, Hilden, Germany), directly sequenced at GATC biotech Ltd. (Konstanz, Germany), or cloned using the TOPO TA Cloning Kit (Invitrogen) before sequencing.

The amino acid sequences of European eel leptins and LEPRs were predicted from the obtained complete cDNA sequences. The characteristic domains of LEPR were predicted using Interproscan software (http://www.ebi.ac.uk/interpro/).

### Quantitative real time PCR (qPCR)

#### Design of qPCR primers

Specific qPCR primers for the two European eel leptins (named leptin1 and leptin2) and the two European eel LEPR (named LEPRa and LEPRb) were designed using the Primer3 Software. For each gene, the forward and reverse primers were designed on two different exons in order to avoid a potential genomic DNA contamination of the tissue samples. All primers were purchased from Eurofins. The sequences are indicated in S4 Table.

#### Protocol

For qPCR analyses, 500 ng of each tissue total RNA was reverse transcribed using SuperScriptIII First Strand cDNA Synthesis Kit (Invitrogen). The qPCR analyses were performed with a Lightcycler (Roche, Ltd. Basel, Switzerland), using SYBR Green I sequence-unspecific detection. Each reaction contained 4 μL of diluted cDNA template, 2 μL of SYBR Green master mix, and 1 μL of each forward and reverse specific primers (0.5 pmole each at final concentration). The following program was applied for each gene: a polymerase activation step of 10 min at 95°C, followed by 41 to 51 cycles of 10 seconds of denaturizing at 95°C, 5 seconds of annealing at 60°C, 6 seconds of elongation at 72°C. The program ended with a melting curve analysis by slowly increasing the temperature (0.1°C/s) from 68°C to 95°C, with a continuous registration of changes in fluorescent emission intensity. This step aimed at ensuring the presence of only one amplified product.

#### Assessment of qPCR primer specificity

The specificity of the primers was verified by sequencing the amplification products (GATC Biotech Ltd.). To further assess the specificity of the qPCR primers, each couple was tested for its inability to amplify the transcript of the corresponding duplicated gene. PCR were conducted to obtain amplicons of leptin1, leptin2, LEPRa and LEPRb. Serial dilutions were performed on each amplicon. The qPCR primers were tested on a 51 cycle program. Leptin1 specific qPCR primers were not able to amplify the leptin2 amplicon, leptin2 qPCR primers were not able to amplify the leptin1 amplicon, LEPRa specific qPCR primers were not able to amplify the LEPRb amplicon, LEPRb specific qPCR primers were not able to amplify the LEPRa amplicon.

#### Assays of samples

Individual tissue samples were analyzed in duplicate. Each qPCR run contained a non-template control for each primer couple, by substituting the cDNA template with water. Serial dilutions of each specific amplicon were used as a standard curve for each gene. One dilution was included in each run as a calibrator. Normalization of the qPCR data was performed using total RNA content for the tissue distribution samples, and eel beta actin [45] as a reference gene for experimental maturation samples.

### Statistical analysis

Results are given as mean ± SD. Non-parametric tests were performed. Means were compared by Mann-Whitney *U* test or Kruskal-Wallis ANOVA using Instat (GraphPad Software Inc., San Diego, Calif., USA).

## Results and Discussion

### Characterization of eel leptins

#### European and Japanese eel leptin gene prediction

Two leptin genes were identified in each of the European and Japanese eel genomes. These genes display the classical conserved gene structure of two exons described in vertebrates (S1 Fig) [2].

For one gene, named here leptin1 gene, the complete CDS sequence was retrieved from both eel draft genomes. In both European and Japanese eels, leptin1 CDS is a 513 bp sequence, composed by a 150 bp exon1 and a 363 bp exon2. The resulting predicted amino acid sequence consists of 171 aa and contains a 21 amino acid signal peptide (SignalP 4.1 server). The predicted European eel leptin1 differs only by four amino acids from the predicted Japanese eel leptin1.

For the other gene, named here Leptin2 gene, a partial sequence was retrieved from the European eel draft genome (488 bp), while the complete CDS sequence was retrieved from the Japanese eel draft genome. The Japanese leptin2 CDS is a 519 bp sequence, composed by a 150 bp exon1 and a 369 bp exon2. The resulting predicted amino acid sequence is made of 173 aa and contains a 21 amino acid signal peptide. The predicted partial European eel leptin2 differs only by five amino acids from the corresponding sequence of the predicted Japanese eel leptin2 (comparison made on the 163 common amino acids).

#### European eel leptin cDNA cloning

Specific primers were designed from the European and Japanese predicted sequences and PCR analyses were conducted in order to obtain the complete cDNA sequences for leptin1 and leptin2 (S2 Fig). These PCR were conducted on cDNA obtained from European eel tissues. The European eel leptin1 cDNA sequence is identical to the predicted European eel CDS sequence. The European eel leptin2 cDNA sequence differs only by three nucleotides from the corresponding partial sequence characterized in the European eel genome, and sixteen nucleotides from the complete CDS sequence characterized in the Japanese eel genome. The resulting amino acid sequence is identical to the corresponding partial European eel sequence, and still differs by the same five amino acids from the predicted Japanese eel sequence.

### Leptin sequence comparisons

We characterized two leptins (leptin1 and leptin2) in eel species. Two leptins have been described in most other teleosts investigated so far, with a few exceptions, like the fugu, which possess only one leptin [6, 12–16, 33, 46–48]. European eel leptin1 and 2 display 97.7% and 97.1% identity when compared to their respective Japanese eel leptin1 and 2 (S5 Table). This indicates that leptin sequences are highly conserved between the two eel species. In contrast, there is a high divergence between both leptin types, with only 51.4% identity between leptin1 and leptin2 in the European eel (S5 Table).

When compared to human leptin, European eel leptin1 displays 25.7% of sequence identity, and European eel leptin2 23.7% (S5 Table). This low identity is nevertheless higher than between human and fugu, the first non-mammalian leptin described (13.2% identity: [6]). The percentages of identity between eel and other teleost leptins vary for European eel leptin1, from 30.2% (zebrafish leptinA) to 22.7% (fugu leptin), and for European eel leptin2, from 32.4% (zebrafish leptinA) to 25.9% (medaka leptinA). The highest identity for both European eel sequences is found with the single leptin of the non-teleost actinopterygian spotted gar: 47.7% for eel leptin1 and 50.9% for eel leptin2 (S5 Table).

Both eel leptins possess the two highly conserved cysteines, in positions 121 and 171 for European and Japanese eel leptin1, and positions 123 and 173 for European and Japanese eel leptin2 (S2 Fig). This disulphide bridge is required for full biological activity, as demonstrated in human [49]. Its high conservation in eel, as in all vertebrate leptins characterized so far, suggests a conservation of the mechanisms of activation of their specific receptors.

### Leptin phylogenetic analyses

Two leptins were identified in each of the two eel species. This is in agreement with the presence of two leptin paralogs (“A” and “B” types) in some teleosts, such as zebrafish and medaka, while some other teleosts, such as fugu and striped bass, possess only one leptin gene [6, 12, 13, 16]. We retrieved only a single leptin gene in a non-teleost actinopterygian, the spotted gar, and in a basal sarcopterygian, the coelacanth, as in tetrapods. Previous studies have suggested that the teleost leptin “A” and “B” paralogs may have arisen from the specific teleost whole genome duplication (3R), while the “B” type would have been lost in some percomorphs [12, 13, 16]. In addition, the salmonid-specific genome tetraploidization (4R) further duplicated the number of leptin genes in salmonids (*i*.*e*. four leptin genes in Atlantic salmon) [34, 50]. The tetraploidization of the carp genome, also resulted in an additional duplication of the leptin genes, with two leptinA being present in carp species, such as the common carp, *Cyprinus carpio* [46], and the Jian carp, *Cyprinus carpio var*. *Jian* [51]. In the latter, a single leptinB has also been identified [51].

**Fig 1:**
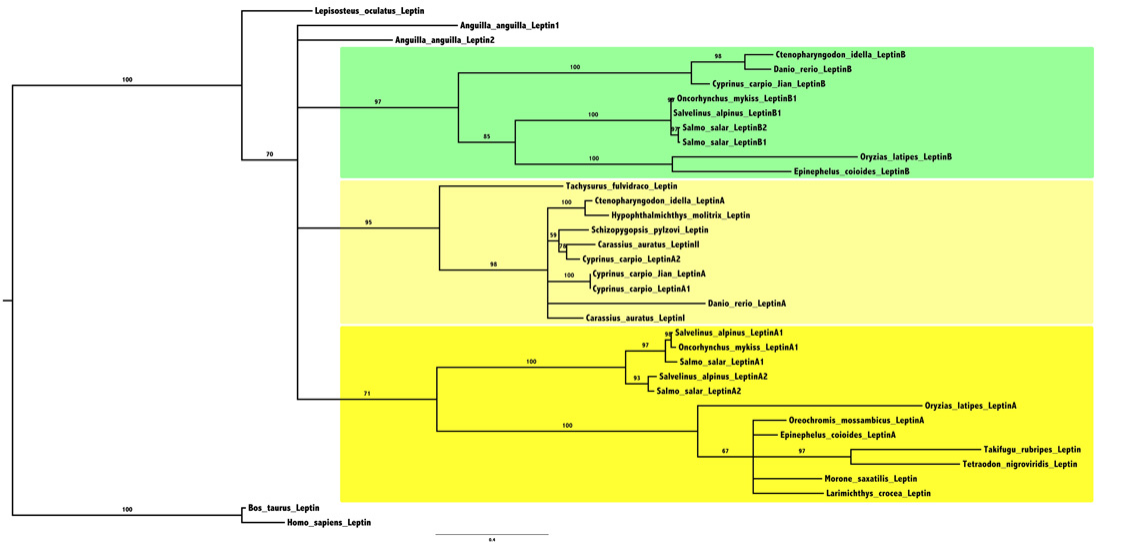
Consensus phylogenetic tree of actinopterygian leptins. Phylogenetic analysis of 34 actinopterygian leptin amino acid sequences was performed using the Maximum Likelihood method, with 1,000 boostrap replicates (for the alignment and references of sequences see S3 Fig and S1 Table). The number shown at each branch node indicates in percentage the boostrap value. Only values above 50% are indicated. The tree was rooted using mammalian (human and bull) leptin sequences as outgroup. Teleost duplicated leptin groups are indicated in yellow (“A” type) and in green (“B” type).

To characterize the two European eel leptins among teleost leptins, we performed phylogenetic analyses on 34 actinopterygian leptin amino acid sequences (33 leptins from teleost species and one from a non-teleost species, the spotted gar), with leptins from two mammalian species as outgroup (Fig 1 and S3 Fig). In the phylogenetic tree (Fig 1), generated using the Maximum Likelihood method, the single spotted gar leptin branches basal to all teleost leptins. Teleost leptinB sequences cluster in one well-supported clade, while teleost leptinA sequences were distributed in two clades, one encompassing silurid and cyprinid sequences, and the other salmonid and percomorph sequences. Previous phylogenetic analyses [12, 13, 16, 50] also did not show well-supported separation of teleost leptin sequences into a single “A” and a single “B” clades. Our phylogenetic tree suggests that this may result from a rapid divergence between leptinA sequences from teleost sub-groups. The two eel leptin sequences branch independently from the “A” and “B” teleost clades, which does not allow classifying eel leptins as “A” or “B” types. The leptin phylogenetic tree displays shorter branch lengths for spotted gar leptin and the two eel leptins, as compared to the longer branch lengths for the other teleost leptins. This suggests that the two eel leptins may have less rapidly evolved after the 3R duplication than the leptins of the other teleosts. More data in representative species from basal groups of teleosts may improve the phylogenetic analysis and help in classifying eel leptins. Another way to further classify eel leptins was to perform synteny analyses.

### Leptin synteny analysis

**Fig 2:**
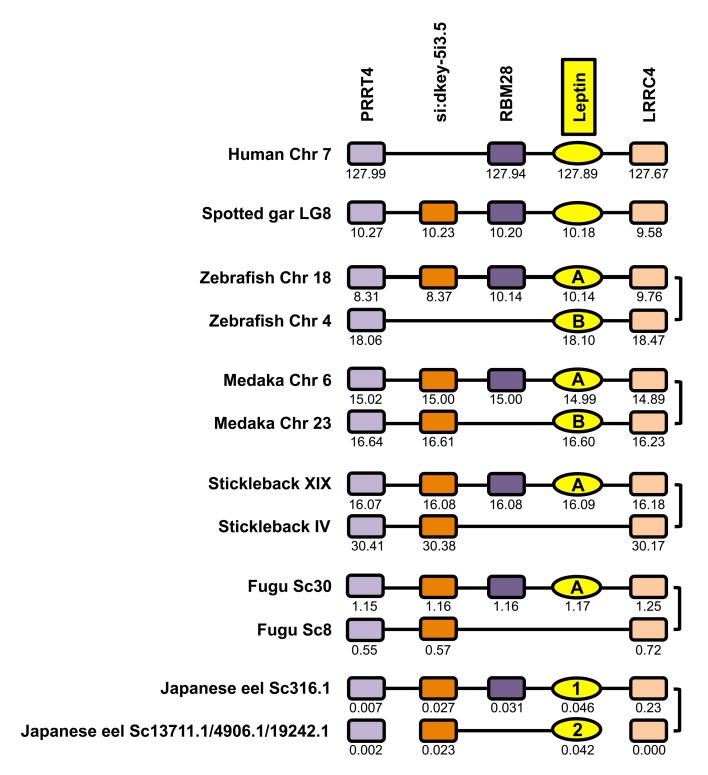
Conserved genomic synteny of vertebrate leptins. Genomic synteny maps comparing leptins and their neighbouring genes from human, non-teleost actinopterygian (spotted gar), and teleost species including the two eel leptins (leptin1 and leptin2) genomic regions, are represented. The leptin genomic region has been duplicated in teleost species, likely as a result of the teleost specific third round of genome duplication. The duplicated leptins have been conserved in most of the teleosts, including the eel. In the current status of the European and Japanese eel draft genomes, the identification of a “A” or a “B” type for eel leptins would be based only on the presence of a RBM28 on the same scaffold as eel leptin1. Therefore, we chose to keep a distinct nomenclature in the present study, and named the eel leptins, eel leptin1 and leptin2. Genes are named after their human orthologs according to the Human Genome Naming Consortium (HGNC), except for si:dkey-5i3.5 that does not exist in human. This gene was named after its spotted gar ortholog. Orthologs of each gene are represented in the same color and displayed in the same column. The genes reproduced in this figure are not necessarily presented in the same order as they appear on the chromosomes and scaffolds, except for spotted gar, and their positions are indicated in 10^6^ base pairs. The detailed genomic locations of the genes are given in Supporting S6 Table.

We compared eel leptin genomic regions with homologous regions in genomes of a sarcopterygian (Human), a non-teleost actinopterygian (spotted gar), and teleosts (zebrafish, medaka, stickleback, fugu, Japanese eel) (Fig 2 and S6 Table). Only four leptin neighboring genes could be identified in the eel, due to the small size of the leptin scaffolds in the European and Japanese eel draft genomes, *i*.*e*. PRRT4, si:dkey-5i3.5, RBM28, and LRRC4. These four genes are located in the leptin genomic regions of all the vertebrate species investigated in this study, supporting the orthology of the vertebrate leptin genes.

In teleosts, previous leptin synteny analyses showed that this genomic region has been duplicated as a result of the third whole genome duplication event that occurred specifically in this lineage (3R) [12, 13, 16]. The genomic regions of the two eel leptins display duplicated copies of PRRT4, si:dkey-5i3.5, and LRRC4, as in the other teleost investigated in this study, with the exception of a single si:dkey-5i3.5 in zebrafish (Fig 2). In contrast, a single copy of these three genes is present in the spotted gar genome. This supports that the duplication of eel leptin genomic region resulted from 3R, as for the other teleost species.

In the eel, a single copy of RBM28 is conserved and located in the same scaffold as eel leptin1 (Fig 2). In the other teleosts, only one RMB28 paralog is also present and located in the genomic region of leptinA. This suggests that eel leptin1 may be orthologous to the teleost leptinA. To test this hypothesis, we performed phylogenetic analyses of the leptin neighboring genes. PRRT4, si:dkey-5i3.5, and LRRC4 phylogenetic analyses strengthen the hypothesis that, in teleosts, the genomic regions encompassing leptinA and leptinB resulted from 3R (S4–S6 Figs). However, these phylogenetic analyses could not classify eel duplicated genes into separate teleost clades, and therefore could not contribute to support the orthology of eel leptin1 and teleost leptinA genomic regions. In the current status of the European and Japanese eel draft genomes, the identification of a “A” or a “B” type for eel leptins would be based only on the presence of a single RBM28 on the same scaffold as eel leptin1. Therefore, we chose to keep a distinct nomenclature in the present study, and named the eel leptins, eel leptin1 and leptin2.

### Characterization of eel LEPR

#### European and Japanese LEPR partial gene prediction

Two partial sequences of LEPR genes were identified in each of the European and Japanese eel draft genomes. For one LEPR gene, named here LEPRa, a partial sequence of 3053 bp was characterized in the European and Japanese eel draft genomes. This LEPRa partial sequence is made of 16 exons. The missing part mainly corresponds to the 5’ end. Further BLAST analyses did not succeed to identify the 5’ end of the sequences in the two eel draft genomes.

For the second LEPR gene, named here LEPRb, a partial sequence of 2639 bp was characterized in the European and Japanese eel draft genomes. This LEPRb partial sequence is made of 14 exons. As for LEPRa, the missing part corresponds mainly to the 5’ end, and additional BLAST analyses could not identify it in the two eel draft genomes.

#### Cloning of the cDNAs of the two European eel LEPRs and complete gene structure prediction

Specific primers were designed from the European and Japanese partial predicted sequences (S4 Table) and PCR analyses were conducted in order to obtain corresponding cDNA sequences for LEPRa and LEPRb.

**Fig 3:**
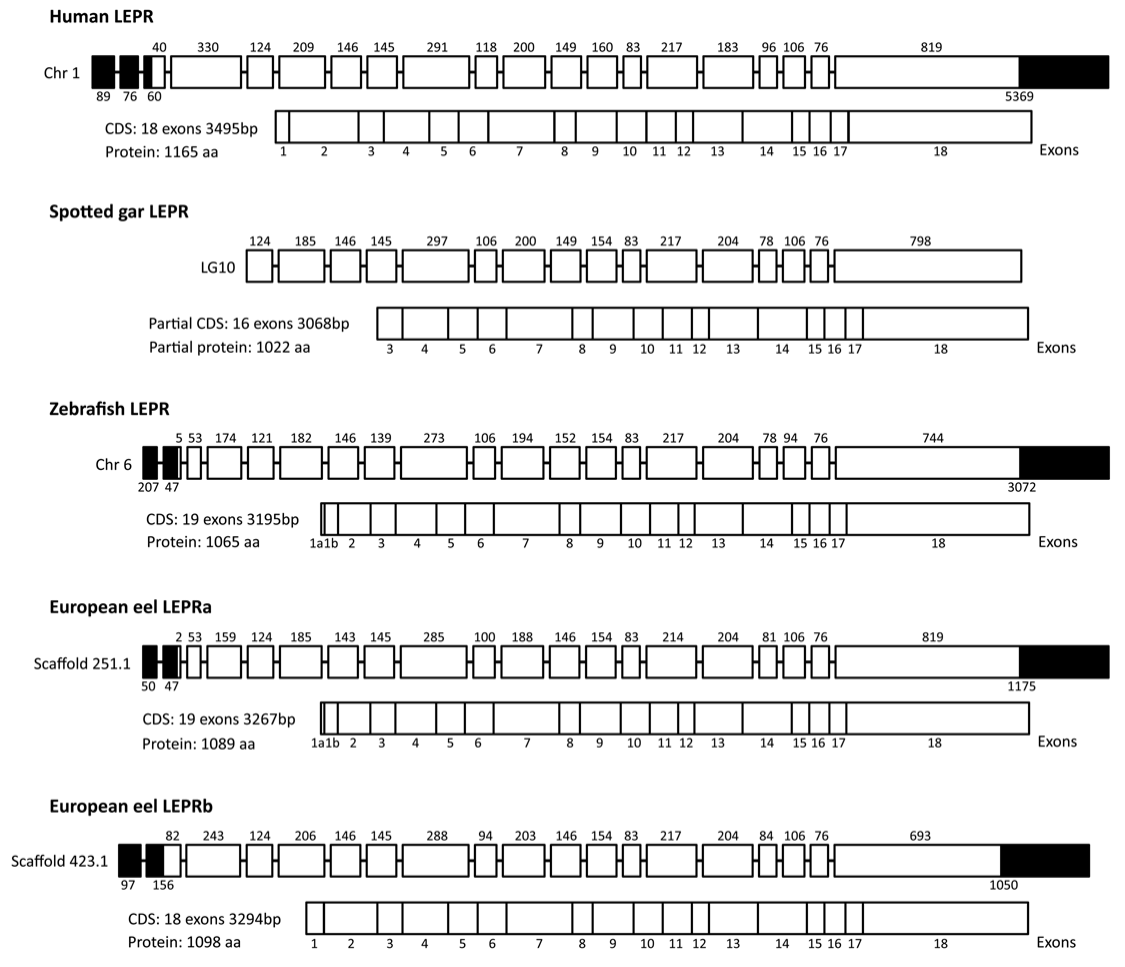
Comparison of leptin receptor (LEPR) gene structure, in human and actinopterygian species. LEPR genes from human, non-teleost actinopterygian spotted gar, and teleost species including the two eel LEPRs are represented. Exons are symbolized either by black squares (untranslated nucleotides) or white squares (translated nucleotides). The nucleotide length is indicated above each translated exon. The total lengths of exons containing untranslated nucleotides are indicating below. The numbers of the exons (from 1 to 18) composing the coding sequence (CDS) are indicated below. Chr: chromosome; LG: linkage group.

For LEPRa, we initially amplified a 2911 bp sequence. This sequence displays 99.6% identity with the corresponding partial sequence predicted in the European eel genome. Specific RACE PCR primers (S4 Table) were designed to conduct 5’ and 3’ RACE PCR in order to obtain the complete LEPRa cDNA (S7A Fig). We could amplify a 3700 bp sequence, comprised of 20 exons, with 19 exons coding for a 3267 bp CDS (Fig 3 and S7A Fig). The resulting predicted protein is composed of 1089 aa, with a 22 aa signal peptide, a 765 aa extracellular segment, a single 23 aa transmembrane domain, and a 301 aa intracellular segment (Fig 3 and S7A Fig).

For LEPRb, we initially amplified a 2287 bp sequence. This sequence displays 98% identity with the corresponding partial sequence predicted in the European eel genome. Specific RACE PCR primers (S4 Table) were designed to conduct 5’ and 3’ RACE PCR. We could amplify a 3822 bp sequence, constituted by 19 exons, with 18 exons coding for a 3294 bp CDS (Fig 3 and S7B Fig). The resulting predicted protein is composed of 1098 aa, with a 31 aa signal peptide, a 816 aa extracellular segment, a single 23 aa transmembrane domain, and a 259 aa intracellular segment (Fig 3 and S7B Fig).

### LEPR sequence comparisons

We characterized two LEPR in each of the two eel species. This is the first report of duplicated leptin receptors in vertebrates. The two European eel LEPR predicted proteins contain the characteristic domains of the cytokine receptor superfamily, conserved among vertebrate LEPRs, as predicted by Interproscan software [2] (S7 Fig). The extracellular part of each LEPR displays a putative leptin binding domain (LBD), a Ig-like C2-type domain, and three fibronectin III domains, two of them containing the WSXWS signature. The cytoplasmic region contains two JAK and one STAT boxes. These boxes are involved in the JAK/STAT pathway characteristic of the leptin signaling.

The amino acid sequences of European eel LEPRa and LEPRb display 98.9% and 98.3% identity when compared to Japanese eel LEPRa and b, respectively (S7 Table). This indicates that LEPR sequences are highly conserved between the two eel species. In contrast, there is a high divergence between both LEPR types, with only 49% identity between LEPRa and LEPRb in the European eel.

When compared to human LEPR, European LEPRa displays 30.3% of sequence identity, and European eel LEPRb 29.3%. The percentages of identity between eel and other teleost LEPR vary for European eel LEPRa, from 43.7% (salmon) to 33.7% (medaka), and for European eel LEPRb, from 41.2% (salmon) to 31.7% (medaka) (S7 Table).

In order to compare eel LEPR sequences with that of a non-teleost actinopterygian, we predicted a longer sequence of LEPR in the spotted gar genome (LepOcu1), from BLAST analyses. We used as queries the zebrafish and Atlantic salmon LEPR, in addition to the two eel LEPR identified in this study, and the partial spotted gar LEPR sequence from the Ensembl Genome Database. We were able to identify a partial 3068 bp LEPR sequence made of 16 exons and localized on the chromosome LG10 (Fig 3 and S8 Fig). The first predicted exon corresponds to the exon 3 of the CDS of human LEPR, zebrafish LEPR, European eel LEPRa and LEPRb (Fig 3). Our predicted spotted gar LEPR partial sequence is longer than the 2501 bp annotated sequence in Ensembl, and includes 4 additional exons in the partial 5’ end of the sequence.

As our predicted spotted gar LEPR sequence is still partial, we restricted the comparison to the corresponding partial sequences of vertebrate LEPR, including European eel LEPRa and LEPRb (S8 Table). The two European eel LEPR display the highest of sequence identity with the spotted gar LEPR: 53.2% for LEPRa and 47% for LEPRb.

### LEPR phylogenetic analyses

**Fig 4:**
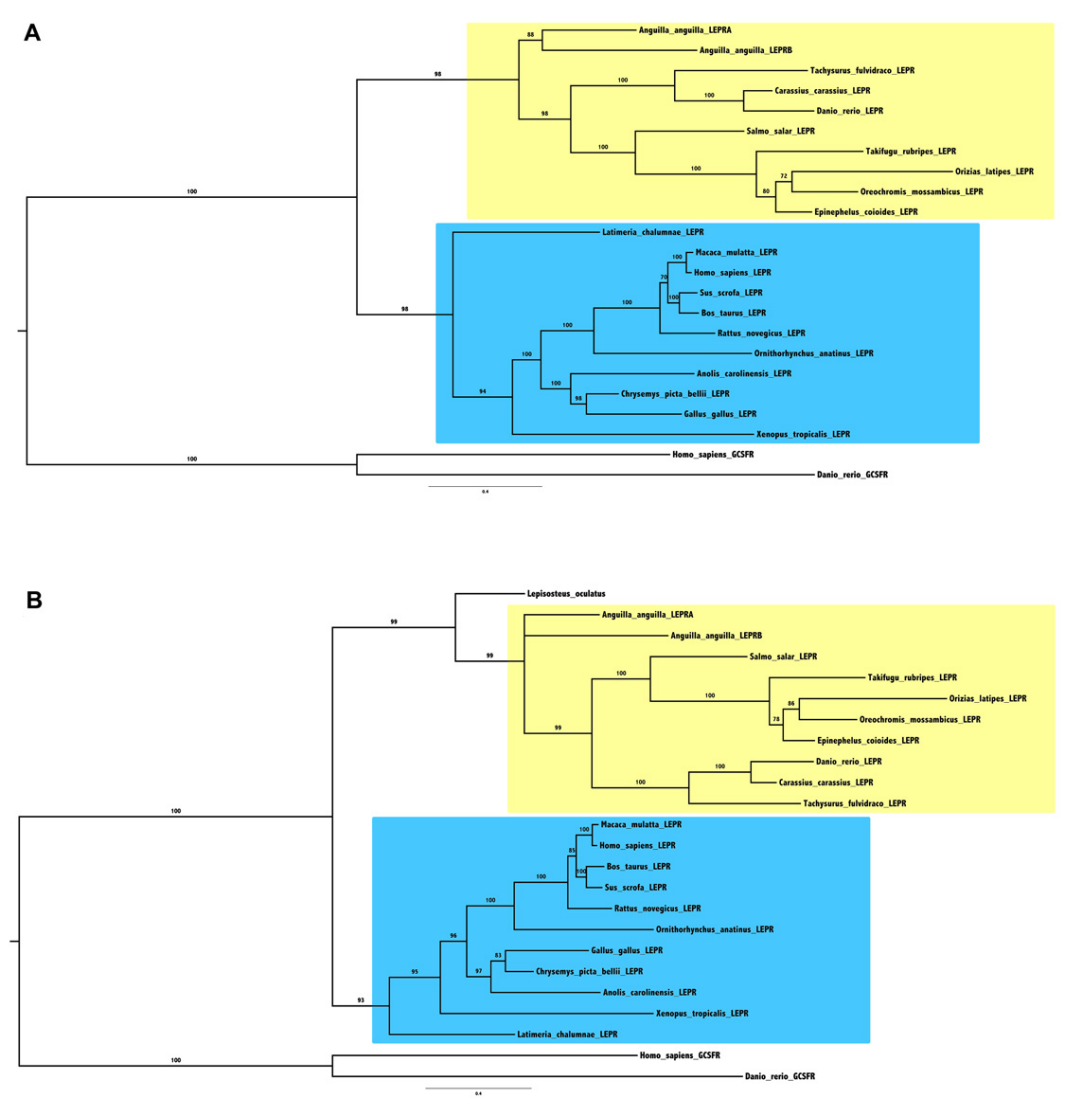
Consensus phylogenetic trees of vertebrate leptin receptors (LEPRs). Phylogenetic analyses of 21 vertebrate LEPR amino acid sequences (Fig 4A) and 22 partial vertebrate LEPR amino acid sequences (Fig 4B) were performed using the Maximum Likelihood method, with 1,000 boostrap replicates (for the alignments and references of sequences, see S9 and S10 Figs, and S2 Table). The number shown at each branch node indicates, in percentage, the boostrap value. The trees were rooted using human and zebrafish GCSFR sequences as outgroups. Teleost LEPR group is indicated in yellow and sarcopterygian LEPR group is indicated in blue.

To better understand the evolutionary history of the LEPR genes, we performed phylogenetic analyses on various vertebrate LEPR sequences. Based on a 21 vertebrate LEPR amino acid sequence alignment (S9 Fig), and assuming human and zebrafish GCSFR (Granulocyte colony-stimulating factor receptor) sequences as outgroup, a phylogenetic tree was generated using the Maximum Likelihood method (Fig 4A). This analysis clusters the LEPR sequences in two clades, one encompassing the sarcopterygian species and the second one encompassing the teleost species. The two European eel LEPR sequences form a group branching at the base of the teleost clade, which may suggest that the two eel LEPR resulted from a lineage-specific duplication in the eels. As the spotted gar predicted LEPR sequence is incomplete, we did not include it in this alignment. However, we proceeded with an additional alignment including to spotted gar, with all sequences shortened to the same size, in order to avoid artificial bias (S10 Fig). This phylogenetic tree (Fig 4B) shows that the single spotted gar LEPR branches basal to all teleost LEPR, and that the two eel LEPR branch independently at the base of the other teleost sequences. This tree displays shorter branch lengths for spotted gar LEPR and the two eel LEPR, suggesting that they are less divergent than the other teleost LEPR. This tree is compatible with two hypotheses: duplicated eel LEPR may originate either from teleost 3R, or from a specific gene-duplication, that could have occurred in Elopomorphs or Anguillids. In the 3R-origin hypothesis, the second LEPR gene would have been lost after the elopomorph emergence.

### LEPR synteny analysis

**Fig 5:**
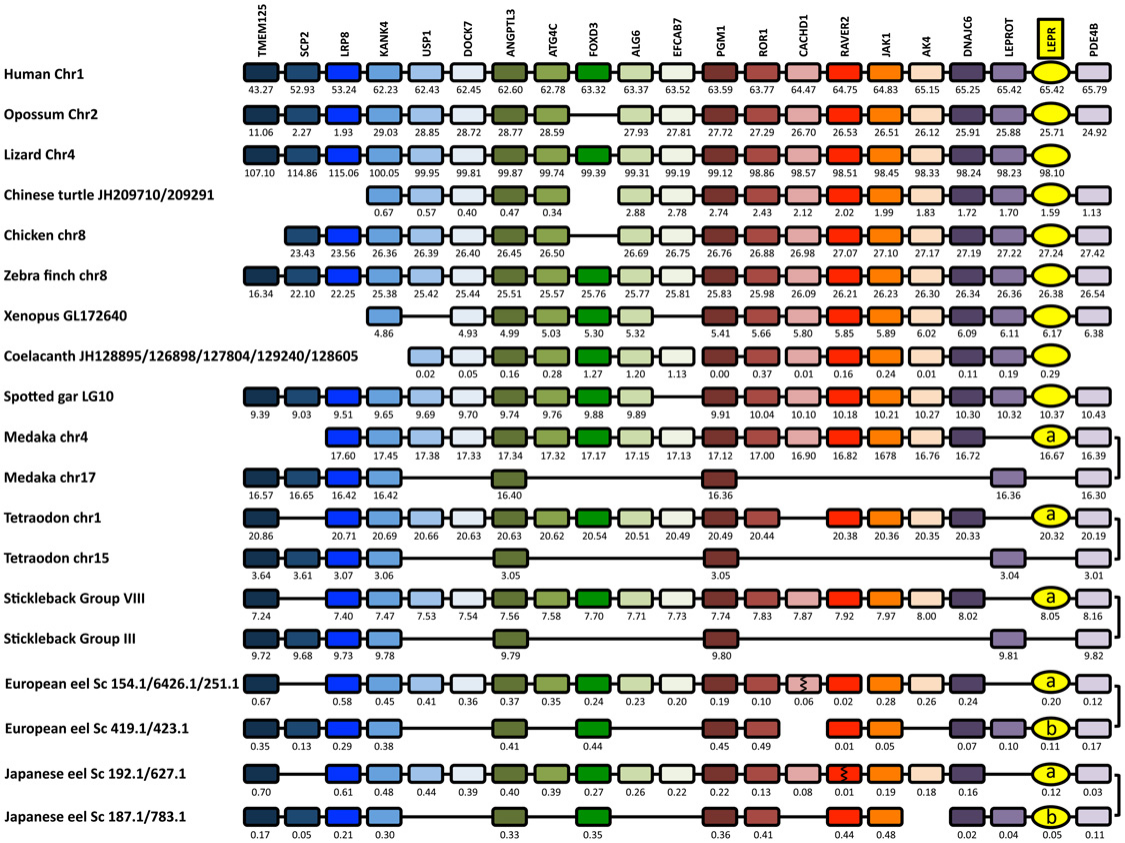
Conserved genomic synteny of vertebrate leptin receptors (LEPR). Genomic synteny map comparing LEPR and their neighbouring genes from human, other tetrapods, basal sarcoterygian (coelacanth), non-teleost actinopterygian (spotted gar), and teleost species including the two eel LEPR (LEPRa and LEPRb) genomic regions, are represented. The LEPR genomic region has been duplicated in teleost species, likely as a result of the teleost specific third round of genome duplication. The duplicated LEPRb has been conserved in the eel but not in the other teleosts. Genes are named after their human orthologs according to the Human Genome Naming Consortium (HGNC). Orthologs of each gene are represented in the same color and displayed in the same column. The genes reproduced in this figure are not necessarily presented in the same order as they appear on the chromosomes and scaffolds, except for human, and their positions are indicated in 10^6^ base pairs. The detailed genomic locations of the genes are given in Supporting S9 Table.

To further resolve the origin of the two eel LEPR, and confront our two hypotheses, we performed a synteny analysis of the LEPR neighboring genes in several vertebrate genomes: eutherian mammal (human), prototherian mammal (opossum), birds (chicken, zebra finch, *Taeniopygia guttata*), squamate (lizard), chelonian (Chinese turtle), amphibian (Western clawed frog), basal sarcopterygian (coelacanth), non-teleost actinopterygian (spotted gar) and teleosts (medaka, tetraodon, stickleback, European eel, Japanese eel) (Fig 5 and S9 Table).

In the eel genomes, we characterized two LEPR genes. We could reconstruct, by manually combining scaffolds, their genomic environment containing the following neighboring genes: TMEM125, SCP2, LRP8, KANK4, USP1, DOCK7, ANGPTL3, ATG4C, FOXD3, ALG6, EFCAB7, PGM1, ROR1, CACHD1, RAVER2, JAK1, AK4, DNAJC6, LEPROT, and PDE4B (Fig 5). These genes are located in the LEPR genomic regions of human and most vertebrate species investigated in this study, supporting the orthology of the vertebrate LEPR genes.

Many of these neighboring genes are duplicated in the eels, as in the other teleosts investigated in these study, medaka, tetraodon, and stickleback, which all display duplicated copies of TMEM125, LRP8, KANK4, ANGPTL3, PGM1, and PDE4B (Fig 5). In contrast, the spotted gar possesses only a single copy of these genes (Fig 5). This supports the hypothesis that LEPR genomic region has been duplicated in teleosts, as a result of 3R.

Differently from the eels, we identified the presence of only one LEPR gene in these duplicated regions in the other teleosts investigated, suggesting the loss of a duplicated LEPR after elopomorph emergence. By BLAST analyses, we assessed the absence of the duplicated LEPR in the rest of the genomes of these species. The chromosome in which the duplicated LEPR is missing, also shows a loss of many other duplicated genes, such as USP1, DOCK7, ATG4C, FOXD3, ALG6, EFCAB7, ROR1, CACHD1, RAVER2, JAK1, AK4, DNAJC6, in these three teleost species. Conversely, some other duplicated genes have been lost on the LEPR containing chromosome, such as SCP2 and LEPROT (Fig 5).

As for the medaka, stickleback and tetraodon LEPR, the genomic region of eel LEPRa (scaffolds 251.1/6426.1/154.1 of the European eel genome and scaffolds 627.1/192.1 of the Japanese eel genome) contains USP1, DOCK7, ATG4C, FOXD3, ALG6, EFCAB7, ROR1, CACHD1, RAVER2, JAK1, AK4, DNAJC6, and lacks SCP2 and LEPROT. This suggests that eel LEPRa is orthologous to the other teleost LEPR.

The genomic region of eel LEPRb (scaffolds 423.1/419.1 of the European eel genome and scaffolds 783.1/187.1 of the Japanese eel genome) has conserved LEPROT and SCP2. As mentioned above, these two genes are only conserved in the chromosome lacking LEPR in medaka, stickleback and tetraodon. This supports that the orthologous gene of eel LEPRb would have been lost in the other teleosts. It can be noticed that, in addition to LEPRb, the eel has conserved more duplicated genes in this region than the other teleosts, such as FOXD3, ROR1, RAVER2, JAK1, DNAJC6. This is in agreement with our recent observations that the eel genome may have conserved more duplicated genes after the 3R event than other teleosts [38, 39, 52, 53].

We tested the hypothesis of a 3R-origin of LEPR genomic region in teleosts by performing phylogenetic analyses of LEPR neighboring gene families (S11–S17 Figs). Concerning LEPR neighboring genes duplicated in all teleosts studied (S11–S15 Figs), phylogenetic trees show two well-supported clades for teleost-specific paralogs. The single spotted gar gene always branches basal to teleost sequences (S11–S17 Figs). These features support a 3R-origin of the LEPR genomic region. However, various situations are observed in the case of the eel paralogs. LRP8 phylogenetic tree clearly shows that each eel duplicated gene branches basal to the respective teleost-specific duplicate cluster, which further supports a 3R-origin of the genomic region (S11 Fig). The same situation is displayed in the case of KANK4 (S12 Fig). Concerning TMEM125, one duplicated eel gene, located on the LEPRb paralogon, branches basal to its respective teleost-specific cluster (S13 Fig). The other eel TMEM125 branches basal to all teleost sequences, which may reflect a low divergence (S13 Fig). A similar situation is obtained for ANGPTL3 (S14 Fig). Concerning PDE4B, both eel genes branch independently and basal to the two teleost-specific duplicate clusters (S15 Fig). Concerning DNAJC6, duplicated only in eels, both eel sequences are basal to the other teleost sequences. Bootstrap values strongly support that the eel DNAJC6 located on LEPRa paralogon, would be the paralog of the single gene conserved in the other teleosts, which is also located on the LEPRa paralogon (S16 Fig). A similar situation is displayed for JAK1 (S17 Fig). Altogether, phylogeny and synteny analyses of LEPR neighboring genes are in favor of a 3R-origin for the LEPR duplicated genomic region. This supports that the two eel LEPR do not result from an eel specific gene-duplication, but from the conservation of the duplicated copies originated from the teleost whole genome duplication 3R.

### Differential tissue distributions of leptins and LEPRs in the eel

Among extant vertebrate species, eels show the largest leptin system, with two leptins and two leptin receptors. Conservation of multiple genes may reflect differential functions. We compared their expression in various tissues, in order to get some insights on their potential differential roles in the eel. We developed specific qPCR assays for European eel leptin1, leptin2, LEPRa and LEPRb. We specifically assessed that there was no cross reaction in the qPCR between duplicated leptins nor between duplicated LEPRs.

#### Wide tissue distribution of eel leptins and LEPRs

**Fig 6:**
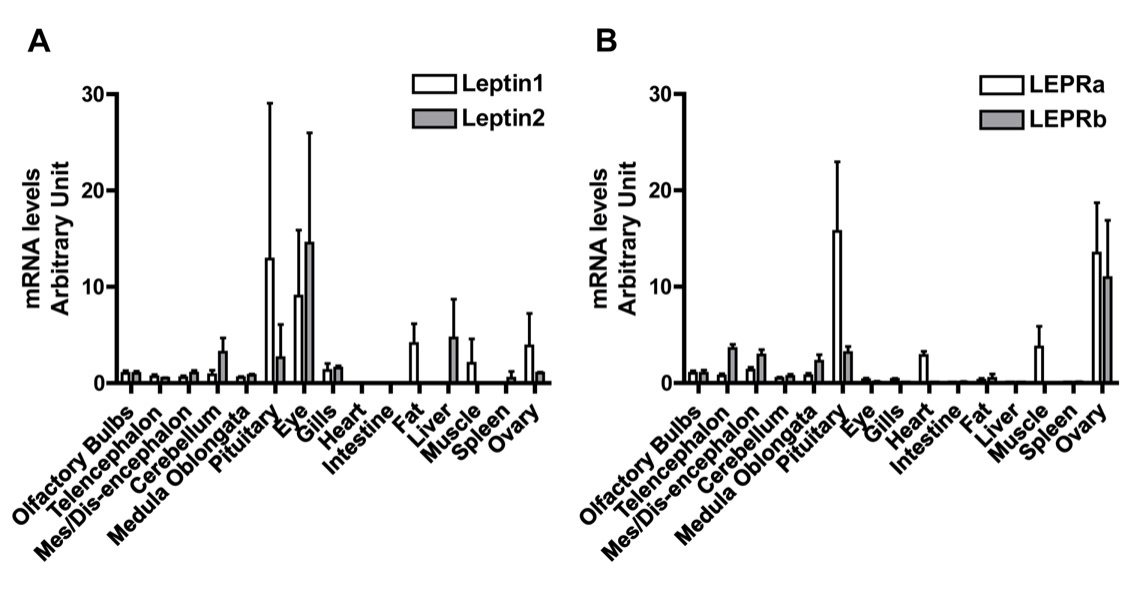
Tissue distribution of the expression of the two leptins and the two leptin receptors (LEPRs) in the European eel. Messenger RNA levels for eel leptins 1 and 2 (Fig 6A) and eel LEPR A and B (Fig 6B) were measured by qPCR and normalized to the amount of total RNA. Each bar represents the mean ± SD from 8 pre-pubertal female eels.

Both leptins are expressed in the five brain parts, as well as in the pituitary, eye, gills, and ovary (Fig 6A). The highest expression is found in the pituitary and in the eye. In contrast, we can highlight that only leptin1 is expressed in the adipose tissue, while only leptin2 is expressed in the liver. This indicates a differential expression of the two leptins concerning these two major organs involved in metabolism. No expression could be detected in some tissues, such as heart and intestine.

Both leptins are highly expressed in the eel eye. Expression in the eye has also been reported for other teleost species, such as Atlantic salmon, medaka, or goldfish [13, 33, 34]. In mammals, leptin appears to be involved in some eye diseases. In human, elevated vitreous leptin levels can be measured in the eyes of diabetic retinopathy patients [54]. In the pig, leptin is expressed in the eye and is upregulated in case of ocular inflammation [55]. The high expression of leptin in the eye of teleosts may provide relevant models to investigate the function of leptin in this organ.

LEPRa and LEPRb also show a wide tissue distribution. Both LEPRs are expressed in the five brain parts, pituitary, eye, adipose tissue and ovary (Fig 6B). The highest expression is in the pituitary and in the ovary for LEPRa and in the ovary for LEPRb. In contrast, only LEPRa is expressed in heart, muscle, liver, and gills, while LEPRb expression is under the limit of detection in these tissues. No expression could be detected for either LEPR in some tissues, such as spleen and intestine. Similarly to the situation described in mammals (for review: [5, 19, 23]), LEPR in teleosts often shows an ubiquist tissue distribution, suggesting multiple physiological functions, such as fat metabolism, reproduction, cardiovascular functions or immunity (for review [22]).

In the eel, only LEPRa is expressed in the heart, with no expression of both leptins, nor LEPRb. Leptin would thus act in the eel as an endocrine factor on the heart, through its interaction with LEPRa. In other teleosts, such as fugu, Atlantic salmon, goldfish, catfish (*Pelteobagrus fulvidraco)*, and tilapia (*Oreochromis niloticus)*, LEPR is also highly expressed in heart [33–35, 56–58]. In mammals, leptin has been described as a cardiac hypertrophic factor potentially involved in heart failure [59], and this hormone can represent a potential link between obesity and cardiovascular risk [59, 60]. The expression of LEPR in the heart of teleosts may suggest an ancient role of leptin in this organ among osteichthyans.

#### Differential distribution of eel leptins in the liver and adipose tissue

We demonstrated the selective expression of eel leptin1 in the fat tissue and of eel leptin2 in the liver. Mammalian leptin was discovered for its role in lipid metabolism and adipostat, with the adipose tissue constituting its main expression site [1]. In contrast, the first identified teleost leptin (fugu), was shown to be expressed mainly in the liver [6]. Since then, many reports in teleosts have highlighted the high expression of leptin in the liver [6, 12–15, 33, 34, 46–48, 50, 51, 56–58, 61, 62]. This hepatic expression of leptin is likely related to the major role played out by the liver in lipid storage and metabolism in teleosts [63].

In amphibians, leptin expression has been reported in both fat and liver in African clawed frog [8], whereas in salamander, leptin was only expressed in fat [7]. In birds, the recently characterized leptin has been found in both fat and liver in rock dove (*Columba livia*) [11], whereas in zebra finch neither of these two tissues were shown to express it [10].

Since its discovery in non-mammalian vertebrates, liver was thought to be the main expression site of leptin in teleosts, whereas in tetrapods leptin would be expressed in both fat and liver [22]. However, recent studies demonstrated the expression of leptin in fat tissues in teleosts, including this present study in the eel [14, 34, 47, 50, 56, 57]. This suggests that the expression of leptin in both liver and adipose tissues may represent an ancient character shared by actinopterygians and sarcopterygians. In the eel, this function may be partitioned between the duplicated leptins, depending on the type of tissue involved, leptin1 being expressed in the adipose tissue, while leptin2 in the liver. This differential localization suggests a potential subfunctionalization of the two leptins in the eel, which may have driven the conservation of the two genes in this species.

We also showed the expression of leptin1, but not leptin2, in the muscle, as in the fat tissue. Leptin function in muscle may be related to its role in energy storage and allocation in teleosts, as suggested in zebrafish and salmonids [32, 34]. In this organ LEPRa was also highly expressed, while LEPRb could not be detected. The local action of leptin in muscle may occur specifically through LEPRa in the eel. In the same way, the receptor orthologous to eel LEPRa was also shown to be highly expressed in muscle in various teleosts (medaka: [13]; Atlantic salmon: [34, 58]; goldfish: [33]; catfish: [56]; tilapia: [57])

#### Expression of eel leptins and LEPRS in the BPG axis

In the eel, the two leptins and the two LEPRs are expressed in the brain, pituitary and gonads, indicating potential endocrine/paracrine roles of the leptin system on the BPG axis. In various teleosts species, LEPR expression is high in the brain, pituitary and gonads, suggesting a function of leptin in reproduction, as we will discuss further in the following section [13, 14, 32–35, 56–58].

In mammals, the leptin system has been described as a major actor in puberty and reproduction (for review: [5, 19, 20]. *Obese* gene (leptin) deficient female mice are infertile, and ovulation can be stimulated in these females by recombinant leptin treatments [64]. In mammals, leptin can stimulate the BPG axis at different levels, by inducing GnRH release from the hypothalamus, likely via the activation of Kisspeptin neurons expressing LEPR [65, 66], by inducing gonadotropin (LH and FSH) release from the pituitary [67], and also by directly activating the gonads [68, 69].

The expression of leptins and leptin receptors at the various levels of the BPG axis in the eel, suggest an ancient and conserved pleiotropic role of the leptin system in the reproductive function in osteichthyans. Future studies, using *in situ* hybridization and immunocytochemistry, should aim at identifying the cells expressing leptin and LEPR along the BPG axis in teleosts.

### Differential regulation of leptins and LEPRs during fasting and experimental maturation in the eel

Tissue distributions showed that the leptin system is expressed in major tissues involved in the regulation of metabolism and reproduction, such as brain, pituitary, ovary and liver. In order to further investigate the role of eel duplicated leptins and LEPRs, we analyzed the regulation of their expression during experimental maturation.

A first experimental maturation (Exp1) was performed on two groups, one control group sacrificed at the beginning of the experiment, and one mature group sacrificed at the end of experimental maturation. As eels stop feeding from the silver stage, experimental maturation was performed on fasting eels. In order to discriminate between the effect of fasting and the effect of maturation, a second experiment (Exp2) was performed on three groups: one initial control group, one control group sacrificed at the end of the experiment, and one group of matured eels.

#### No impact of long-term fasting on eel leptin system

Remarkably, there is no significant difference in the expression of leptin1, leptin2, LEPRa and LEPRb mRNA in the brain, pituitary, liver and ovary, between the control eels sacrificed at the beginning and at the end of the experimental maturation, after four months of fasting (Exp2). This suggests that the leptin system in these tissues is not significantly regulated in relation with long-term fasting in silver eels.

In mammals, leptin is a main actor in the regulation of metabolism, body weight and appetite. Leptin has an inhibitory effect on orexigenic peptides, such as neuropeptide Y (NPY), melanine-concentrating hormone (MCH) and orexins, and a stimulatory effect on anorexigenic peptides, such as proopiomelanocortin (POMC), alpha-melanocyte-stimulating hormone (alpha-MSH), corticotropin-releasing hormone (CRH) and cholecystokinin (CCK) [70]. In this way, high leptin levels inhibit appetite and feeding behavior, while low leptin levels stimulate them [70].

In the teleost species, for which an anorexigenic function of leptin has been highlighted, the pathways involved might be similar to those described in mammals, such as POMC and NPY pathways. In the rainbow trout (*Oncorhynchus mykiss*), treatment with homologous recombinant leptin induced a decrease in the food intake concomitant with a decrease of NPY and an increase in POMC mRNA levels in the brain [61]. In medaka, a recent study has demonstrated the existence in the brain of an appetite-signaling pathway mediated by LEPR [71]. In this species, LEPR knockout induced an increase in food intake, concomitant with an increase in the expression of the orexigenic NPYa and a decrease in the expression of the anorexigenic POMC1 [71].

However, in teleosts, the role of leptin on appetite and feeding behavior remains controversial as different status have been reported, depending on the species [46, 48, 58, 72–75]. For instance, in the common carp, 6 weeks of fasting had no impact on hepatic leptin mRNA levels [46]. In the same way, 8 week-overfed zebrafish presented no variation in their visceral adipose tissue leptin mRNA expression [72]. In both cases there were significant modifications in the body mass of the fish. In contrast, in some species, variation in leptin expression has been, positively or negatively, correlated to the feeding status of the fish. In the Arctic charr (*Salvelinus alpinus*), 10 weeks of fasting induced an increase in both hepatic mRNA and plasma levels of leptin [48]. Similar results were observed in another salmonid, the Atlantic salmon [58]. In the flounder, *Paralichthys adspersus*, 3 weeks of fasting induced a significant increase in the leptin plasma levels [73]. In contrast, in the green sunfish, *Lepomis cyanellus*, and the burbot, *Lota lota*, 2 weeks of fasting induced a decrease in the leptin plasma levels [74, 75].

The variation in leptin regulation in relation to fasting or overfeeding in teleosts is thus highly dependent on the species. This may be related to the great variability observed among this group concerning life cycles, development, environmental conditions, and reproduction [76]. From our results, we suggest that the leptin system may not be involved in the long term fasting of the silver eels. This might be related to the particular metabolism of the silver eels. In fact, previous studies showed that silver eel metabolism and energy expenditure are remarkably adapted to long term fasting, which they experience throughout their oceanic migration, sexual maturation and reproduction [40, 77].

#### Differential impact of sexual maturation on the expression of eel leptins and LEPRs

**Fig 7:**
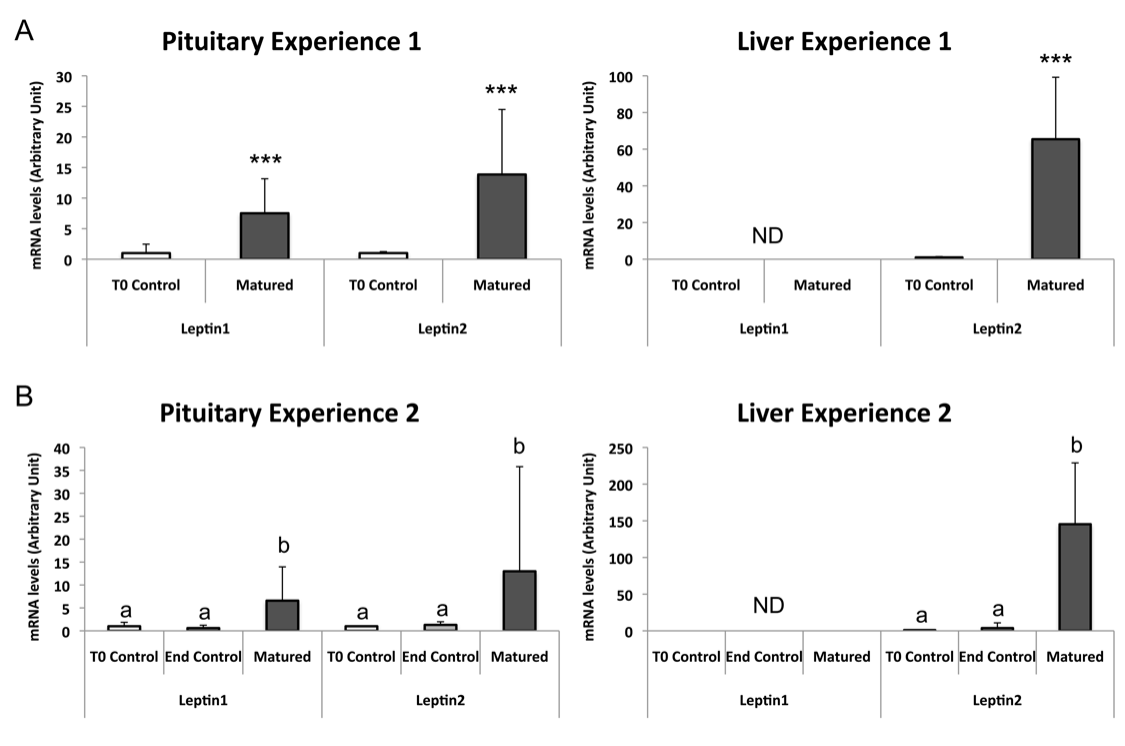
Regulation of the expression of the two eel leptins in the pituitary and liver during experimental maturation. The mRNA levels of eel leptin1 and 2 were measured by qPCR and normalized to beta-actin mRNA. (Fig 7A) Experiment1: Each bar represents the mean ± SD from 10 control female eels sacrificed at the beginning of the experiment (T0 controls) and 10 matured female eels sacrificed at the end of the experiment. Significant differences between the two eel groups were analyzed by Mann-Whitney *U* test; ***P<0.001. (Fig 7B) Experiment2: Each bar represents the mean ± SD from 6 control females sacrificed at the beginning of the experiment (T0 controls), 6 control females sacrificed at the end of the experiment (End controls), and 6 matured females. Significant differences between the three eel groups were analyzed by Kruskal-Wallis ANOVA; significant differences are indicated by different letters. ND: Not detectable.

Concerning leptin, in both experiments major changes were observed in the pituitary and liver of matured eels (Fig 7A and 7B). In the pituitary, the expression of both leptin1 and leptin2 mRNA is significantly upregulated as compared to initial controls for Exp 1 (leptin1: x7.5; n = 10/group; *P*<0.0001; leptin2: x13.8; n = 10/group; *P*<0.0001) and for Exp 2 (leptin1: x6.6; n = 6/group; H = 11.661; *P*<0.05; leptin2: x13.1; n = 6/group; H = 10.246; *P*<0.05). In the liver, the expression of leptin1 transcript remains under the limit of detection in matured eels, as in controls, in both experiments. In contrast, a striking increase in the expression of leptin2 mRNA is observed in the liver of matured eels (x65; n = 10/group; *P*<0.0001; and x145; n = 6/group; H = 11.474; *P*<0.05; as compared to initial controls for Exp 1 and 2, respectively). In the other tissues (anterior brain and ovary) no significant variation of leptin1 and leptin2 expression is observed in matured eels. These data indicate that both leptins are differentially regulated according to the tissue. Furthermore, these regulation studies reinforce the specific role of leptin2 expressed by the liver, with a strong increase during the maturation process.

The liver is at the center of the metabolism challenge involved in teleost sexual maturation. In addition to its major function in energy storage, this organ produces the vitellogenin in oviparous vertebrates [78]. This phospho-lipo-glyco-protein, released in the circulation, is incorporated into the oocytes to form the yolk, crucial source of nutrition for the embryonic development and the larvae survival [78, 79]. The hepatic vitellogenin production, under the control of sex steroids, costs high-energy expenditure for the females. In the eel, the sexual maturation, including the vitellogenesis, occurs during the oceanic migration while animals are fasting [40, 77]. Therefore, all the energy needed for the migration, the metabolism and the sexual maturation will come from the energy storage accumulated during the juvenile growth phase. In mammals, leptin is one major factor involved in the mobilization of energy storage [5]. In teleosts, this role has been suggested in some species such as the goldfish [80], the grass carp (*Ctenopharyngodon idella*) [81], the Arctic charr [82], or the Atlantic salmon [83]. The important upregulation of hepatic leptin2 mRNA expression during sexual maturation observed in this study suggests that this hormone may play such a role in the eel during the oceanic migration and reproduction.

**Fig 8:**
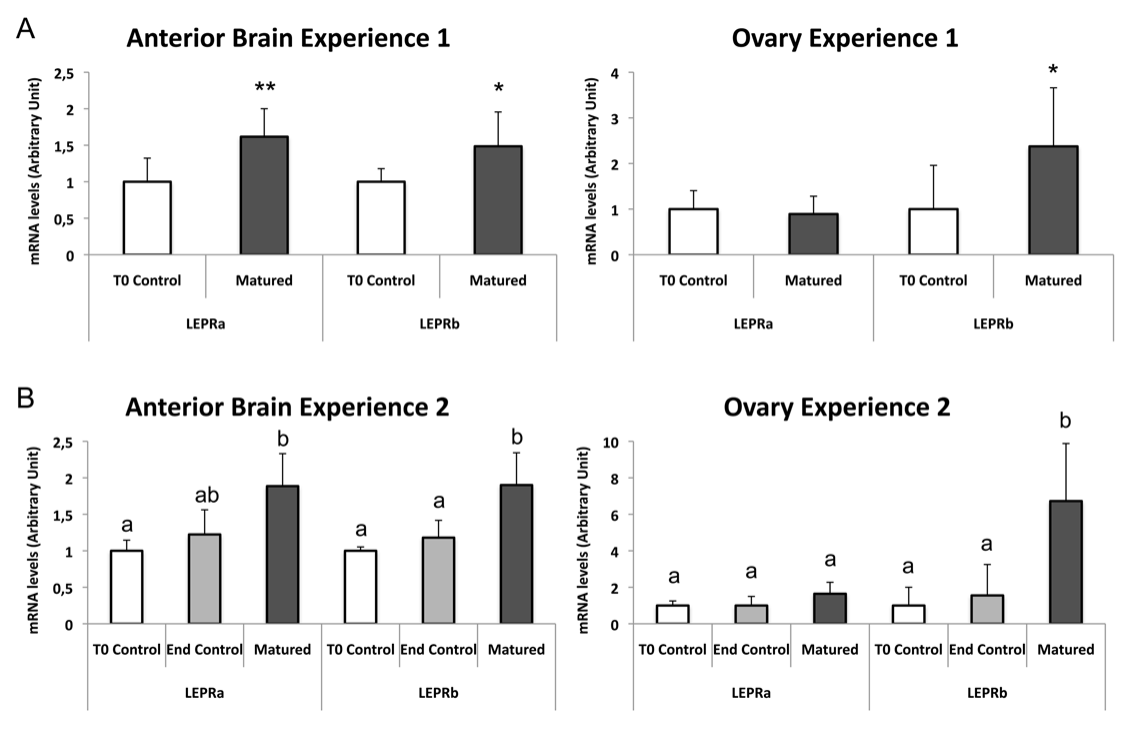
Regulation of the expression of the two eel leptin receptors (LEPRs) in the anterior brain and ovary during experimental maturation. The mRNA levels of eel LEPRa and b were measured by qPCR and normalized to beta-actin mRNA. (Fig 8A) Experiment 1: Each bar represents the mean ± SD from 10 control female eels sacrificed at the beginning of the experiment (T0 controls) and 10 matured female eels sacrificed at the end of the experiment. Significant difference between control and matured groups were analyzed by Mann-Whitney *U* test; **P<0.05 and *P<0.05. (Fig 8B) Experiment 2: Each bar represents the mean ± SD from 6 control females sacrificed at the beginning of the experiment (T0 controls), 6 control females sacrificed at the end of the experiment (End controls), and 6 matured females. Significant differences between each group were analyzed by Kruskal-Wallis ANOVA.

Concerning leptin receptors, in both experiments significant changes were observed in the anterior brain and ovary of matured eels (Fig 8A and 8B), while no significant variations were observed in the pituitary and liver. Both LEPR mRNA expressions are upregulated in the same way in the anterior brain as compared to initial controls for Exp 1 (LEPRa: x1.61; n = 10/group; *P* = 0.0021; LEPRb: x1.48; n = 10/group; *P* = 0.0288) and for Exp 2 (LEPRa: x1.88; n = 6/group; H = 9.93; *P*<0.01; LEPRb: x1.89; n = 6/group; H = 12.117; *P*<0.01). In contrast, only LEPRb transcript is significantly upregulated in the ovary (x2.37; n = 10/group; *P* = 0.0232; and x6.72; n = 6/group; H = 11.099; *P*<0.01; as compared to initial controls for Exp 1 and 2, respectively), while no significant change is observed for LEPRa. These data reveal that both LEPR mRNA are differentially regulated according to the tissue. We can also highlight that, while tissue distribution shows a high expression of both receptors in the ovary, only LEPRb is significantly upregulated during experimental maturation. The differential regulation of the expression of the two eel LEPR suggests differential roles in the reproductive function. This may have contributed to the conservation of the duplicated LEPR genes in this species.

An increase in leptin expression with the onset of sexual maturation has recently been shown in the Atlantic salmon [84]. In the present study, we could demonstrate a tissue and gene-specific increase of the expression of the duplicated leptins and LEPRs in the BPG-liver axis in experimentally matured eels. This suggests a conserved function of the leptin system during reproduction in teleosts. This may reflect an ancient and conserved positive role of the leptin system in the reproductive function in vertebrates, as first discovered in mammals [5, 19, 20, 22].

*In vitro* studies showed a stimulatory effect of mammalian leptin on LH and FSH production or release by pituitary cells, in the sea bass (*Dicentrarchus labrax*) and rainbow trout [85–87]. So far, most of the studies raised in teleosts on the effect of homologous recombinant leptins focused on the effects on metabolism and feeding behavior, such as in the rainbow trout [61, 88], or in the grass carp [81, 89]. As teleost leptins exhibit a very low percentage of sequence identity, not only when compared with mammalian leptins, but also between teleosts, future studies should investigate *in vivo* and *in vitro* effects of homologous recombinant leptins on the BPG axis. Furthermore, it would be also relevant to compare the effects of the duplicated leptins present in teleosts.

### Evolutionary history of the leptin/LEPR system in osteichthyans

**Fig 9:**
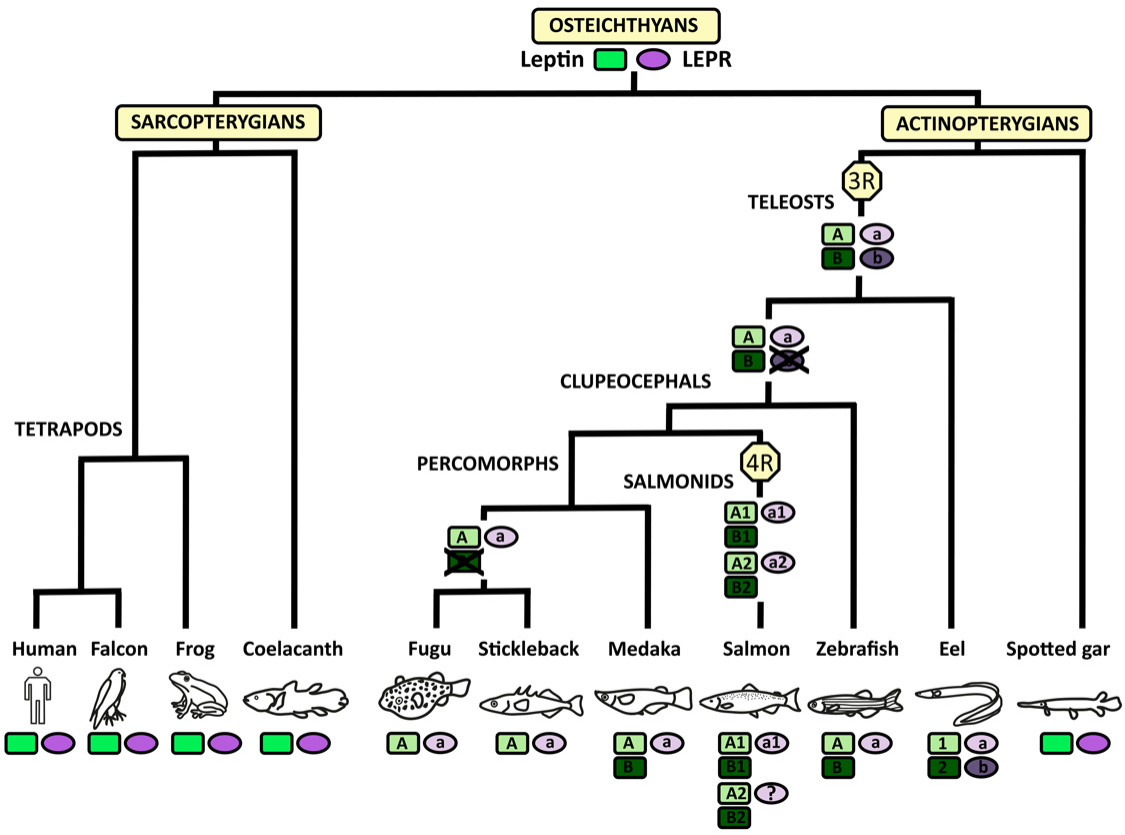
Proposed evolutionary scenario of leptin and LEPR genes in osteichthyans. Osteichthyan ancestor possessed one leptin gene and one LEPR gene. These genes were duplicated in teleosts, as a result of the teleost-specific third whole genome duplication event (3R), and further duplicated in salmonids after the 4R, specific to this group. LEPRb would have been lost during teleost radiation, some time after the elopomorph emergence and before the clupeocephal emergence.

In conclusion, this study brings new insights on the evolutionary history of the leptin system in vertebrates (Fig 9). Leptin and LEPR are present in all the sarcopterygian and actinopterygian species studied so far, suggesting their presence in their common osteichthyan ancestor. All extant sarcopterygians, including a basal representative species, the coelacanth, and various tetrapod groups, possess a single leptin and a single LEPR. Recent advances have assessed the presence of leptin in the avian lineage, making an end to a long-term controversy [9–11]. Similarly, a non-teleost actinopterygian, the spotted gar, also exhibits a single leptin and a single LEPR. This suggests that their common osteichthyan ancestor also possessed a single leptin and a single LEPR. In teleosts, this leptin system would have been duplicated through the teleost-specific third whole genome duplication event (3R). In most extant teleost species, the two duplicated leptin genes originated from 3R are conserved, with some exception like the fugu and the stickleback, which have lost one of the copies. In contrast, eels, representative species of a basal group of teleosts, the elopomorphs, provide so far the unique case of the conservation of the two 3R-duplicated LEPR. The loss of the second LEPR (LEPRb) would have likely occurred during teleost radiation, after the elopomorph emergence. Information on representative species from other basal groups of teleosts, such as osteoglossomorphs [37], would help further pinpoint when the loss of LEPRb occurred. The conservation of the duplicated leptins and LEPRs in the eel may be related to differential roles, as suggested by some variations in their tissue distribution and expression regulation during sexual maturation. Further studies will aim at producing eel recombinant leptins and leptin receptors, and investigating binding specificities as well as possible heterodimerization of the duplicate receptors. Due to their specific phylogenetic position, eels are not only the first but maybe one of the rare examples of the presence of duplicated LEPRs resulting from the teleost 3R. However, multiple genes may also be present in some other extant teleosts, such as salmonids and some carps, as a result of their specific tetraploidization (4R). Four leptins were thus identified in the Atlantic salmon [34, 50] but no parallel information has been published so far concerning LEPR. Considering that the loss of the 3R-duplicated LEPRb likely preceded the emergence of clupeocephales, a maximum of two LEPR (LEPRa1 and LEPRa2) could be expected in carps and salmonids after the 4R events. Eels would thus still be some of the rare extant species having conserved LEPRb.

## Supporting Information

S1 Fig Comparison of leptin gene structure in human and actinopterygian species. Leptin genes from human, non-teleost actinopterygian spotted gar, and teleost species, including the two eel leptins are represented. Leptin genes are all composed by two small exons (symbolized by white squares) separated by an intron (symbolized by a line). The nucleotide length is indicated above each symbol. The number of each exon is indicated below. CDS: coding sequence; Chr: chromosome; LG: linkage group.
(PDF)

S2 Fig European eel leptin1 (LN558789) and leptin2 (LN558790) cDNA nucleotide sequences and predicted amino acid sequences. Exon1 is boxed in a continued line, while exon2 is boxed in a discontinued line. Signal peptide is shaded in black. The two cysteines, conserved among vertebrates, are shaded in red.
(PDF)

S3 Fig Alignment of 37 amino acid sequences of leptins used for the phylogenetic analysis. The sequences were aligned by Clustal Omega and manually adjusted. The amino acids presenting similar physico-chemical properties are shaded in the same color.
(PDF)

S4 Fig Consensus phylogenetic tree of vertebrate LRRC4. Phylogenetic analysis of 29 vertebrate LRRC4 amino acid sequences was performed using the Maximum Likelihood method, with 1,000 boostrap replicates (for the references of sequences, see S3 Table). The number shown at each branch node indicates in percentage the boostrap value. Only values above 50% are indicated. The tree was rooted using human and zebrafish LRRC4b sequences as outgroup. Sarcopterygian LRRC4 group is indicated in blue, teleost duplicated LRRC4 groups are indicated in green (type 1) and yellow (type 2).
(PDF)

S5 Fig Consensus phylogenetic tree of vertebrate PRRT4. Phylogenetic analysis of 21 vertebrate PRRT4 amino acid sequences was performed using the Maximum Likelihood method, with 1,000 boostrap replicates (for the references of sequences, see S3 Table). The number shown at each branch node indicates in percentage the boostrap value. Only values above 50% are indicated. The tree was rooted using human and zebrafish PRRT3 sequences as outgroup. Sarcopterygian PRRT4 group is indicated in blue, teleost duplicated PRRT4 groups are indicated in yellow (“A” type) and green (“B” type).
(PDF)

S6 Fig Consensus phylogenetic tree of actinopterygian si:dkey-5i3.5. Phylogenetic analysis of 17 actinopterygian si:dkey-5i3.5 amino acid sequences was performed using the Maximum Likelihood method, with 1,000 boostrap replicates (for the references of sequences, see S3 Table). The number shown at each branch node indicates in percentage the boostrap value. Only values above 50% are indicated. The tree was rooted using human and zebrafish TMEM53 sequences as outgroup. Teleost duplicated si:dkey-5i3.5 groups are indicated in yellow (“A” type) and green (“B” type).
(PDF)

S7 Fig European eel LEPRa (LN558791) (A) and LEPRb (LN558792) (B) cDNA nucleotide sequences and predicted amino acid sequences. Signal peptide is shaded in black. Transmembrane domain is shaded in green. Ig-like C2-type domain is shaded in light blue. Fibronectin type 3 domains are shaded in red. WSXWS motifs are indicated in blue letters. The leptin binding site is boxed. JAK box are shaded in purple. STAT box is shaded in yellow.
(PDF)

S8 Fig Partial predicted spotted gar LEPR nucleotide and amino acid sequences. (PDF)

S9 Fig Alignment of 21 amino acid sequences of LEPR and 2 sequences of GCSFR used for the phylogenetic analysis. The sequences were aligned by Clustal Omega and manually adjusted. The amino acids presenting similar physico-chemical properties are shaded in the same color.
(PDF)

S10 Fig Alignment of 22 amino acid sequences of LEPR and 2 sequences of GCSFR used for the phylogenetic analysis. The sequences were aligned by Clustal Omega and manually adjusted. The amino acids presenting similar physico-chemical properties are shaded in the same color.
(PDF)

S11 Fig Consensus phylogenetic tree of vertebrate LRP8. Phylogenetic analysis of 31 vertebrate LRP8 amino acid sequences was performed using the Maximum Likelihood method, with 1,000 boostrap replicates (for the references of sequences, see S3 Table). The number shown at each branch node indicates in percentage the boostrap value. Only values above 50% are indicated. The tree was rooted using human and zebrafish VLDLR sequences as outgroup. Sarcopterygian LRP8 group is indicated in blue, teleost duplicated LRP8 groups are indicated in yellow (“A” type) and green (“B” type).
(PDF)

S12 Fig Consensus phylogenetic tree of vertebrate KANK4. Phylogenetic analysis of 26 vertebrate KANK4 amino acid sequences was performed using the Maximum Likelihood method, with 1,000 boostrap replicates (for the references of sequences, see S3 Table). The number shown at each branch node indicates in percentage the boostrap value. Only values above 50% are indicated. The tree was rooted using human and zebrafish KANK1 sequences as outgroup. Sarcopterygian KANK4 group is indicated in blue, teleost duplicated KANK4 groups are indicated in yellow (“A” type) and green (“B” type).
(PDF)

S13 Fig Consensus phylogenetic tree of actinopterygian TMEM125. Phylogenetic analysis of 23 actinopterygian TMEM125 amino acid sequences was performed using the Maximum Likelihood method, with 1,000 boostrap replicates (for the references of sequences, see S3 Table). The number shown at each branch node indicates in percentage the boostrap value. Only values above 50% are indicated. The tree was rooted using sarcopterygian (human, lizard and coelacanth) TMEM125 sequences as outgroup. Teleost duplicated TMEM125 groups are indicated in yellow (“A” type) and green (“B” type).
(PDF)

S14 Fig Consensus phylogenetic tree of vertebrate ANGPTL3. Phylogenetic analysis of 30 vertebrate ANGPTL3 amino acid sequences was performed using the Maximum Likelihood method, with 1,000 boostrap replicates (for the references of sequences, see S3 Table). The number shown at each branch node indicates in percentage the boostrap value. Only values above 50% are indicated. The tree was rooted using human and zebrafish ANGPTL4 sequences as outgroup. Sarcopterygian ANGPTL3 group is indicated in blue, teleost duplicated ANGPTL3 groups are indicated in yellow (“A” type) and green (“B” type).
(PDF)

S15 Fig Consensus phylogenetic tree of vertebrate PDE4B. Phylogenetic analysis of 23 vertebrate PDE4B amino acid sequences was performed using the Maximum Likelihood method, with 1,000 boostrap replicates (for the references of sequences, see S3 Table). The number shown at each branch node indicates in percentage the boostrap value. Only values above 50% are indicated. The tree was rooted using human and zebrafish PDE4A sequences as outgroup. Sarcopterygian PDE4B group is indicated in blue, teleost duplicated PDE4B groups are indicated in yellow (“A” type) and green (“B” type).
(PDF)

S16 Fig Consensus phylogenetic tree of vertebrate DNAJC6. Phylogenetic analysis of 20 vertebrate DNAJC6 amino acid sequences was performed using the Maximum Likelihood method, with 1,000 boostrap replicates (for the references of sequences, see S3 Table). The number shown at each branch node indicates in percentage the boostrap value. Only values above 50% are indicated. The tree was rooted using human and zebrafish GAK sequences as outgroup. Sarcopterygian DNAJC6 group is indicated in blue, teleost DNAJC6 group is indicated in yellow.
(PDF)

S17 Fig Consensus phylogenetic tree of vertebrate JAK1. Phylogenetic analysis of 25 vertebrate JAK1 amino acid sequences was performed using the Maximum Likelihood method, with 1,000 boostrap replicates (for the references of sequences, see S3 Table). The number shown at each branch node indicates in percentage the boostrap value. Only values above 50% are indicated. The tree was rooted using human and zebrafish JAK2 sequences as outgroup. Sarcopterygian JAK1 group is indicated in blue, teleost JAK1 group is indicated in yellow.
(PDF)

S1 Table References of the leptin amino acid sequences used in the phylogenetic analysis (Fig 1). (XLSX)

S2 Table References of the LEPR amino acid sequences used in the phylogenetic analyses (Fig 4A and 4B). (XLSX)

S3 Table References of the leptin and LEPR neighboring gene amino acid sequences used in the phylogenetic analyses (S4–S6 and S11–S17 Figs). (XLSX)

S4 Table European eel gene specific primers. (DOCX)

S5 Table Comparison of leptin amino acid sequences. (DOCX)

S6 Table Names, references and locations of the genes used in the leptin synteny analysis (Fig 2). (XLSX)

S7 Table Comparison of LEPR amino acid sequences. (DOCX)

S8 Table Comparison of partial LEPR amino acid sequences. (DOCX)

S9 Table Names, references and locations of the genes used in the LEPR synteny analysis (Fig 5). (XLSX)

S10 Table The ARRIVE Guidelines Checklist for Animal Research. (PDF)
