# Supplementary material for: Duplicated Leptin Receptors in Two Species of Eel Bring New Insights into the Evolution of the Leptin System in Vertebrates
Source: PLoS One. 2015 May 6;10(5):e0126008. doi: 10.1371/journal.pone.0126008 (PMC4422726; doi:10.1371/journal.pone.0126008)
Supplement: S1 Fig — Leptin genes from human, non-teleost actinopterygian spotted gar, and teleost species, including the two eel leptins are represented. Leptin genes are all composed by two small exons (symbolized by white squares) separated by an intron (symbolized by a line). The nucleotide length is indicated above each symbol. The number of each exon is indicated below. CDS: coding sequence; Chr: chromosome; LG: linkage group. (PDF) [file pone.0126008.s001.pdf]

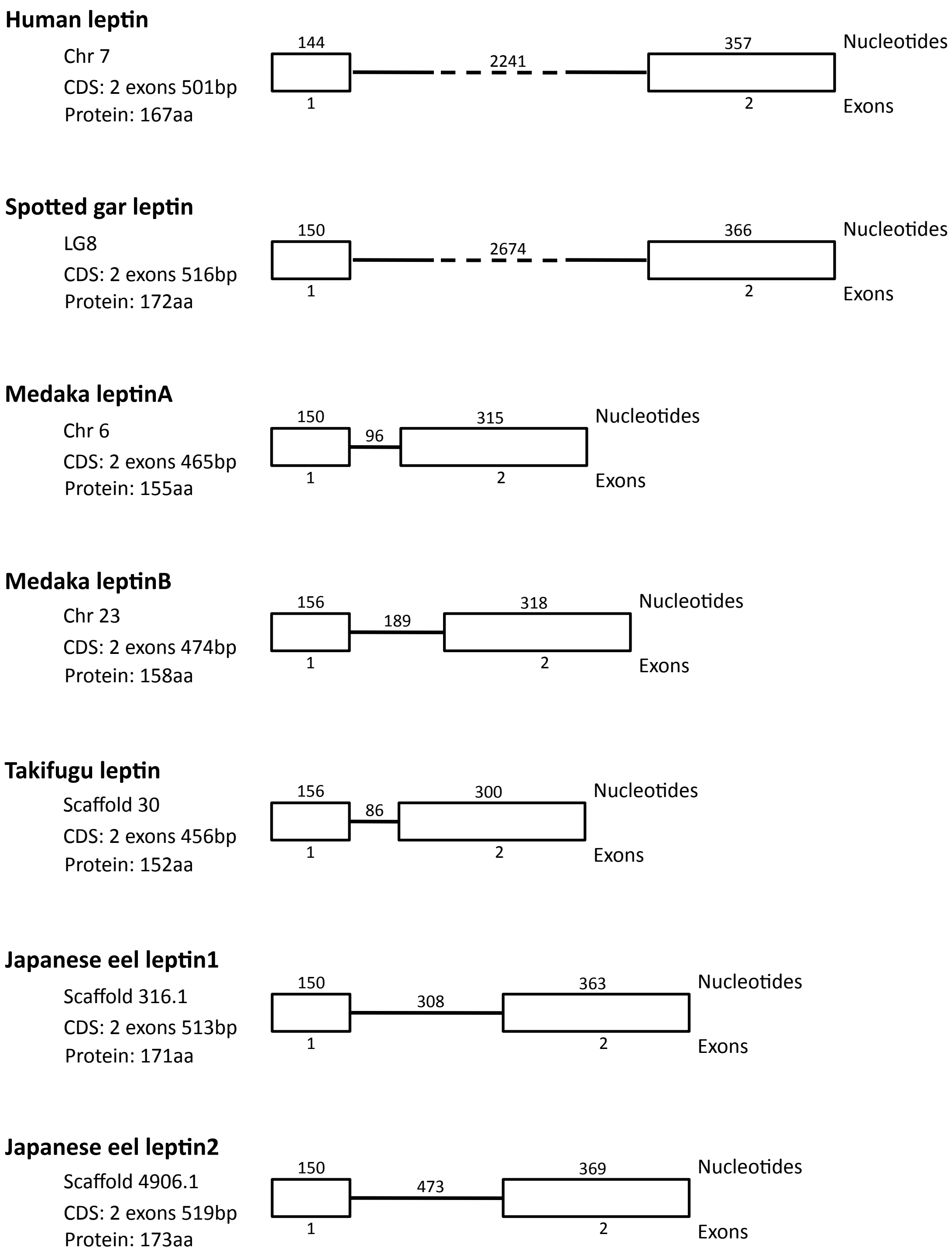
Human leptin
Chr 7
CDS: 2 exons 501bp
Protein: 167aa
144
2241
357
Nucleotides
1
2
Exons
Spotted gar leptin
LG8
CDS: 2 exons 516bp
Protein: 172aa
150
2674
366
Nucleotides
1
2
Exons
Medaka leptinA
Chr 6
CDS: 2 exons 465bp
Protein: 155aa
150
96
315
Nucleotides
1
2
Exons
Medaka leptinB
Chr 23
CDS: 2 exons 474bp
Protein: 158aa
156
189
318
Nucleotides
1
2
Exons
Takifugu leptin
Scaffold 30
CDS: 2 exons 456bp
Protein: 152aa
156
86
300
Nucleotides
1
2
Exons
Japanese eel leptin1
Scaffold 316.1
CDS: 2 exons 513bp
Protein: 171aa
150
308
363
Nucleotides
1
2
Exons
Japanese eel leptin2
Scaffold 4906.1
CDS: 2 exons 519bp
Protein: 173aa
150
473
369
Nucleotides
1
2
Exons
