# Supplementary material for: Duplicated Leptin Receptors in Two Species of Eel Bring New Insights into the Evolution of the Leptin System in Vertebrates
Source: PLoS One. 2015 May 6;10(5):e0126008. doi: 10.1371/journal.pone.0126008 (PMC4422726; doi:10.1371/journal.pone.0126008)
Supplement: S2 Fig — Exon1 is boxed in a continued line, while exon2 is boxed in a discontinued line. Signal peptide is shaded in black. The two cysteines, conserved among vertebrates, are shaded in red. (PDF) [file pone.0126008.s002.pdf]

Fig S1

>Anguilla anguilla Leptin1

```
  1 – ATGCATCACTTCATCATTCTTCACTGCACTTCCCTCCTCTTTCTACTGACAATGGGTAAAAGCACACCGCCCCCTGTG
  1 -  M  H  H  F  I  I  L  H  C  T  S  L  L  F  L  L  T  M  G  K  S  T  P  P  P  V
 79 – GACAAAATGAAGAACAACGTCAAAATGCTAGGAGAAACAGCATTAATACGGATACAAAAATTCACGAATGAGTTCCAG
 27 -  D  K  M  K  N  N  V  K  M  L  G  E  T  A  L  I  R  I  Q  K  F  T  N  E  F  Q
157 – ATTTCTCCTAACATGGTGTTCAGCGGTGCAGAGTTGATCCCCAACATCACGCTGGAGACACCCCTGGGTTTGTCCTCC
 53 -  I  S  P  N  M  V  F  S  G  A  E  L  I  P  N  I  T  L  E  T  P  L  G  L  S  S
235 – GTAGCGGAGAACCTCAACACTTTCCAGCTCATTTTGCTGAACCTGACCCTGGACGGCACGCTCCAAATCCGCAGTGAC
 79 -  V  A  E  N  L  N  T  F  Q  L  I  L  L  N  L  T  L  D  G  T  L  Q  I  R  S  D
313 – ATTGTAGGCCTCCTGGACATTGTGCACTGGCTAGCAGCCTCCAGTAGCTGCCCCATGAAGAAACCAGCAAGTGACGGA
105 -  I  V  G  L  L  D  I  V  H  W  L  A  A  S  S  S  C  P  M  K  K  P  A  S  D  G
391 – CACTTGGAAACTTTTCTCAAGACCAACATGCCTTTTCAACTCTCAATTGCCAACATTGTCTTGACCCGACTACAGGAG
131 -  H  L  E  T  F  L  K  T  N  M  P  F  Q  L  S  I  A  N  I  V  L  T  R  L  Q  E
469 - TTCTTAAACAAACTAATCAACAACCTAGACCAGCTGAAAAAGTGTTGA
157 -  F  L  N  K  L  I  N  N  L  D  Q  L  K  K  C  *
```

>Anguilla anguilla Leptin2

```
  1 - ATGTCCGGCTGCGTGGCGCTCCTCTGCACCTCCCTCCTGGTGCTCCTGCCCTTGGGAGCGGGGGTGCCCCTCTCCGTG
  1 -  M  S  G  C  V  A  L  L  C  T  S  L  L  V  L  L  P  L  G  A  G  V  P  L  S  V
 79 - GAGACCATGAAGAGTAACGTCAAGCTGATGGCGCAGACCACCATCGTCAGGATACAGAAGCTCACAGAGGAGTTCCGG
 27 -  E  T  M  K  S  N  V  K  L  M  A  Q  T  T  I  V  R  I  Q  K  L  T  E  E  F  R
157 – ATATCCCCCAACATGGTGTTCAGCGGCCTGGAGCTGATCCCGGACATCGCCCCGGACAAGGCGTGGGAGGGCCTGTCG
 53 -  I  S  P  N  M  V  F  S  G  L  E  L  I  P  D  I  A  P  D  K  A  W  E  G  L  S
235 - GCCATCGCGCAGGGGCTGCACTCCTTCCAGGTGGTCCTGTCCCACCTGCCGCCGGGCGACGGCATGGCGCAGGTGCAC
 79 -  A  I  A  Q  G  L  H  S  F  Q  V  V  L  S  H  L  P  P  G  D  G  M  A  Q  V  H
313 - GCCGATGTCCTGAGCCTCCACGGCGTGGTGCGCTCGCTCGCCGCCTCGCTCGGCTGCCCGCTGCACAAGCCCGCCGGC
105 -  A  D  V  L  S  L  H  G  V  V  R  S  L  A  A  S  L  G  C  P  L  H  K  P  A  G
391 - GACGGCCGCCTGGAGGCCTTCCTCAAGACCAACTCCACCTTCCACGTCACCATCGGCAACGTGGCCCTGGAGAGGCTC
131 -  D  G  R  L  E  A  F  L  K  T  N  S  T  F  H  V  T  I  G  N  V  A  L  E  R  L
469 - CGGCGCTTCCTCGGCAAGCTGGTCCAGAACCTGGACCAGCTCAAGAGCTGCTGA
157 -  R  R  F  L  G  K  L  V  Q  N  L  D  Q  L  K  S  C  *
```
