# Supplementary material for: Duplicated Leptin Receptors in Two Species of Eel Bring New Insights into the Evolution of the Leptin System in Vertebrates
Source: PLoS One. 2015 May 6;10(5):e0126008. doi: 10.1371/journal.pone.0126008 (PMC4422726; doi:10.1371/journal.pone.0126008)
Supplement: S3 Fig — The sequences were aligned by Clustal Omega and manually adjusted. The amino acids presenting similar physico-chemical properties are shaded in the same color. (PDF) [file pone.0126008.s003.pdf]

```
                               1
Danio_rerio_LeptinA            MRFP-ALRST CILSMLSLIH C---IPVHQH DRK---NVKL QAKTIIVRIR
Danio_rerio_LeptinB            MKSS-MIFCL LISSLVAVSI S---RP-TAP EDR----IRI IARTTISRIK
Ctenopharyngodon_idella_LepA   MYSP-VLLYT CFLSILGMID GRS-IPIHQ- DN-----LKN LVKLQADTII
Ctenopharyngodon_idella_LepB   MHSS-LIFSI LMAALVAVSI S---RP-TAT EDR----IRI MARTTISRIK
Carassius_auratus_LeptinI      MYFP-ALLYP CILGMLSLVH G---IPVHP- DR-----LKN MVKLQADTII
Carassius_auratus_LeptinII     MYFP-ALLYP CILGMLGLVH A---IPVHQ- DS-----LKN LVKPQAETII
Cyprinus_carpio_LeptinA1       MYFS-ALLYP CILAMLSLVH G---IPIHS- DS-----LKN LVKLQADTII
Cyprinus_carpio_LeptinA2       MYFS-VLLYP CILGMLSLVH A---IPVHP- DS-----LKN LVKLQADTII
Cyprinus_carpio_Jian_LeptinA   MYFS-ALLYP CILAMLSLVH G---IPIHS- DS-----LKN LVKLQADTII
Cyprinus_carpio_Jian_LeptinB   MKSS-MIF-- CYLITALVAV SIS-RPTATE ER-----IR- ---IMARTII
Hypophthalmichthys_molitrix_Le MYFP-VLLYT CFLSILGLID GRS-IPFHP- ES-----LKS L-KQQADTII
Schizopygopsis_pylzovi_Leptin  MYF-AALLYP CILSMLSLVH G---IPVHQ- NS-----LKN LVKMQADTII
Salmo_salar_LeptinA1           MDCSMALLLS SLLALFSVGA G---ASLSL- HV-----VRT KVKDLAQTMV
Salmo_salar_LeptinA2           MDCSMALLVS SLLALFSMGA G---ASLSL- DI-----VRT KVKFLAQTMV
Salmo_salar_LeptinB1           MDVSVVLLCL GLVVSVSVCH PQRGRPLNG- DV----QMRN NIKLLSMITV
Salmo_salar_LeptinB2           MHVSVVLLCL GLVVSVSVCH PQRGRPLNG- GV----QMRN NIKLLSMITV
Oncorhynchus_mykiss_LeptinA1   MDCSMALLLS SLLALFSVGA G---ASLSL- HV-----VRT KVKDLAQTMV
Oncorhynchus_mykiss_LeptinB1   MHVSVVLLCL GLVVSVSVCH PQRGRPLNG- DV----QMGN NIKLLAMITV
Salvelinus_alpinus_Leptin      MDCSMAPLVS FLLAHFSMGA G---ASLSL- DI-----VRT KVKFLAQTMV
Salvelinus_alpinus_LeptinA1    MDCSMALLLS SLLALFSVGA G---ASLSL- HV-----VRT KVKDLAQTMV
Salvelinus_alpinus_LeptinB1    MHVSVVLLCL GLVVSVSVCH PQRGRPLNG- DV----QMRN NIKLLAMITV
Oreochromis_mossambicus_Leptin MDYGLVLLFS LFQA-LSMGT A---APLPV- EVV---TMKS KVKWMAEQLV
Epinephelus_coioides_LeptinA   MDYTLALLFS LLHV-FSVGT A---APLPV- EVV---KMKS KVKWMAEQLV
Epinephelus_coioides_LeptinB   MHIFRALVYV SLVV-APGCS S---LPTKG- DS-----IRN TIHSIINIAQ
Larimichthys_crocea_Leptin     MDYSLALLFT LLHL-LSVGT A---APLSA- EVV---KMKS KVKWMAEQLV
Morone_saxatilis_Leptin        MDYTLAILFS MLQL-LSVSM A---APLPV- EVV---KMKS KVKWMAEQLV
Takifugu_rubripes_Leptin       MDHILALVLA LLPL--SLCV A---LPGAL- DAMDVEKMKS KVTWKAQGLV
Tetraodon_nigroviridis_Leptin  MDYTLALALS LLQL--SMCT P---VPMMQ- DSG---RMKT KAKWMVQQLL
Oryzias_latipes_LeptinA        MDSALVLFAF LFHC-LNVAT A---APVNP- EL---QEMKS NVIDIAKELS
Oryzias_latipes_LeptinB        MYMPLALVYA SFLTLPAST- ----SPATK- GNVI-QIQVH NIVNLAQTTV
Tachysurus_fulvidraco_Leptin   MAVYPALFCS CVVTVLTLTN GRA-LP--T- DS-----LKN SVKLQVENII
Anguilla_anguilla_Leptin1      MHHFIILHCT SLLFLLTMGK S---TPPPV- DK-----MKN NVKMLGETAL
Anguilla_anguilla_Leptin2      MSGCVALLCT SLLVLLPLGA G---VPLSV- ET-----MKS NVKLMAQTTI
Homo_sapiens_Leptin            MHWGTLCGFL WLWPYLFYVQ A---VP--I- QK-----VQD DTKTLIKTIV
Bos_taurus_Leptin              MRCGPLYRFL WLWPYLSYVE A---VP--I- CK-----VQD DTKTLIKTIV
Lepisosteus_oculatus_Leptin    MKYPVIPFCL SSWMFLTLSY S---RPLAE- DR-----VKN DARLLAQTTV

                              51
Danio_rerio_LeptinA            EHIDGQN--- --LLPTLIIG DPGH-YPEIP ADKPIQGLGS IMETINTFHK
Danio_rerio_LeptinB            KIKDEHFQ-- --MSPEIDFG -PDI------ -DNPIDGLSS VLSYLSYLQL
Ctenopharyngodon_idella_LepA   HRIKEHNEK- LKLSPKILIG DSEL-YPEVP ADKPIQGLGS IVDTLTTFQK
Ctenopharyngodon_idella_LepB   KIKDEHFQ-- --MSPEIDFG -PDI------ -DTPIDGLTS IYVHLSYLQL
Carassius_auratus_LeptinI      LRIKDHNEK- LKLYPKLLIG DPEL-YPEVP ADRHIQGLGS IMDTLTIFQK
Carassius_auratus_LeptinII     LRIKDHN-K- LKLSPKIFI- DPEL-YPEVP ADKPIQGLGS IVDTLTTFQK
Cyprinus_carpio_LeptinA1       IRIKDHNAE- LKLYPKLLIG DPEL-YPEVP ADKPIQGLGS IMDTITTFQK
Cyprinus_carpio_LeptinA2       LRIKDHNEK- LKLSPKLLIG DPEL-YPEVP ANKPIQGLGS IVETLSTFHK
Cyprinus_carpio_Jian_LeptinA   IRIKDHNAE- LKLYPKLLIG DPEL-YPEVP ADKPIQGLGS IMDTITTFQK
Cyprinus_carpio_Jian_LeptinB   TRIKKIKDEH FQMSPEIDFG -TDI------ -DTPTDGLTS VFVHLSYLQL
Hypophthalmichthys_molitrix_Le HRIKEHNEK- LKLSPKILIG DSEL-YPEVP ADKPIQGLGS IIDTLTTFQK
Schizopygopsis_pylzovi_Leptin  LRIKNHNEK- LKLSPKLVIG GPEL-YPEVP ADKPLQGLGS IVDTLTTFQK
Salmo_salar_LeptinA1           IRIN----K- LDISPNLIEG -MDPFLPAAA VDQHIESLPS IMETMGFYQD
Salmo_salar_LeptinA2           YRTQMGIKK- LPSSSNLVID GLELFFPAAA GDQPIGGLPS IVETMGFYQD
Salmo_salar_LeptinB1           VHIKNYLTE- FDVPPEMEFN ---------P MNPPIEGLAS IWVHLGGLEE
Salmo_salar_LeptinB2           VHIKNYLTE- FDVPPEMEFN ---------P MNPPIEGLAS IWVHLGGLEE
Oncorhynchus_mykiss_LeptinA1   IRIK----K- LDI-SPNLIE GMDPFLPAAA VDQHIESLPS IVETMGFYQD
Oncorhynchus_mykiss_LeptinB1   VHIKNYLTE- FNVPPEMEFN ---------P MNPPIEGLAS IWVHLGGLED
Salvelinus_alpinus_Leptin      YRTQMEIKK- LPSSSNLVID GLELFFPAAA GDQPIEGLPS IVETMGFYQD
Salvelinus_alpinus_LeptinA1    IRIK----K- LDI-SPNLIE GMDPFLPAAA VDQHIESLPS IVETMGFYQD
Salvelinus_alpinus_LeptinB1    VHIKNYLTE- FNVPPEMEFN ---------P MNPPIEGLAS IWVHLGGLED
Oreochromis_mossambicus_Leptin VRLDKDVQV- ---PVNWTLN ---------P PADDLDGTSS IVTVLNGYNS
Epinephelus_coioides_LeptinA   VRLNKDFQV- ---PPGLTLS ---------P PADILDGPSS IVTVLDGYNS
Epinephelus_coioides_LeptinB   ITLVHIKKLR TRLPAAPQIE ---------P STPSIDGLTS ITQDLGLLDN
Larimichthys_crocea_Leptin     LKLDRNFQV- ---PPGLTLS ---------P PVDVLDGPAS IVMVLEGYNS
Morone_saxatilis_Leptin        VRLNRDFEV- ---PAGLTLS ---------P PADDLDGLSS IVTILEGYNS
Takifugu_rubripes_Leptin       ARIDKHFP-- ---DRGLR-- ---------F DTDKVEGSTS VVASLESYNN
Tetraodon_nigroviridis_Leptin  VRLKDNVWP- -HFDMPPT-- ---------F SADDLEGSAS IVARLENFNS
Oryzias_latipes_LeptinA        LRLESIIQT- ---SIGPKFS ---------P PSDELNGLSS IMAVLDECTN
Oryzias_latipes_LeptinB        AHIRKLRMQ- -LLMAPPIE- ---------I TTPPIKGLAS FSHYLKHLDN
Tachysurus_fulvidraco_Leptin   SRIQKHKDE- FPILHKMILD SPEL-LPELQ SDKPIEGLSS MVEMLNNFQR
Anguilla_anguilla_Leptin1      IRIQKFTNE- FQISPNMVFS GAEL-IPNIT LETPL-GLSS VAENLNTFQL
Anguilla_anguilla_Leptin2      VRIQKLTEE- FRISPNMVFS GLEL-IPDIA PDKAWEGLSA IAQGLHSFQV
Homo_sapiens_Leptin            TRINDISHT- QSVSSKQKVT GLDF----IP GLHPILTLSK MDQTLAVYQQ
Bos_taurus_Leptin              TRINDISHT- QSVSSKQRVT GLDF----IP GLHPLLSLSK MDQTLAIYQQ
Lepisosteus_oculatus_Leptin    IRIQKHTNE- SKMSPNLVFS GLEL-IPDAL NDKTLEGLST VEDNLHTFQE
```

```
                                101
Danio_rerio_LeptinA             VLQKLPN-KH VDQIRRDLST LLGYLE---- GMDCTLK--- ESTNGKALDA
Danio_rerio_LeptinB             RLHVPPA-QH LQQVQIDLET LLRTLEELAV SQGCPLP--- NPET-----P
Ctenopharyngodon_idella_LepA    ILQTLPK-GH VSQLHNDMST LLEYFKDRMT FMRCTLK--- EPANGKSLDT
Ctenopharyngodon_idella_LepB    RLRVPPA-QH LQQVQVDLET LLSTLEGLAT SQGCTLP--- NPET-----P
Carassius_auratus_LeptinI       VLQRLPK-GH VSQIRSDLST LLGYLKERTT SMHCILK--- EPANGRSLDA
Carassius_auratus_LeptinII      ILQRLPN-GN VSQISTDLFT LLGNLKDRMK SLRCTLK--- EPANDRSLDA
Cyprinus_carpio_LeptinA1        VLQRLPK-GR VSQIHIDLST LLGHLKERMT SMHCTSK--- EPANGRALDA
Cyprinus_carpio_LeptinA2        VLQRLPK-GH VSQIRNDLFT LLGYLKDRMT SMRCTLK--- EPANERSLDA
Cyprinus_carpio_Jian_LeptinA    VLQRLPK-GR VSQIHIDLST LLGHLKERMT SMHCTSK--- EPANGRALDA
Cyprinus_carpio_Jian_LeptinB    RLRVPPA-LH LQQVQVDLET LLRTLEGLAV SQGCPLP--- NPET-----P
Hypophthalmichthys_molitrix_Le  VLQTLPK-GH VSQLHSDVST LLDYFKVWMT FMRCTPK--- EPANGKSLDT
Schizopygopsis_pylzovi_Leptin   VLQKLPK-GH VSQIYNDLST LVGYLE-RMK SMLCTLK--- EPSNDRSLDA
Salmo_salar_LeptinA1            LMLFLDW-AD LKQLVEDTST MRGLLENWMI S-RCPGRQQK QTGEGRLSEA
Salmo_salar_LeptinA2            LLIFLDM-AD LKQLVEDAST MRGQLENWMM S-RCPGRQQK QIGEGGLEEA
Salmo_salar_LeptinB1            SLQDSR---- CGQVYEDLSS MRGWVHSLSQ ALGCPDL--- -AKPGG--EA
Salmo_salar_LeptinB2            SLQDSR---- CGQVYEDLSS MRGWVHSLSQ ALGCPDL--- -AKPGG--EA
Oncorhynchus_mykiss_LeptinA1    LLLVLDW-AD LKQLVEDTST MRGLLENWMT S-RCPARQQK QTGEGGLSEA
Oncorhynchus_mykiss_LeptinB1    SLQDSR---- CGQVYEDLSS MRGWVHSLSQ ALDCPDL--- -AKPGGK--A
Salvelinus_alpinus_Leptin       LLISLDM-AD LKQLVEDAST MRGQLENWMM S-RCPGRQQK QTGEGGLEEA
Salvelinus_alpinus_LeptinA1     LLLFLDW-AD LKQLVEDTST MRGLLENWMI S-RCPGHQQK QTGEGGLSEA
Salvelinus_alpinus_LeptinB1     SLQDSR---- CGQVYEDLSS MRGWVHSLSQ ALDCPDL--- -AKPGG--EA
Oreochromis_mossambicus_Leptin  LIPDTF--KG VSQIKYDISS LTGYIHLWRQ G-HCSEQRPK PEVPG--PLQ
Epinephelus_coioides_LeptinA    LISDTF--NG VSQVKFDISS LTGYIGQWRQ G-HCTEQRPK PSVPG--PLQ
Epinephelus_coioides_LeptinB    ELQNPVT-EL LSQIQADVSS LEGRVRSFAL TMDCPLQ--- ARPSGATSDS
Larimichthys_crocea_Leptin      LISDTL--DG VTQVKTEISS LAGYLDQWRK G-HCTEQRPK PSVPG--PLQ
Morone_saxatilis_Leptin         LISDSL--IG VSQVKVDISS LTGFLSQWRQ G-HCSEQRPK LSVPG--VLQ
Takifugu_rubripes_Leptin        LISDRF--GG VSQIKTEISS LAGYLNHWRE G-NCQEQQPK V---------
Tetraodon_nigroviridis_Leptin   LISDNL--GD VLQIKAEISS LTGYLKNWRH N-NCKEQRPR TAVPG--LPQ
Oryzias_latipes_LeptinA         QISDNF--DE AKKIKVDISS LMDSMSEWSD K-HCGEQPST --------QA
Oryzias_latipes_LeptinB         ELQSPDT-DL LSQIQADVSS LDGKVQSLGL MMNCPFQPRP T-------AEV
Tachysurus_fulvidraco_Leptin    VLHSLPK-GH MSQLHSDVST LQHYLEDRMS SLQCTH---R ITGTEKNLED
Anguilla_anguilla_Leptin1       ILLNLTL-DG TLQIRSDIVG LLDIVHWLAA SSSCPMKKPA SDGH---LET
Anguilla_anguilla_Leptin2       VLSHLPPGDG MAQVHADVLS LHGVVRSLAA SLGCPLHKPA GDGR---LEA
Homo_sapiens_Leptin             ILTSMPS-RN VIQISNDLEN LRDLLHVLAF SKSCHLP--W ASG-LETLDS
Bos_taurus_Leptin               ILTSLPS-RN VVQISNDLEN LRDLLHLLAA SKSCPLP--- QVRALESLES
Lepisosteus_oculatus_Leptin     ILSSLPM-EE MDQILADIFN LRMIIKSLAT SVNCAPL--- KSSNMSHLES

                                151
Danio_rerio_LeptinA             FLEDSASYPF TLEYMTLNRL KQFMQKLIDN LDQLKIC--- ---------
Danio_rerio_LeptinB             VHKEETAFPV TSNYLHLLEL QRFLEKLCLN IDKLKYCKDT DVAETFIL
Ctenopharyngodon_idella_LepA    FIEKNATHHI TFGYMALDRL KQFMQKLIAN LDQLKSC--- ---------
Ctenopharyngodon_idella_LepB    VHKEEAAFPV TSNYLYLPEL QRYLEKLCLN MDKLKWCKDT DVAEMFLF
Carassius_auratus_LeptinI       FLEENATHHI TLGYLALDRL KQFMQKLIVN LDQLKSC--- ---------
Carassius_auratus_LeptinII      FLENNATHPI TLGFLALDRL KQFMQKLIIN LDHLKSC--- ---------
Cyprinus_carpio_LeptinA1        FLEDNATHHI TVRYLALDRL KQFMQKLIVN LDQLKSC--- ---------
Cyprinus_carpio_LeptinA2        FLENNATHHI TFGFLALDRL KQFMQKLIVN LDHLKSC--- ---------
Cyprinus_carpio_Jian_LeptinA    FLEDNATHHI TVRYLALDRL KQFMQKLIVN LDQLKSC--- ---------
Cyprinus_carpio_Jian_LeptinB    VHKDETAFPV TSNYLYLLEL QRYLEKLCLN MDKLKWCKDT DVAETFIL
Hypophthalmichthys_molitrix_Le  FIQKNATHHV TFGYMALDRL KQFMQKLIAN LDQVKSC--- ---------
Schizopygopsis_pylzovi_Leptin   FLEDSATHHI TFGHLALDRL KQFMQKLIVN PDHLKSC--- ---------
Salmo_salar_LeptinA1            LKDTTRKYGL SVGPVALNRL KGYLGRLLLN LDQLNYCY-- ---------
Salmo_salar_LeptinA2            LKDSVRKFGL SVSPVALNRL KGYLDRLPLN LDQLRYC--- ---------
Salmo_salar_LeptinB1            LKT------V Y---QSLVEG QRYMEKISLN LDKLKIC--- ---------
Salmo_salar_LeptinB2            LKT------V Y---QSLVEG QRYMEKISLN LDKLKIC--- ---------
Oncorhynchus_mykiss_LeptinA1    LKDTTRKYGL SVGPVALNRL KGYLGRLLLN LDQLNYCY-- ---------
Oncorhynchus_mykiss_LeptinB1    LKT------V Y---QSLVEG QRYMGKISLN LDKLKIC--- ---------
Salvelinus_alpinus_Leptin       LKDSVRKFGL SVCPVALNRL KGYLDRLLLN LDQLRYC--- ---------
Salvelinus_alpinus_LeptinA1     LKDTTRKYGL SVGPVALNRL KGYLGRLLLN LDQLNYCY-- ---------
Salvelinus_alpinus_LeptinB1     LKT------V Y---QSLVEG QRYMEKISLN LDKLKIC--- ---------
Oreochromis_mossambicus_Leptin  ELQSHKEFIQ TVGIEALMRV KEFLNLLLKN LDQLETC--- ---------
Epinephelus_coioides_LeptinA    ELQSRKEFIH TVSIEALMRV KEFLNLLLKN LDHLETC--- ---------
Epinephelus_coioides_LeptinB    ------VFPD SQLHLTLTKV QRYLEKFILH KDKLKVC--- ---------
Larimichthys_crocea_Leptin      ELQSRKEFIH TVSIEALMRV KELLNLLLKN LDQLQSC--- ---------
Morone_saxatilis_Leptin         ELQRRKKFIH TVSMEALMRV KEFLNLLLKN LNHLETC--- ---------
Takifugu_rubripes_Leptin        -WPRRNIFNH TVSLEALMRV REFLKLLQKN VDLLERC--- ---------
Tetraodon_nigroviridis_Leptin   EPQRRKDFIQ SVTIDALMSM KEFLNLLLQN LDHLEIC--- ---------
Oryzias_latipes_LeptinA         ENQTSRRFSI TESMQAVTRL KHFLLLLQNN SDQLEIC--- ---------
Oryzias_latipes_LeptinB         SRF---LFPD IHHYWTIAKV ENYLESLHLN RDKLKVC--- ---------
Tachysurus_fulvidraco_Leptin    FPKNHSMYYI TVRHVALDRL QKYIQRLNHN LEQLRTC--- ---------
Anguilla_anguilla_Leptin1       FLKTNMPFQL SIANIVLTRL QEFLNKLINN LDQLKKC--- ---------
Anguilla_anguilla_Leptin2       FLKTNSTFHV TIGNVALERL RRFLGKLVQN LDQLKSC--- ---------
Homo_sapiens_Leptin             LGGVLEASGY STEVVALSRL QGSLQDMLWQ LDLSPGC--- ---------
Bos_taurus_Leptin               LGVVLEASLY STEVVALSRL QGSLQDMLRQ LDLSPGC--- ---------
Lepisosteus_oculatus_Leptin     FLKTNAAFHV TIGNVALERL QKYLLKLIRN LDQLKNC--- ---------
```
