# Supplementary figures and images for: Duplicated Leptin Receptors in Two Species of Eel Bring New Insights into the Evolution of the Leptin System in Vertebrates
Source: PLoS One. 2015 May 6;10(5):e0126008. doi: 10.1371/journal.pone.0126008 (PMC4422726; doi:10.1371/journal.pone.0126008)

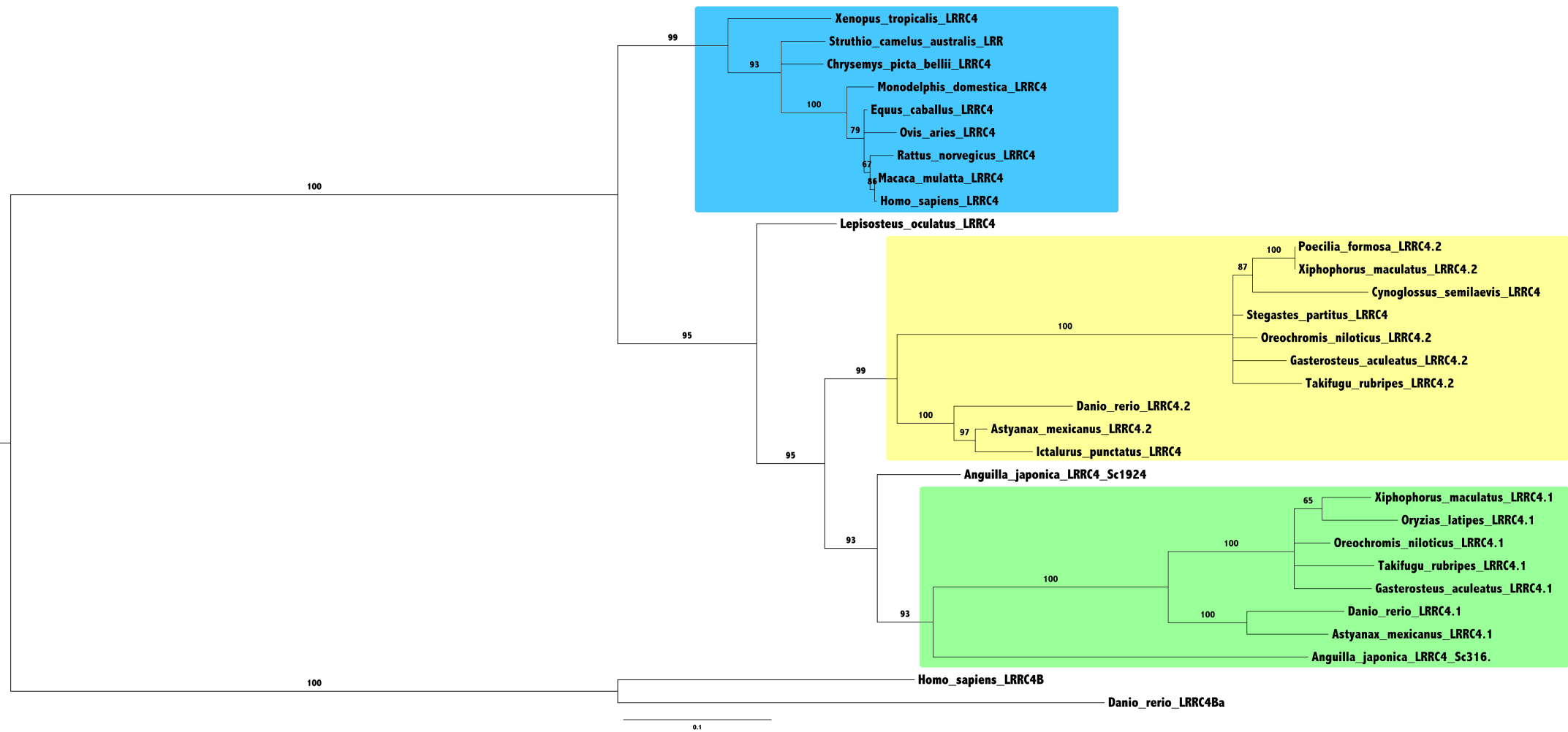

Supplement: S4 Fig — Phylogenetic analysis of 29 vertebrate LRRC4 amino acid sequences was performed using the Maximum Likelihood method, with 1,000 boostrap replicates (for the references of sequences, see S3 Table). The number shown at each branch node indicates in percentage the boostrap value. Only values above 50% are indicated. The tree was rooted using human and zebrafish LRRC4b sequences as outgroup. Sarcopterygian LRRC4 group is indicated in blue, teleost duplicated LRRC4 groups are indicated in green (type 1) and yellow (type 2). (PDF) [file pone.0126008.s004.pdf]

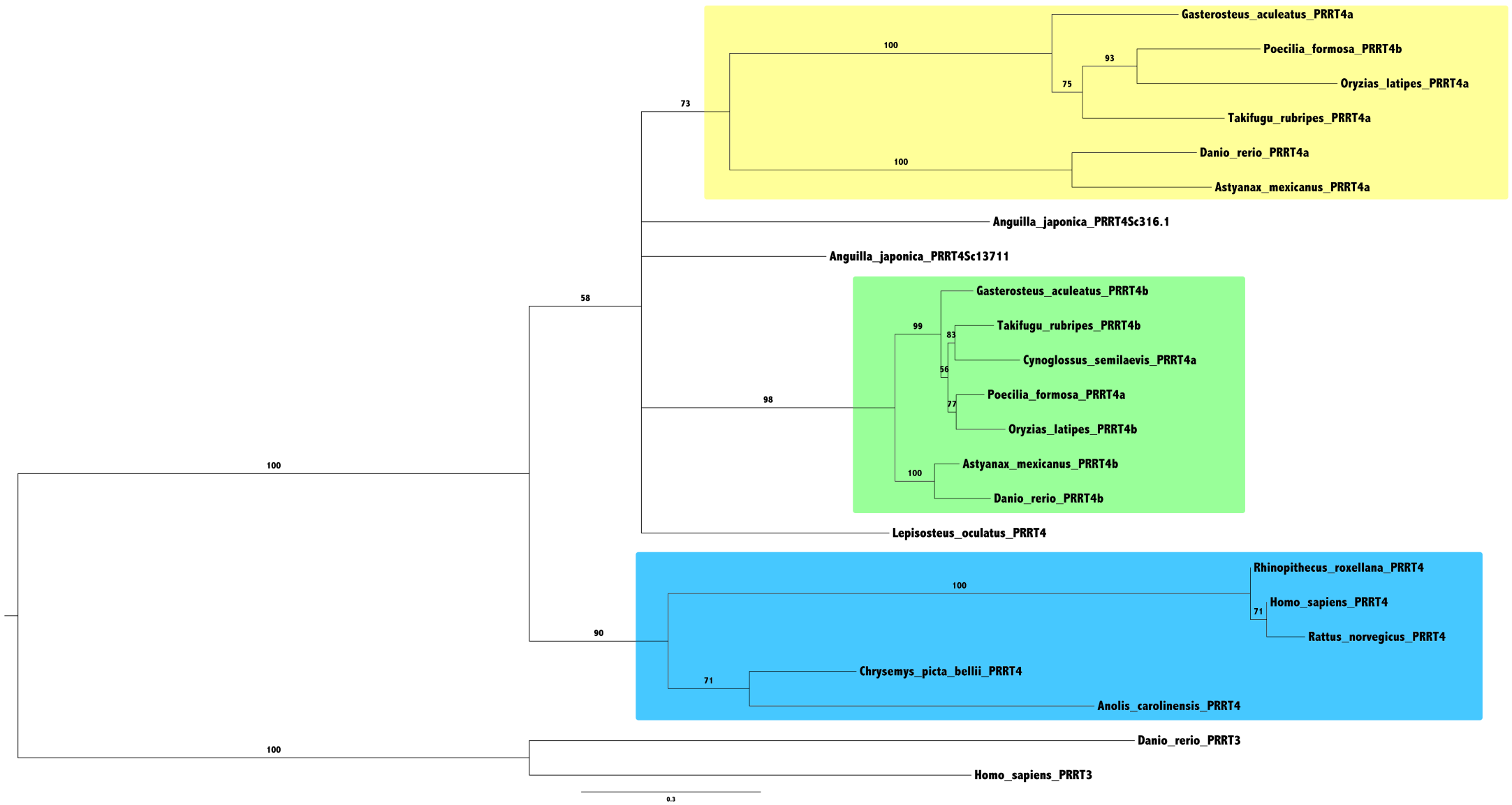

Supplement: S5 Fig — Phylogenetic analysis of 21 vertebrate PRRT4 amino acid sequences was performed using the Maximum Likelihood method, with 1,000 boostrap replicates (for the references of sequences, see S3 Table). The number shown at each branch node indicates in percentage the boostrap value. Only values above 50% are indicated. The tree was rooted using human and zebrafish PRRT3 sequences as outgroup. Sarcopterygian PRRT4 group is indicated in blue, teleost duplicated PRRT4 groups are indicated in yellow (“A” type) and green (“B” type). (PDF) [file pone.0126008.s005.pdf]

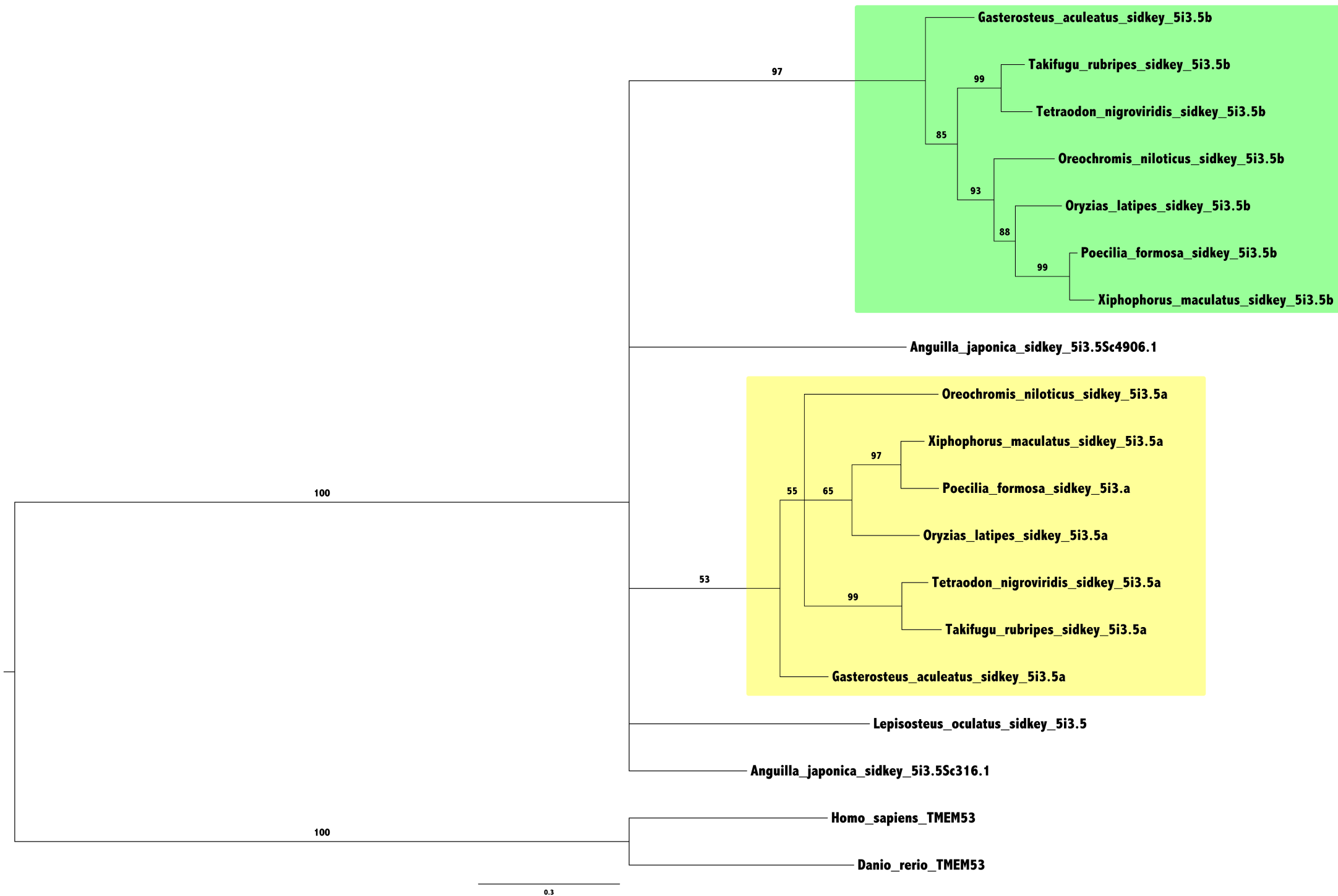

Supplement: S6 Fig — Phylogenetic analysis of 17 actinopterygian si:dkey-5i3.5 amino acid sequences was performed using the Maximum Likelihood method, with 1,000 boostrap replicates (for the references of sequences, see S3 Table). The number shown at each branch node indicates in percentage the boostrap value. Only values above 50% are indicated. The tree was rooted using human and zebrafish TMEM53 sequences as outgroup. Teleost duplicated si:dkey-5i3.5 groups are indicated in yellow (“A” type) and green (“B” type). (PDF) [file pone.0126008.s006.pdf]

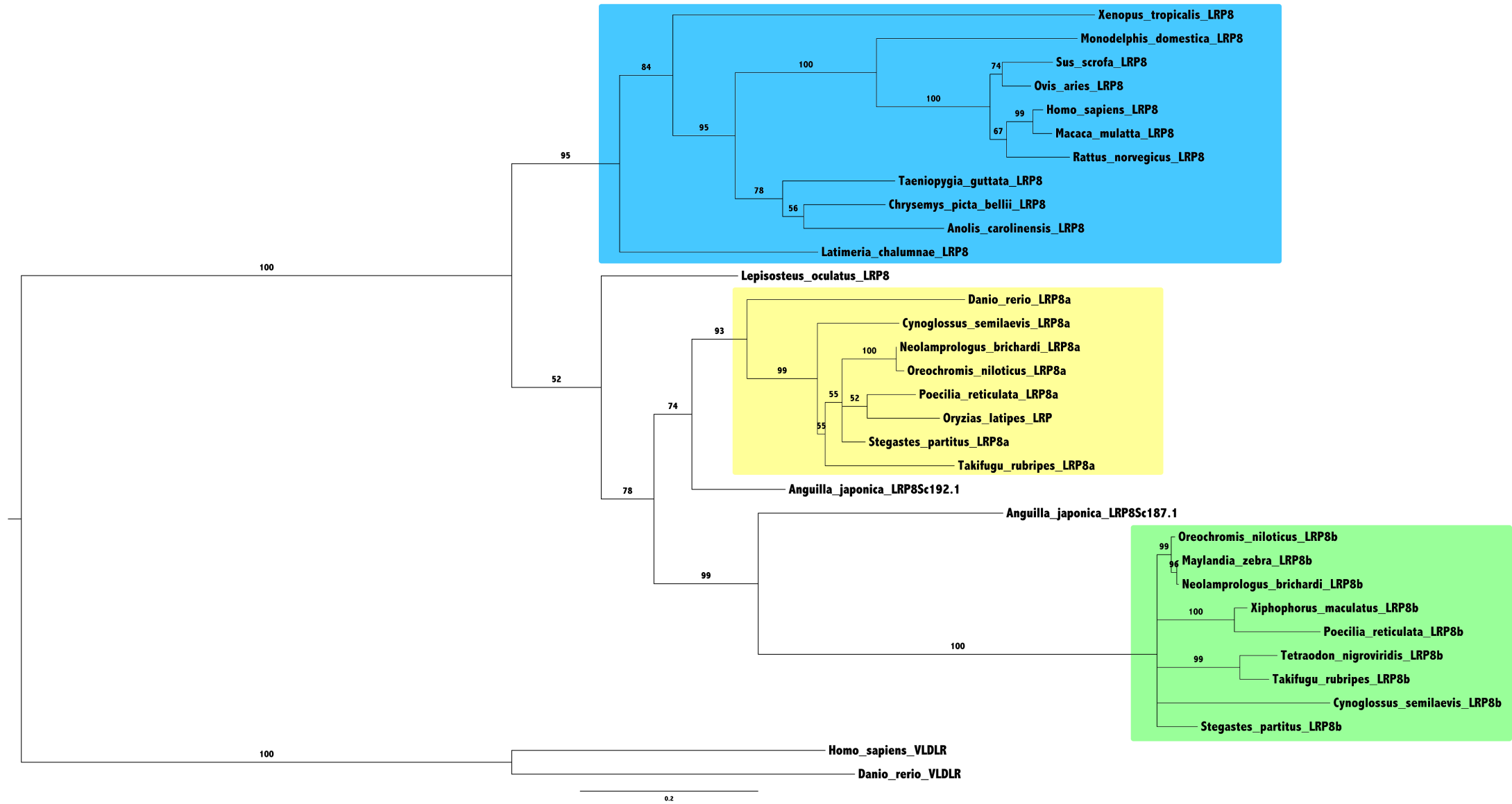

Supplement: S11 Fig — Phylogenetic analysis of 31 vertebrate LRP8 amino acid sequences was performed using the Maximum Likelihood method, with 1,000 boostrap replicates (for the references of sequences, see S3 Table). The number shown at each branch node indicates in percentage the boostrap value. Only values above 50% are indicated. The tree was rooted using human and zebrafish VLDLR sequences as outgroup. Sarcopterygian LRP8 group is indicated in blue, teleost duplicated LRP8 groups are indicated in yellow (“A” type) and green (“B” type). (PDF) [file pone.0126008.s011.pdf]

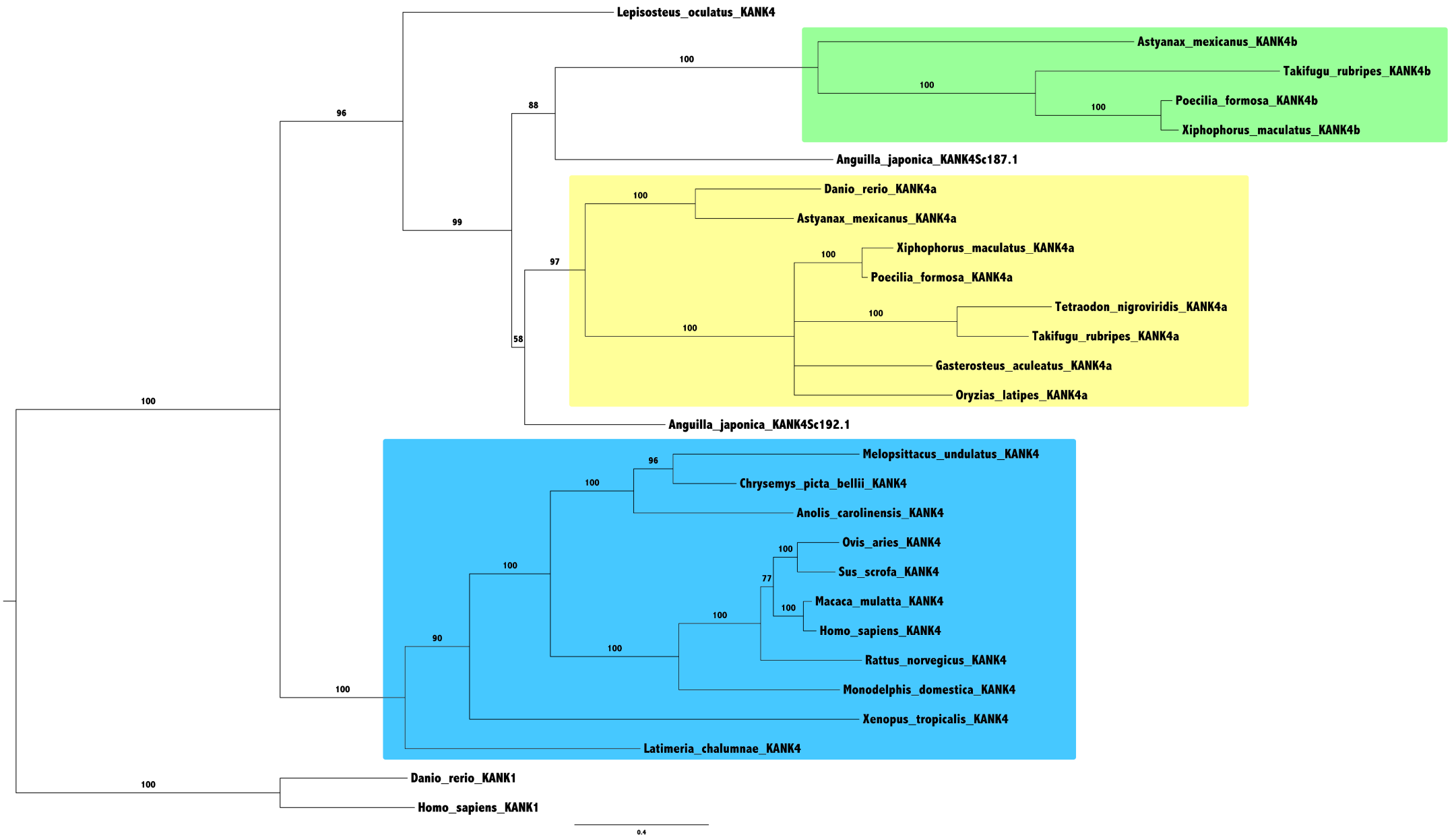

Supplement: S12 Fig — Phylogenetic analysis of 26 vertebrate KANK4 amino acid sequences was performed using the Maximum Likelihood method, with 1,000 boostrap replicates (for the references of sequences, see S3 Table). The number shown at each branch node indicates in percentage the boostrap value. Only values above 50% are indicated. The tree was rooted using human and zebrafish KANK1 sequences as outgroup. Sarcopterygian KANK4 group is indicated in blue, teleost duplicated KANK4 groups are indicated in yellow (“A” type) and green (“B” type). (PDF) [file pone.0126008.s012.pdf]

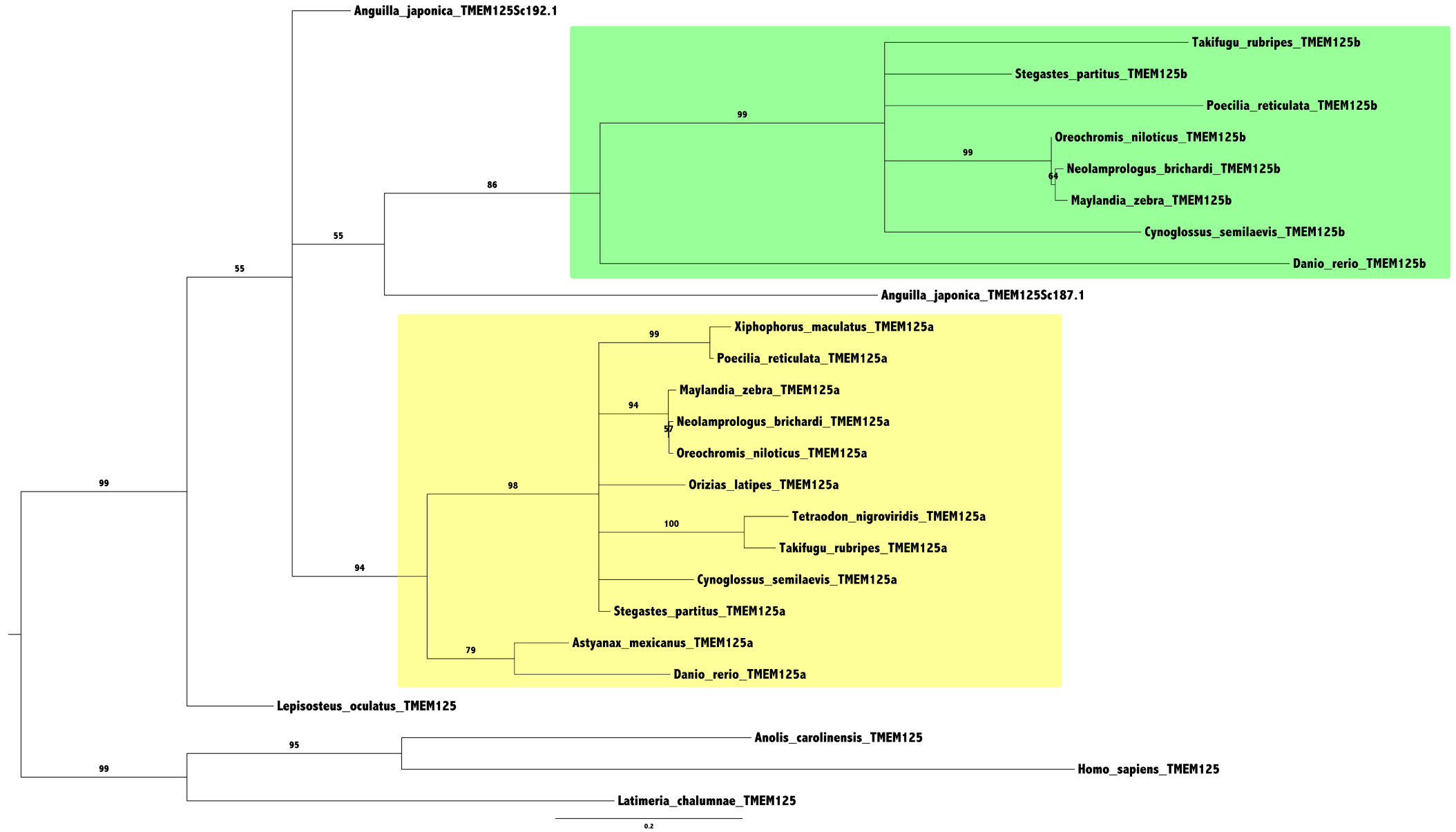

Supplement: S13 Fig — Phylogenetic analysis of 23 actinopterygian TMEM125 amino acid sequences was performed using the Maximum Likelihood method, with 1,000 boostrap replicates (for the references of sequences, see S3 Table). The number shown at each branch node indicates in percentage the boostrap value. Only values above 50% are indicated. The tree was rooted using sarcopterygian (human, lizard and coelacanth) TMEM125 sequences as outgroup. Teleost duplicated TMEM125 groups are indicated in yellow (“A” type) and green (“B” type). (PDF) [file pone.0126008.s013.pdf]

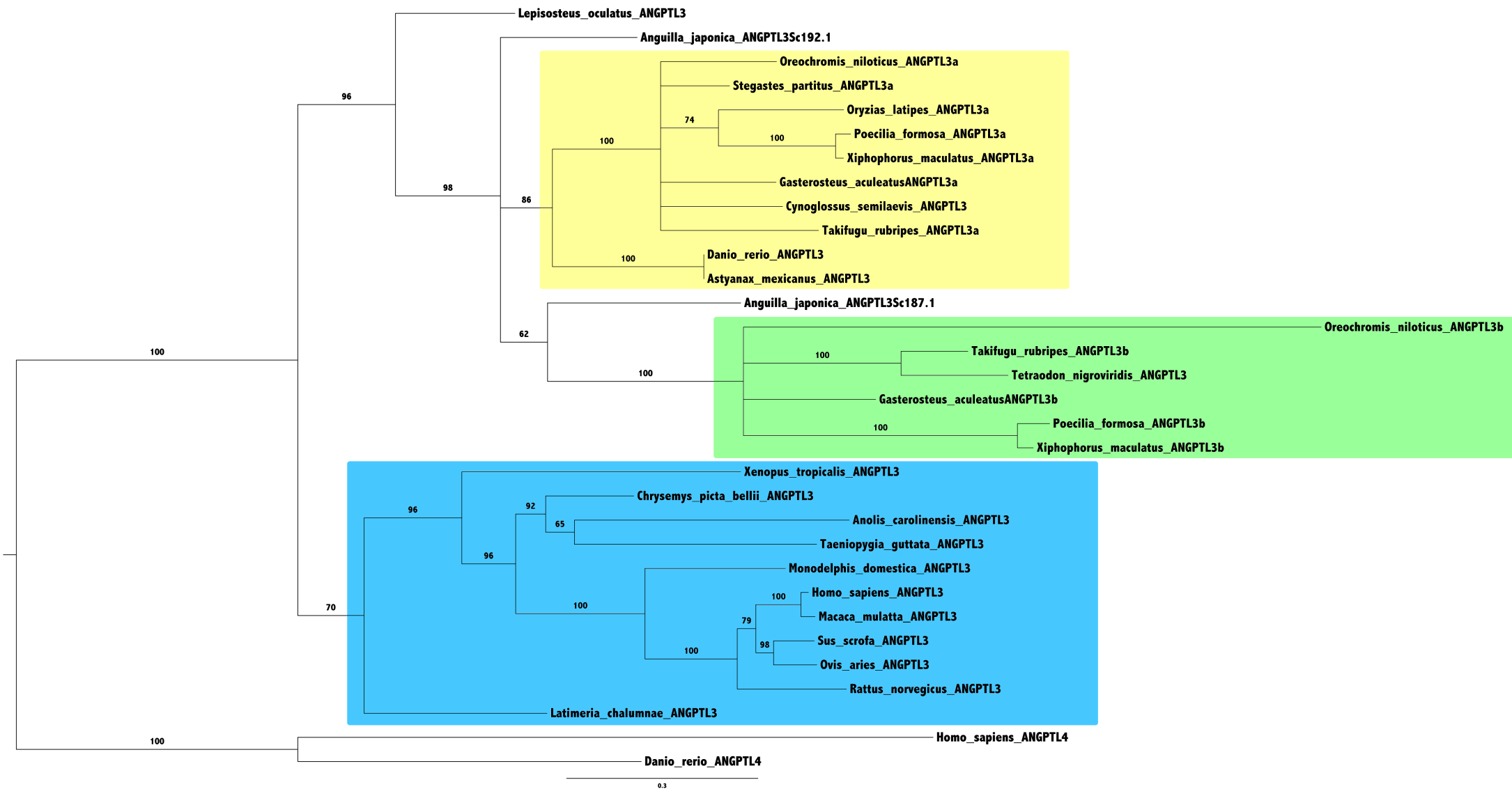

Supplement: S14 Fig — Phylogenetic analysis of 30 vertebrate ANGPTL3 amino acid sequences was performed using the Maximum Likelihood method, with 1,000 boostrap replicates (for the references of sequences, see S3 Table). The number shown at each branch node indicates in percentage the boostrap value. Only values above 50% are indicated. The tree was rooted using human and zebrafish ANGPTL4 sequences as outgroup. Sarcopterygian ANGPTL3 group is indicated in blue, teleost duplicated ANGPTL3 groups are indicated in yellow (“A” type) and green (“B” type). (PDF) [file pone.0126008.s014.pdf]

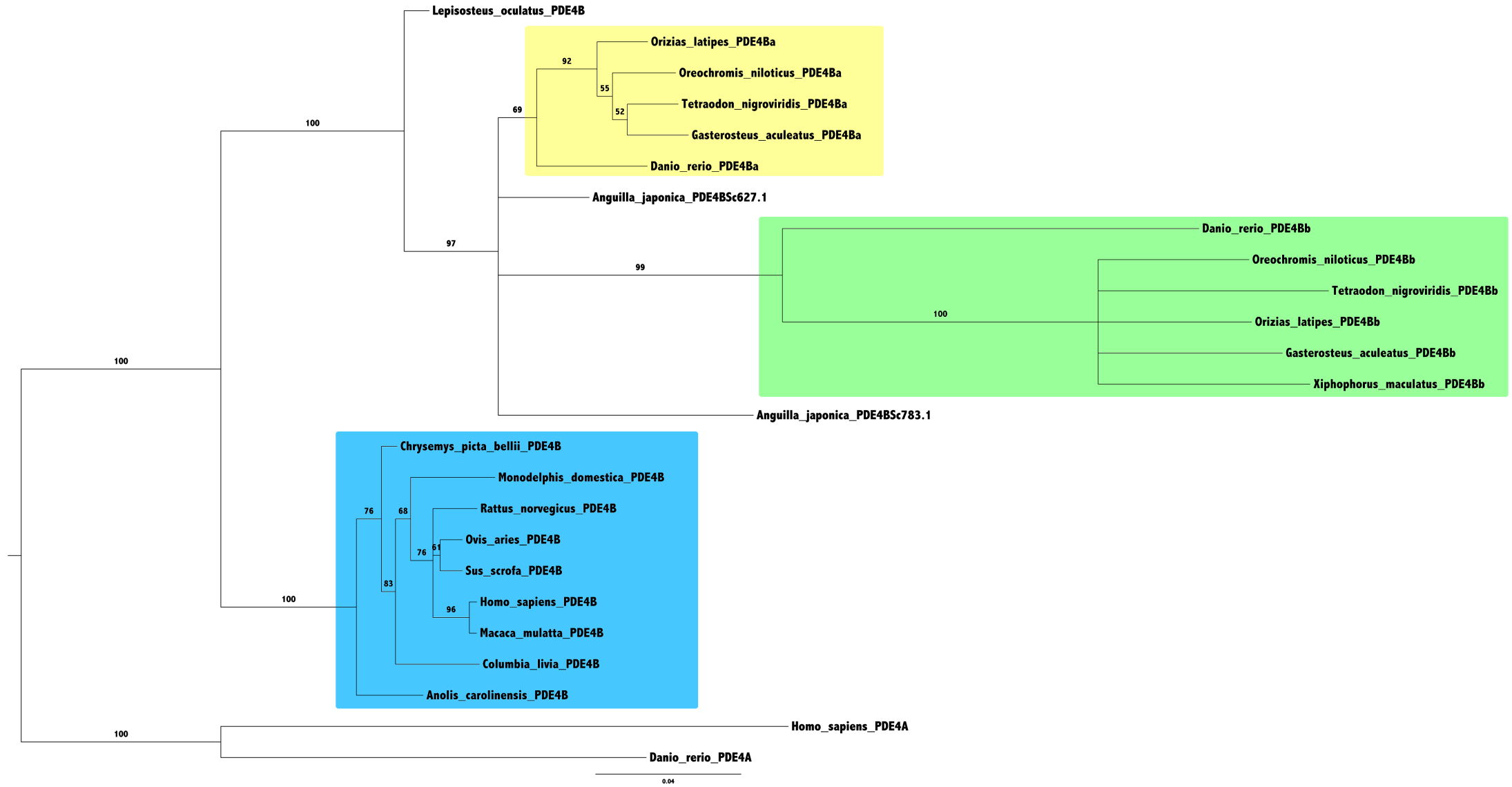

Supplement: S15 Fig — Phylogenetic analysis of 23 vertebrate PDE4B amino acid sequences was performed using the Maximum Likelihood method, with 1,000 boostrap replicates (for the references of sequences, see S3 Table). The number shown at each branch node indicates in percentage the boostrap value. Only values above 50% are indicated. The tree was rooted using human and zebrafish PDE4A sequences as outgroup. Sarcopterygian PDE4B group is indicated in blue, teleost duplicated PDE4B groups are indicated in yellow (“A” type) and green (“B” type). (PDF) [file pone.0126008.s015.pdf]

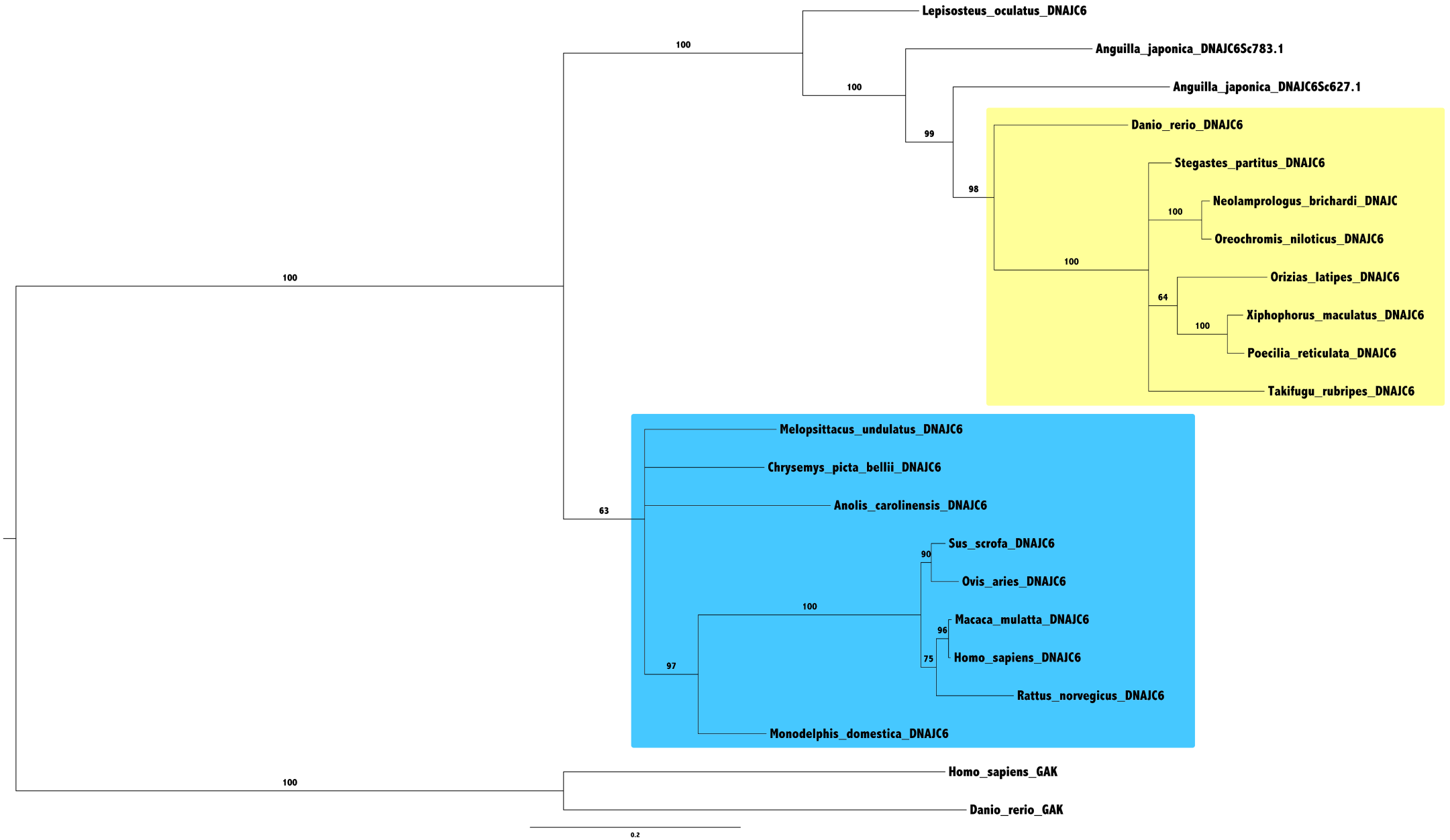

Supplement: S16 Fig — Phylogenetic analysis of 20 vertebrate DNAJC6 amino acid sequences was performed using the Maximum Likelihood method, with 1,000 boostrap replicates (for the references of sequences, see S3 Table). The number shown at each branch node indicates in percentage the boostrap value. Only values above 50% are indicated. The tree was rooted using human and zebrafish GAK sequences as outgroup. Sarcopterygian DNAJC6 group is indicated in blue, teleost DNAJC6 group is indicated in yellow. (PDF) [file pone.0126008.s016.pdf]

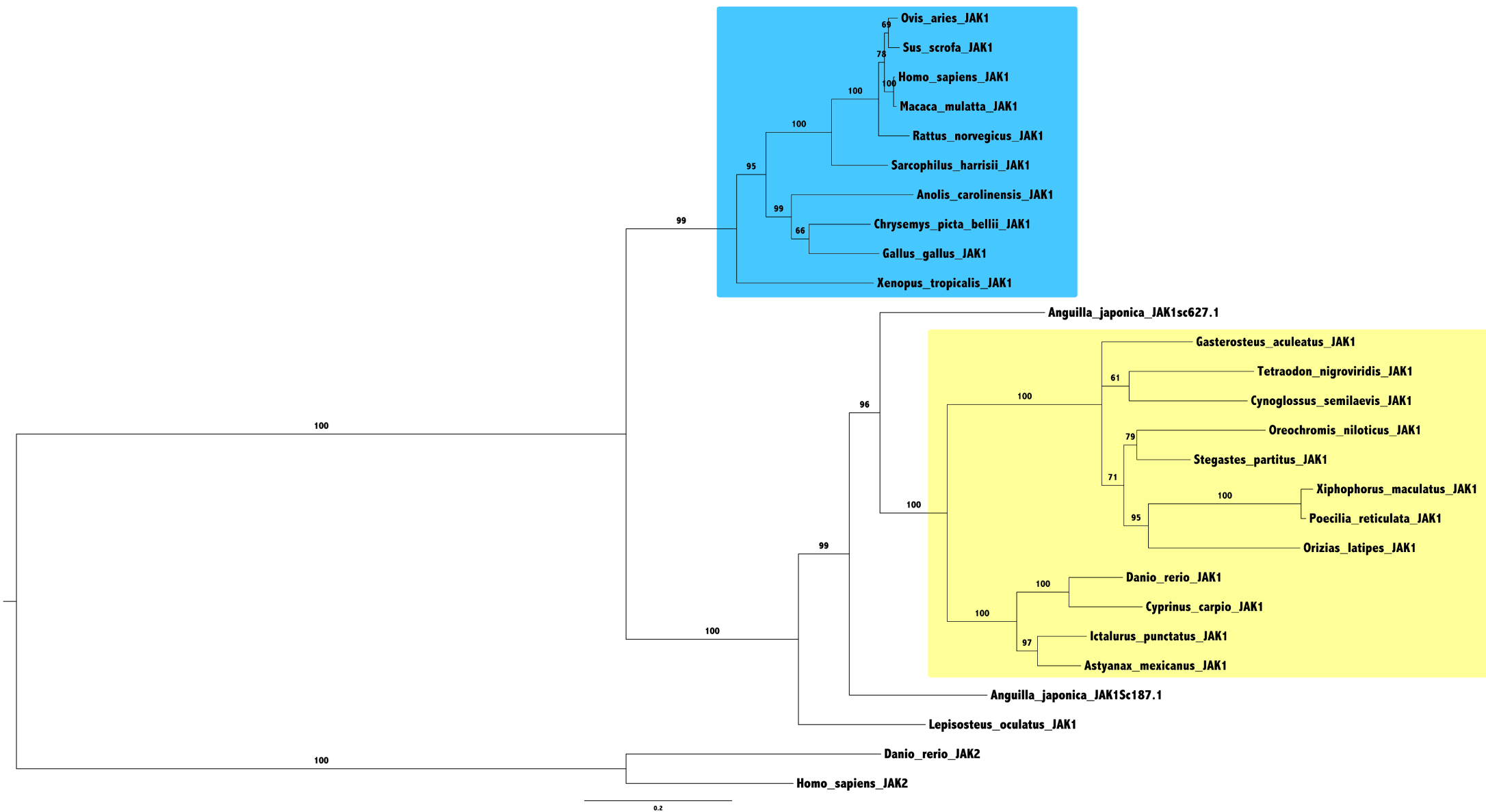

Supplement: S17 Fig — Phylogenetic analysis of 25 vertebrate JAK1 amino acid sequences was performed using the Maximum Likelihood method, with 1,000 boostrap replicates (for the references of sequences, see S3 Table). The number shown at each branch node indicates in percentage the boostrap value. Only values above 50% are indicated. The tree was rooted using human and zebrafish JAK2 sequences as outgroup. Sarcopterygian JAK1 group is indicated in blue, teleost JAK1 group is indicated in yellow. (PDF) [file pone.0126008.s017.pdf]
