# Supplementary material for: Duplicated Leptin Receptors in Two Species of Eel Bring New Insights into the Evolution of the Leptin System in Vertebrates
Source: PLoS One. 2015 May 6;10(5):e0126008. doi: 10.1371/journal.pone.0126008 (PMC4422726; doi:10.1371/journal.pone.0126008)
Supplement: S7 Fig — Signal peptide is shaded in black. Transmembrane domain is shaded in green. Ig-like C2-type domain is shaded in light blue. Fibronectin type 3 domains are shaded in red. WSXWS motifs are indicated in blue letters. The leptin binding site is boxed. JAK box are shaded in purple. STAT box is shaded in yellow. (PDF) [file pone.0126008.s007.pdf]

Fig S7A LEPRa

```
   1 - ATTCTAGTCGGGCAAACACGCGACGAAAGGAAGAAAAGATAACTATCCAGTGAAATCTACTATCCAGCTGAGGGCCA
  79 - TGCTGAATTACATATGAGATGTCTTTCTCTACAATGGGGATGTTTCTCCTGTGTCTTATCCTGGCTTTACACAGCGTG
   1 -                         M  S  F  S  T  M  G  M  F  L  L  C  L  I  L  A  L  H  S  V
 157 - GTGGGCGTGACGCTTTCAGATGGCAGGCTCCTGGGCACTCCGTGGGCAGCAGCGCTGTGCTGTGACCTGGCGGAACAG
  21 -  V  G  V  T  L  S  D  G  R  L  L  G  T  P  W  A  A  A  L  C  C  D  L  A  E  Q
 235 - GCACGCTCGCCCCGCTGCCACATAAGCCCCCACAGCAGCTCTCTGGCATCTCTCAATCATCTTCCCAGCAGAGGAGTT
  47 -  A  R  S  P  R  C  H  I  S  P  H  S  S  S  L  A  S  L  N  H  L  P  S  R  G  V
 313 - GTCTGTTTGAACATCCTGTGCTGGGTTGATGGGAACCTGGAAAATGTCATTTGTAATCTAAAGCTTTACGTCGGACTG
  73 -  V  C  L  N  I  L  C  W  V  D  G  N  L  E  N  V  I  C  N  L  K  L  Y  V  G  L
 391 - TCAGACACCAGCAGGCCCATTGCAATTAGCTTGCAGCGTTTGCCGTCACAGTTTGGTTCAGACAATGTGACAGACAGT
  99 -  S  D  T  S  R  P  I  A  I  S  L  Q  R  L  P  S  Q  F  G  S  D  N  V  T  D  S
 469 - GGGGCACACAGCAAATGTGAGGGACAAGATGTCCGGACATGTTCTGTTGCCCTCCATTCAGTCAACAGCACGGTGTCT
 125 -  G  A  H  S  K  C  E  G  Q  D  V  R  T  C  S  V  A  L  H  S  V  N  S  T  V  S
 547 - CTGATGATTTCCATTTCCGATGGTTCCAGCTCTGTGCAGTCACCAGTAATGGACATTGTCCCACTAAGTTTCTTGAAA
 151 -  L  M  I  S  I  S  D  G  S  S  S  V  Q  S  P  V  M  D  I  V  P  L  S  F  L  K
 625 - CCAGATCCCCCATTGAACCTGCAGTATCACATGACTATAGAAGGGGAGCTGAGATTGAGCTGGACCCATGCTCTACCC
 177 -  P  D  P  P  L  N  L  Q  Y  H  M  T  I  E  G  E  L  R  L  S  W  T  H  A  L  P
 703 - AGCGCTGAACGCTTTGTCTACGACATCCGCTACTCCTCCAGCTCCTTGCACTCCTGGATGCGCATGAGTGCAGAGGGT
 203 -  S  A  E  R  F  V  Y  D  I  R  Y  S  S  S  S  L  H  S  W  M  R  M  S  A  E  G
 781 - GCTCCTGGTGCCTCCCTTAAGGGGCTTAGTGTAGGGCTTAACTACACTGTCCAGGTGCGCTGCAAGACCCCAGGCAAC
 229 -  A  P  G  A  S  L  K  G  L  S  V  G  L  N  Y  T  V  Q  V  R  C  K  T  P  G  N
 859 - CAAGGCGTGTGGAGTGACTGGAGCCGCCCCCTTTATGTCTACCTAAACGAAGTGAGTTATATCCCTGAAAGATTATTC
 255 -  Q  G  V  W  S  D  W  S  R  P  L  Y  V  Y  L  N  E  V  S  Y  I  P  E  R  L  F
 937 - ACCAGCACGGGGTCCAACGTCACAATTTACTGCATTTTTAACAACCGAAGCCACAGTGCTAAAAATGCAGTTTGGTGG
 281 -  T  S  T  G  S  N  V  T  I  Y  C  I  F  N  N  R  S  H  S  A  K  N  A  V  W  W
1015 - CTGAGCTACCAGAAAGTTCCAGAGAGCCAGTACACCATTATCAGTGACCATGTGAGCAGTGTCACTTTGCTGAACGTG
 307 -  L  S  Y  Q  K  V  P  E  S  Q  Y  T  I  I  S  D  H  V  S  S  V  T  L  L  N  V
1093 - AAACCACTGAAACAACGAGGATATGACATCCTCCACTGCTGCCAACGAGAGGGAGAGAAGTCACTGTGCAGCTTCCCC
 333 -  K  P  L  K  Q  R  G  Y  D  I  L  H  C  C  Q  R  E  G  E  K  S  L  C  S  F  P
1171 - TATGCCCAGATTTACGTTAAGGATATCAGCGTTGCCATATCCTGTGAAGTCGATGGCGATCTCACAGCCATGACCTGC
 359 -  Y  A  Q  I  Y  V  K  D  I  S  V  A  I  S  C  E  V  D  G  D  L  T  A  M  T  C
1249 - ACCTGGAATACCAGCCAATGGGCTGAGGTCAGGTGTCTCTATCAAAGGCATATGCTTCCCTGTGAAGAGGAGATGGAA
 385 -  T  W  N  T  S  Q  W  A  E  V  R  C  L  Y  Q  R  H  M  L  P  C  E  E  E  M  E
1327 - TTGCCTCCTGCTGTGGAGTGTCCTGTGTTGGGGGCAAAGAGTTGTAGACTTGAGCCCCTATTCCTGACCTCTTGCTAC
 411 -  L  P  P  A  V  E  C  P  V  L  G  A  K  S  C  R  L  E  P  L  F  L  T  S  C  Y
1405 - ATAATGTGGGTAGAAGGAAGGGAGCAGGAGGGCACGGTCAAGTCCCACCCTATCTATATCCTGCCCATGGACTTGGTG
 437 -  I  M  W  V  E  G  R  E  Q  E  G  T  V  K  S  H  P  I  Y  I  L  P  M  D  L  V
1483 - AAGCCCCATCCTCCATTTGATCTGGGAGCCACCTTTCTGCCGGATGCTCATCTAAGTATCTGGTGGAAGAGGCCTGAA
 463 -  K  P  H  P  P  F  D  L  G  A  T  F  L  P  D  A  H  L  S  I  W  W  K  R  P  E
1561 - CTGCCTGTCTATGAGCTCCAGTTTGAGGTCCGGTACTCAGTGGACAAAGATGACACACTGCAAAAGGTAATCCGGTCC
 489 -  L  P  V  Y  E  L  Q  F  E  V  R  Y  S  V  D  K  D  D  T  L  Q  K  V  I  R  S
1639 - GTGTCCAATCAGTCAGCTGTGGTCCCGATTGTGGATCCGTGTGTGGTGTACACCATTCAGGTACGCTGCAAGCGGCTT
 515 -  V  S  N  Q  S  A  V  V  P  I  V  D  P  C  V  V  Y  T  I  Q  V  R  C  K  R  L
1717 - GCTGGACCTGGATTCTGGAGTGATTGGAGCAGCCCCTACTATACCACCATTAATAATATCAAAGCACCTGAACAGGGA
 541 -  A  G  P  G  F  W  S  D  W  S  S  P  Y  Y  T  T  I  N  N  I  K  A  P  E  Q  G
1795 - CCTGATTTCTGGAGAGTACTTCAGGAATATCCCAAACTGAATCAGACACATGTCACTCTTCTCTTCACGCTTTCGCAG
 567 -  P  D  F  W  R  V  L  Q  E  Y  P  K  L  N  Q  T  H  V  T  L  L  F  T  L  S  Q
1873 - AGCGAGGGCCCCTTCTGTTGCGTGGAGGGACTTACAGTTAAGCACCAGACCTCCAGGGGCTCTGTTTGGTCGGAGAAT
 593 -  S  E  G  P  F  C  C  V  E  G  L  T  V  K  H  Q  T  S  R  G  S  V  W  S  E  N
1951 - TTAGGCCGTGTCTCCACCTACTCCTTTTCCTGGACTGAAGATGTCCACACTGTCACTGTCCTGGCCCACAATGCCCTT
 619 -  L  G  R  V  S  T  Y  S  F  S  W  T  E  D  V  H  T  V  T  V  L  A  H  N  A  L
2029 - GGATCTTCAACCAAGAACAGCAACATGACACTGACCAGACACACCAAATCGCAATCCGTCCACTCGTTTAGCTCAATG
 645 -  G  S  S  T  K  N  S  N  M  T  L  T  R  H  T  K  S  Q  S  V  H  S  F  S  S  M
2107 - ATGGTAAACAGCAGCTGTGTGGCACTCTCCTGGACCCTGTTCCCAAACAGCTCTGCTCCGTCCTCATTTGTAATCCAG
 671 -  M  V  N  S  S  C  V  A  L  S  W  T  L  F  P  N  S  S  A  P  S  S  F  V  I  Q
2185 - TGGAGCGGCCAAAGCAGGAGCAGGCAGCAGGACAAGCAGGGGGGGAGAGTGAAGTGGGTGCGGGTTCCCCCCAACAAC
 697 -  W  S  G  Q  S  R  S  R  Q  Q  D  K  Q  G  G  R  V  K  W  V  R  V  P  P  N  N
2263 - CGTGCCTTTCACCTACATGAAACCTTCTTTGCCTCAGAGGAATACCAGTTTATTTTGTATCCAATATTTGAGAATACC
 723 -  R  A  F  H  L  H  E  T  F  F  A  S  E  E  Y  Q  F  I  L  Y  P  I  F  E  N  T
```

```
2341 — GAAGGAGAGCCCATCTACGCCAAAGACAGGGGGCGCCCCAGGGGAGACCATGCGGCCTACATGCTCCTGCTGATTATC
 749 -  E  G  E  P  I  Y  A  K  D  R  G  R  P  R  G  D  H  A  A  Y  M  L  L  L  I  I
2419 — ACCTTCCTGTCAGTGGTCTTGTTTGTGACACTCGCCGTCTCCCAGAATCAAATGAGGAAGCTGGTGTGGAAAGATGTT
 775 -  T  F  L  S  V  V  L  F  V  T  L  A  V  S  Q  N  Q  M  R  K  L  V  W  K  D  V
2497 — CCCAACCCCAACAACTGCTCCTGGGCCCAGGGGGTGGATTTTAGGAAGGCAGAGACCATCGAAAGCCTTTTCCGACAC
 801 -  P  N  P  N  N  C  S  W  A  Q  G  V  D  F  R  K  A  E  T  I  E  S  L  F  R  H
2575 — CCTGAGCGACTGATATCCTGCCCTCTGCTCCTGGAGTCGGAGACTATATCTGAGGCTGTAATTGTGGAAAAGACAAGC
 827 -  P  E  R  L  I  S  C  P  L  L  L  E  S  E  T  I  S  E  A  V  I  V  E  K  T  S
2653 — CCTGCGGTCCAAAACAATGAGAGGGATCAAGTCTTGGGCAAAGCCGGACAGGGGGAGATGGCCTCCTCTCTGCCCCCC
 853 -  P  A  V  Q  N  N  E  R  D  Q  V  L  G  K  A  G  Q  G  E  M  A  S  S  L  P  P
2731 — TGTGGAAACTCTGAGGAGCCCCCGGTCCAGGAGACATCCCTTTCCCCCGGTTCCCCGGATGACTCAGCCCAGTCCAGA
 879 -  C  G  N  S  E  E  P  P  V  Q  E  T  S  L  S  P  G  S  P  D  D  S  A  Q  S  R
2809 — ATCAGCTACGCCATGGTTCTGTTCCCAGGGACACCTGGCCTCCTGTACAAGCAGCAGGAGAGCCTGAGCAGCTCCTCT
 905 -  I  S  Y  A  M  V  L  F  P  G  T  P  G  L  L  Y  K  Q  Q  E  S  L  S  S  S  S
2887 — GATGAGGGAAACTTCTCCGCCAATAACTCTGATATCTCTGGCTCCTTTCCTGGGGGCTTGTGGGAGCTGGAGAATCCC
 931 -  D  E  G  N  F  S  A  N  N  S  D  I  S  G  S  F  P  G  G  L  W  E  L  E  N  P
2965 — CCGTCCAATGACTCCGATCCCCGACACTCCTGCTGCTACAACTCTGCTGAGGAGTTCTCTGATACTTCAGATCAGGAG
 957 -  P  S  N  D  S  D  P  R  H  S  C  C  Y  N  S  A  E  E  F  S  D  T  S  D  Q  E
3043 — GACGAAGTCTTGGATGGAACAGGTGCAGGAAAAGAACTCTACTACCTGGGCATGACCTCGCAAAGTGAAGAAGAAGAA
 983 -  D  E  V  L  D  G  T  G  A  G  K  E  L  Y  Y  L  G  M  T  S  Q  S  E  E  E  E
3121 — GATGAAAAGCAAGGTGAGGATGAAGAGGAGGAGGAGGAGGTAGGGGCGAGGTACCCAGGAGAGGATGCTCAAGAGCTC
1009 -  D  E  K  Q  G  E  D  E  E  E  E  E  E  V  G  A  R  Y  P  G  E  D  A  Q  E  L
3199 — CCTCTGGAGTCCAGCCCCCTGCTTGGTCGTTGGGATCCCAGATTGGACCGGACTGACGCAGCAGCTAAGGGCCCTCCA
1035 -  P  L  E  S  S  P  L  L  G  R  W  D  P  R  L  D  R  T  D  A  A  A  K  G  P  P
3277 — CTGTACGTGCCACAGTTCCGAACTGCGTCCAGGAAGTCCCAGCAGGCCAAAGCAAAGGAGCGGAGTGGAGTGGAGTCC
1061 -  L  Y  V  P  Q  F  R  T  A  S  R  K  S  Q  Q  A  K  A  K  E  R  S  G  V  E  S
3355 — CTCCAGGTCTGAAAGGTTCTGAACTGGGTCCGAGGCGCTGAGTTGGTCTTACTGTGGCTACAGAGGCCACATGTCGGA
1087 -  L  Q  V  *
3433 — TCTCCATTCTGAACTGTGTCTCACTGTCTGACTGAAGGGCTGCACAAGCCTGTCATTGTGCATCTTTCAGATAAAGGA
3511 — ATGTAGCCCATGTGTGTTAAGAGGAATTCTGACACCCATATGGCACTTTATGCTGTGTTGAAAGGTAAGCAAAGCTTA
3589 — AACAGGGGTGTGGGTAAGTGCTTTTAGTAGACTGATCTGTAAAAGACAAGCAGATGTTGGGTAATGTTACAAAGGCTG
3667 - ATTTCTGCTGCAGTCCACACACTCCTGTTGATTAA
```

Fig S7B LEPRb

```
   1 — GCACACAGTGGAGATGTCTGCGTTTCTCCCGCCCCACTCAGAAACGCTGGAGGTAGAACAGAGAAAGGAACTGTGCCC
  79 — GTGGACGAGGACTTCCCAGATGTGATGGAACTGCAGTTTAATCCTGTCACACTGTAAAACCTGCTTCGGTTCTCCCAC
 157 — TGGCTGAAGCTGTATATGCACTGGCTTAACTCCTTCTATTCTGGACCCTTTTCTGCAAAAGGAATGTTTGCAGTCTCA
   1 -                 M  H  W  L  N  S  F  Y  S  G  P  F  S  A  K  G  M  F  A  V  S
 235 — TTTCTCCTCACTTTGCATGGTGTGGTGGCTGTGCACTCTGCCGTTGCTCCTCCCCCTGAGTTTGAATACCCAGGAAAG
  22 -  F  L  L  T  L  H  G  V  V  A  V  H  S  A  V  A  P  P  P  E  F  E  Y  P  G  K
 313 — CTGGGGCTGTGCTGTTACCTAGGCGATGGATGGGCAGAGGAGACCGGAGGGGGCAACGGTGAGGGAGACAACAGCCGG
  48 -  L  G  L  C  C  Y  L  G  D  G  W  A  E  E  T  G  G  G  N  G  E  G  D  N  S  R
 391 — GATGGTATGGGGGGTGGGTCCCTTGGGCACACACCCCCAGGCCGGTGCAGCGTCTCCTCCCCCGCCCGCTTCCGCCAG
  74 -  D  G  M  G  G  G  S  L  G  H  T  P  P  G  R  C  S  V  S  S  P  A  R  F  R  Q
 469 — TCAGCCCTGACCTCCCAAAACCACAGCGTTGTGTGTCTGGATGTCTTATGCTGGGTTACCGGGGACAGGCGGCACCTA
 100 -  S  A  L  T  S  Q  N  H  S  V  V  C  L  D  V  L  C  W  V  T  G  D  R  R  H  L
 547 — GTTTGCGAGCGAAATCCGCGCGGCGGGAATGTGGCCGTGGGCGGGGTCGTGACCCTGAGCTTGCTGCGTTTACAATCA
 126 -  V  C  E  R  N  P  R  G  G  N  V  A  V  G  G  V  V  T  L  S  L  L  R  L  Q  S
 625 — GATCAGCAAATGCAGGCCACACCTTCCGCACTGCCAGACGGGGTCACACACAGTTGCCAGGAAGAGGGTGAGGGTGTG
 152 -  D  Q  Q  M  Q  A  T  P  S  A  L  P  D  G  V  T  H  S  C  Q  E  E  G  E  G  V
 703 — GGTGTATTTTGGTGTGCTCTTCCTCCTGGCTCGATGCGTGACGCAGTGACCCTGAGGGTGAATGTCGCCCGTGGTAAC
 178 -  G  V  F  W  C  A  L  P  P  G  S  M  R  D  A  V  T  L  R  V  N  V  A  R  G  N
 781 — CGCTCAGCGCTCTCTCCAGAGATTTCCTTTGTCCCGCAGAAGCTTGTGAGACCAGACCCCCCGGTAAAGCTGTGGTAC
 204 -  R  S  A  L  S  P  E  I  S  F  V  P  Q  K  L  V  R  P  D  P  P  V  K  L  W  Y
 859 — AACATGACCACTGAGGGAGAGCTGAGGCTGCACTGGACTCCCCCGCAACCCGTCACCGGCCCTCTGACCTACGATGTT
 230 -  N  M  T  T  E  G  E  L  R  L  H  W  T  P  P  Q  P  V  T  G  P  L  T  Y  D  V
 937 — CGTTACTCCTCCAACACCTCCCTCAACAGCTGGGTGCATGTGAATAAGGTGATAACTCAGCCCGTGACTCTGACGGGC
 256 -  R  Y  S  S  N  T  S  L  N  S  W  V  H  V  N  K  V  I  T  Q  P  V  T  L  T  G
1015 — ATGAATGCAGGCGTGACCTACACCGTTCAGGTGCGCTGTAAGATCCTGGGCAAGCCAGGCCTGTGGAGCGAGTGGAGC
 282 -  M  N  A  G  V  T  Y  T  V  Q  V  R  C  K  I  L  G  K  P  G  L  W  S  E  W  S
1093 — CAGTCCCTCTTCATATACCTGCATGAAGTCACATATCTCCCCAAATCAGTATTCACCAGCGAGGGAGCCAATGTGACC
 308 -  Q  S  L  F  I  Y  L  H  E  V  T  Y  L  P  K  S  V  F  T  S  E  G  A  N  V  T
1171 — GTATACTGCATATTCAATAACCAGAGCCTCAGTGCCAGGAATGTGGTTTGGTGGCTGAATATCCAAGAGAAGGTTCCA
 334 -  V  Y  C  I  F  N  N  Q  S  L  S  A  R  N  V  V  W  W  L  N  I  Q  E  K  V  P
1249 — GAAAGTCTGTACACCATCGTCAATGACCGCGTGAGCCGCGTCACCGTGCCGAACGTGAGGCCGCTCAAACGGCAGCAG
 360 -  E  S  L  Y  T  I  V  N  D  R  V  S  R  V  T  V  P  N  V  R  P  L  K  R  Q  Q
1327 — TACAACGTCCTCCAGTGCTGCCAGCGGAGCGGAGAGACGTCCCTCTGCAGCTACCGCTATGCTTCTCTCTACACTGAC
 386 -  Y  N  V  L  Q  C  C  Q  R  S  G  E  T  S  L  C  S  Y  R  Y  A  S  L  Y  T  D
1405 - GGTGTCAGTGTCGCCATATCCTGTGAGACCAATGGGACCTCAGTGCCATGACCTGCAGGTGGAACATTAGCCTGGGG
 412 -  G  V  S  V  A  I  S  C  E  T  N  G  D  L  S  A  M  T  C  R  W  N  I  S  L  G
1483 — GTCAGGTTCTATTACAGAACCAGTGATGTGCCTTTTGACATTGCAGAGGAGCAAATGGCTGTGTCCAAAGAAGAGGAG
 438 -  V  R  F  Y  Y  R  T  S  D  V  P  F  D  I  A  E  E  Q  M  A  V  S  K  E  E  E
1561 — TGTCCTTCTGAGGGGCGGGGCTTAAAGAGTTGCACCTTCCAGCCCTTTCTCCCATTCTCTTACTACATGATGTGGCTG
 464 -  C  P  S  E  G  R  G  L  K  S  C  T  F  Q  P  F  L  P  F  S  Y  Y  M  M  W  L
1639 — GAGTTTGGGACTGAAGAGGGGACAGTTAAGTCCCAGCCTGTGTATGCCTTGCCCATGGATTTGGTTAAGCCATACCCC
 490 -  E  F  G  T  E  E  G  T  V  K  S  Q  P  V  Y  A  L  P  M  D  L  V  K  P  Y  P
1717 — CCATTTGACCTGGAGGCTGTCACTGTGCCTGAGGGGTACCTGAGAGCCACATGGAAGCGGCCTGAACTACCCACCTAT
 516 -  P  F  D  L  E  A  V  T  V  P  E  G  Y  L  R  A  T  W  K  R  P  E  L  P  T  Y
1795 — GATCTCCTGTTTGAAGTGCGCTATGCGGTGGACGGGCCCGACCCACTGTGGAGGGTTTACAAATCTGAAGTAAATCTG
 542 -  D  L  L  F  E  V  R  Y  A  V  D  G  P  D  P  L  W  R  V  Y  K  S  E  V  N  L
1873 — ACGGTTGTTTTTCCTGTGTCGGACCCTTGTGCAGTTTACACTATTATGGTGCGTTGCAAACGCCTCCATGGGTCAGGG
 568 -  T  V  V  F  P  V  S  D  P  C  A  V  Y  T  I  M  V  R  C  K  R  L  H  G  S  G
1951 — TTCTGGAGTGAATGGAGTGATCCACATTACTCTGCTGTCCAGATCTCCAGAGCTCCTGAACGGGGACCCGATTTCTGG
 594 -  F  W  S  E  W  S  D  P  H  Y  S  A  V  Q  I  S  R  A  P  E  R  G  P  D  F  W
2029 — AGGGTTCTAAAAGATGACCGAGAACGGAATCAGTCCAATGTCACCCTCCTCTTTGCGCCACTGACTGGAGAAGGGACT
 620 -  R  V  L  K  D  D  R  E  R  N  Q  S  N  V  T  L  L  F  A  P  L  T  G  E  G  T
2107 — CTCTGCTGTGTCACGGGCATTGTAGTTCAGCACCAGACCACAGGAGGTGCTGTGTGGATAGAACAGCTTGGCCTCGTA
 646 -  L  C  C  V  T  G  I  V  V  Q  H  Q  T  T  G  G  A  V  W  I  E  Q  L  G  L  V
2185 — TCCACATACACCTTTCCCTGGAGAGAGGAGGTCCACACCGTCACCGTCATGGCCATCAACTCCCTGGGGCCCTCAACG
 672 -  S  T  Y  T  F  P  W  R  E  E  V  H  T  V  T  V  M  A  I  N  S  L  G  P  S  T
2263 — AGGAACACCCACATGACGCTAATGAGAAAAGCCAGCAAACCGAGGTCCGTCTCCTCGTTCAGCTCGGTGATGATTAAC
 698 -  R  N  T  H  M  T  L  M  R  K  A  S  K  P  R  S  V  S  S  F  S  S  V  M  I  N
```

```
2341 — GACAGCTGCGTGGCGCTGGTGTGGAGCCTGTTCCCCAACTCCTCTGCCCCGGCGTCGTTCGTGGTGGAGTGGAGCAGC
 724 -  D  S  C  V  A  L  V  W  S  L  F  P  N  S  S  A  P  A  S  F  V  V  E  W  S  S
2419 — CGGAGCCGGGGGAGGGGCCGGGGCGACGCGTGGCTCAGGGTGAAATGGGTTCGAGTCTCTGCCCCCAGCCGCTCTCTT
 750 -  R  S  R  G  R  G  R  G  D  A  W  L  R  V  K  W  V  R  V  S  A  P  S  R  S  L
2497 — TATCTCCATGATAGATTCTATGTGTCTGAAGAATATCAGTTTGCTTTGCACCCAATCTTTGCTAATGGAGAAGGAGAG
 776 -  Y  L  H  D  R  F  Y  V  S  E  E  Y  Q  F  A  L  H  P  I  F  A  N  G  E  G  E
2575 — CCATTCTACAATAAAGAAGACAGGGGGCGCCCCAGTGCCCAGCATGCTGCCTATGCCCTCCTGCTGATTATCGCCTTC
 802 -  P  F  Y  N  K  E  D  R  G  R  P  S  A  Q  H  A  A  Y  A  L  L  L  I  I  A  F
2653 — ATGTCGGTGGTGCTGTTTCTGACTCTGGCTGCATCCCAGCGACAGATGATGAAGCTTGTTTGGAAGGATGTTCCTAAT
 828 -  M  S  V  V  L  F  L  T  L  A  A  S  Q  R  Q  M  M  K  L  V  W  K  D  V  P  N
2731 — CCCAACAACTGCTCTTGGGCCCAAGGTGTGGACTTTAGGAAGGCAGAGGCCGTTGAGAACCTGTTCAGACACCCCGAG
 854 -  P  N  N  C  S  W  A  Q  G  V  D  F  R  K  A  E  A  V  E  N  L  F  R  H  P  E
2809 — CGCCTGACCTCCTGTCCCCTCCTTCTGGAAATGGAGACCATCTCGGAGGCGGTGATTGTGGAGAAGGCCCACCCGAAA
 880 -  R  L  T  S  C  P  L  L  L  E  M  E  T  I  S  E  A  V  I  V  E  K  A  H  P  K
2887 — GCTGCGTCGGAGAAGGACCGGGCGGCGTGGGCGGAGAAGGCTCTGCACGCCGCGGTCACCCCCTTCTCCTCGGCAGGC
 906 -  A  A  S  E  K  D  R  A  A  W  A  E  K  A  L  H  A  A  V  T  P  F  S  S  A  G
2965 — AGCTCGGCGCAGTCCAGCGTCGCCTACGCCACGGTCCTGCCCGCCGCGGAAGGGGGCCGCGCCCGCCGGAGGCCCCAG
 932 -  S  S  A  Q  S  S  V  A  Y  A  T  V  L  P  A  A  E  G  G  R  A  R  R  R  P  Q
3043 — GAGAGCCTCAGTAGCTGCTCCGACGAGGGCAACTTCTCTGCCGACACCTCGGACATGTCCGGGTCCTACCCGGGGGCG
 958 -  E  S  L  S  S  C  S  D  E  G  N  F  S  A  D  T  S  D  M  S  G  S  Y  P  G  A
3121 — CTGTGGGAGCCGGAGGGCATGCCGTCGAACCCGCGCCACCCGTGCCACTCCTGCGCCTCAACCGAGGAGTTCTCGGAG
 984 -  L  W  E  P  E  G  M  P  S  N  P  R  H  P  C  H  S  C  A  S  T  E  E  F  S  E
3199 — AACTCCGACCAGGAGGACCAGTCTCTGGACGGGACGGGCTCGAGGCAGGACTTGTACTATCTGGGGACGATCTCGCAG
1010 -  N  S  D  Q  E  D  Q  S  L  D  G  T  G  S  R  Q  D  L  Y  Y  L  G  T  I  S  Q
3277 — AGCGAAGAGGGGGAGGAAGGGAAACCATTTCACAGTGAGTCAACCCTGGGGCGCTGTCTGGAGTCCAGCCCCCTTCTC
1036 -  S  E  E  G  E  E  G  K  P  F  H  S  E  S  T  L  G  R  C  L  E  S  S  P  L  L
3355 — GGTCAACAGGAGCCCTGGTCCCATGGGGAGGAGAGGTCGGGGAAGGGTGTCCCACTCTACATGCCTCAGTTCCAAACA
1062 -  G  Q  Q  E  P  W  S  H  G  E  E  R  S  G  K  G  V  P  L  Y  M  P  Q  F  Q  T
3433 — GTGGTCACAGAAAGTCAAAGCACGAAAGGGCTGTGAACTTGCTTACGTGAAGCCGAAATGTGCTTTTGAATGAATGTT
1088 -  V  V  T  E  S  Q  S  T  K  G  L  *
3511 — TACCAGTGGATAGAACTTGGTTCTGAATCATAACTGGTGGCAAAAGATGATTGCAGGACTCCTGGGTCCTGGGCAACA
3589 — CACTAGTGTTGTACTCAGGAGGATGGTAAATATCCGTCTGTTTAAATGAATTGCTTGTAAAACCTGAGCTGTTAATCG
3667 — CCCTCTTTAAAGGTGTCTGCTCATTTCAACAAACAACACAGGCTACCGGGGCTTGGATCAATTCTGAAATTGCCTTAA
3745 — TATCTTTTTGGAACTGGCACACGCACATTAATTCTTAATGTTTCCTTATATTTTGTACACCTACAATGCTAGAAAATA
```
