# Supplementary material for: Duplicated Leptin Receptors in Two Species of Eel Bring New Insights into the Evolution of the Leptin System in Vertebrates
Source: PLoS One. 2015 May 6;10(5):e0126008. doi: 10.1371/journal.pone.0126008 (PMC4422726; doi:10.1371/journal.pone.0126008)
Supplement: S8 Fig — (PDF) [file pone.0126008.s008.pdf]

Fig S4 Spotted gar LEPR

1 – TTTTCTGTTTGAACATCCTGTGCTGGGTTGATGGAAACCTGGAACATTTAATTTGTAATCTGAAGCCACACACAAAA
1 - F C L N I L C W V D G N L E H L I C N L K P H T K
78 - TATCCAGACAGGAGCAGGTTCCTTACAATTAGGCTGCGGTATTCATTTGTTTCTTCTAAACAAAAAGAAGAGATTGTT
26 - Y P D R S R F L T I R L R Y S F V S S K Q K E E I V
156 – TATGCTGCTGAAAGTAGCTGTGAAGGAGAGGACACCTTACGATGTGCTATACCTCTAAAAAGCCTGAATAGCACCTAT
52 - Y A A E S S C E G E D T L R C A I P L K S L N S T Y
234 – ACCTTGAGAGTTACAATCTCAGATGGCAAAAGCGCTATGGAATCACCAGCGATGCACATTGTCCCTGGAAATGTGGTG
78 - T L R V T I S D G K S A M E S P A M H I V P G N V V
312 – AAGCCAGACCCGCCTCTCAAATTAGAGTATCACATGACCCTTGAAGGGGAGCTGTGGTTGAACTGGACAGAACCTCAG
104 - K P D P P L K L E Y H M T L E G E L W L N W T E P Q
390 - CCAGCTCCCCACAAACTGAAGTACGAAGTCAGATACTCTGCAGATACGGCTGAGGACAGCTGGTTGTATACAGAAGGT
130 - P A P H K L K Y E V R Y S A D T A E D S W L Y T E G
468 – TTGATGGCATCCCATGTGCTACTTAATAACCTGAGACCTGGTGTGAATTACAGTGCTCAGGTTCGCTGTAGGAATGGC
156 - L M A S H V L L N N L R P G V N Y S A Q V R C R N G
546 – AATAAATCTGGTCTCTGGAGTAATTGGAGTATCCCTCTCTTTATTTATTTGAATGAAGTGACATACCTTCCTGCAAAA
182 - N K S G L W S N W S I P L F I Y L N E V T Y L P A K
624 - ATACTGGCAAGTACTGGATCAAATGTAACAATTCACTGTGTATTTAACAACCGGAGCACTAATGCAACAAATATTGTG
208 - I L A S T G S N V T I H C V F N N R S T N A T N I V
702 - TGGTGGCTCAATACCCATGAAAAAGTTCCCGTAAGTCAATATTCTACCATTAATGATCACGTGAGCAGTGTCACTCTG
234 - W W L N T H E K V P V S Q Y S T I N D H V S S V T L
780 - GTTAATGTGAAACCACATAAACGCAAAGGGGGGATTGACTATGATGTGCTACAATGCTGTCAACAAAATGGAGAAAAT
260 - V N V K P H K R K G G I D Y D V L Q C C Q Q N G E N
858 – TCAGTCTGCAACTACCGCTACGCGGCAATTTATGCAAAAGATATCAATGTTACTATCTCCTGTGAAACTAATGAGCAT
286 - S V C N Y R Y A A I Y A K D I N V T I S C E T N E H
936 – CTGACCAACATGACTTGCAAATGGAATCCAAATTGGGTTTTGGGAAGTGATGTGAAGTTCCTTTATCGGAAAGGCCTT
312 - L T N M T C K W N P N W V L G S D V K F L Y R K G L
1014 – TCCTGTGGTGCTCTGGAAGAGGATGTGCGTGTCTCCCGCGTGGAGGAGTGCCCTGTCATGGGATGGGGATCCCACAAG
338 - S C G A L E E D V R V S R V E E C P V M G W G S H K
1092 – TGCACCTTCCAACCATTGTACCTGTCTTTCTGTTACGTGATGTGGCTGGAGATTCACCATGAGCTTGGCCCTGTGAAA
364 - C T F Q P L Y L S F C Y V M W L E I H H E L G P V K
1170 – TCACGCCCCACCTATGTCACCCCAATGGATGTTGTAAAGCCACATCCTCCCCTCAACCTTGAGGCTGAGATCACAGTT
390 - S R P T Y V T P M D V V K P H P P L N L E A E I T V
1248 – CCAGAGGGACATCTGAGTATCAGCTGGCAAAGGACGGAATTGCCAGTTTATGATCTTCAGTTTGAGATCCGCTATGCT
416 - P E G H L S I S W Q R T E L P V Y D L Q F E I R Y A
1326 – GTAGATGGGCCGAATAAGCAGTGGAAGCTTCTCAGAACAGTATATAATGAGACAGTTGTGACTCAGGTGGCCGATCCA
442 - V D G P N K Q W K L L R T V Y N E T V V T Q V A D P
1404 – TGTGTTGTATACACGGTTCAGATTCGCTGCAAAAGGTTCAATGGACCTGGACACTGGAGCACATGGAGCAGCCCTGTG
468 - C V V Y T V Q I R C K R F N G P G H W S T W S S P V
1482 – TACACTGTTGTTCATGATATTCAAGCTCCTGAACAGGGACCTGACTTTTGGAGAATGATTCGTGAGGATCCAGCAATG
494 - Y T V V H D I Q A P E Q G P D F W R M I R E D P A M
1560 – AAGCAAACTAATGTTACTCTTCTAATTAAGCCACTGAGAAAGGAAGACCTCTTTTGTTCTGTGGATGGGTTAATTGTT
520 - K Q T N V T L L I K P L R K E D L F C S V D G L I V
1638 - CAGCATCAAACATCAAGTGATGTGCTACGGTATGAGTATCTGAATATCACCACAACATACAGTTTTCCCTGGATTGAA
546 - Q H Q T S S D V L R Y E Y L N I T T T Y S F P W I E
1716 – GATGTTCACAGTGTGTCAGTCCTGGCCTTCAACTCAGTTGGCTCTTCTGTGATGAACTATAATTTAACATTCACAAGG
572 - D V H S V S V L A F N S V G S S V M N Y N L T F T R
1794 – CACACAAGAAAAGTACAGACTGTTCAGTCGTTCAGCTGTGCAATGGTAAACAGCAGTTGTGTGGCACTTTCGTGGAAC
598 - H T R K V Q T V Q S F S C A M V N S S C V A L S W N
1872 – CTGCTTCCAAACCGCTCTCTTCCCGAGTCTTTCATTATCGAGTGGAAACTTCAAAACAGGGAGAAACAGCTACAAAAC
624 - L L P N R S L P E S F I I E W K L Q N R E K Q L Q N
1950 – ACAGATGAAGTCGTTAAGTGGGTTCGAGCACCTCCAAAGATACACACCTTTTATTTATATGACACTTTCTTCCTTTCT
650 - T D E V V K W V R A P P K I H T F Y L Y D T F F L S
2028 – GAGGAATACCTGTTTATTTTGTATCCAATATTTCTGGAAGGAGAAGGGGAACCCATCTATAAGGACAAGAGGAAACCC
676 - E E Y L F I L Y P I F L E G E G E P I Y K D K R K P
2106 – AAAGGAGAGCAGGCTGCTTACATTCTTCTTCTCCTTATTGCATTTCTGTCTGTTGTGCTGTTTGTGACACTTGCAGTC
702 - K G E Q A A Y I L L L L I A F L S V V L F V T L A V
2184 – TCTCAGCATCAAATGAAGAAGCTGGTTTGGAAAGATGTTCCTAATCCTAATAACTGCTCCTGGGCACAAGGAGTTGAT
728 - S Q H Q M K K L V W K D V P N P N N C S W A Q G V D
2262 – TTTAAAAGGGCAGAAACCATAGAGAATCTCTTTAAACACCCAAAGAGAGTAACATCATGTCCTCTTCTCTTGGAATCT
754 - F K R A E T I E N L F K H P K R V T S C P L L L E S
2340 – GAAACCATTTCTGAGGCTGTGATTCTAGAGAAGATGAAACTGGGTTCACAGGAACAAACTACCTCAGATGCATCCCTG

```
 780 - E T I S E A V I L E K M K L G S Q E Q T T S D A S L
2418 - GGCAAAAAACAGGAACTGGACGAGGACTCTACCCATTCTGCTTGCCAGTACAATGAGGAATCCTTAGTCCAAGAAAGG
 806 - G K K Q E L D E D S T H S A C Q Y N E E S L V Q E R
2496 — TCTATGCACTCCACTACCCCAGAGAGATCAGCACAGTCCATAATACAGTATGCAACAGTGTTACTTCCTGATGTGCCC
 832 - S M H S T T P E R S A Q S I I Q Y A T V L L P D V P
2574 — ATCCATCTCTACAAACAACAGAAAAGTATCAGCAGTTCTTCTGATGAGGGCAATTTTTCTGCAAACAACTCTGACATT
 858 - I H L Y K Q Q K S I S S S S D E G N F S A N N S D I
2652 — TCAGGATCCTTTCCCAACAATCTGTGGGATGTTGAAAATCAATCTTGTAAGAATTCAGCACAAGAAAATCCTAGAAAT
 884 - S G S F P N N L W D V E N Q S C K N S A Q E N P R N
2730 — ATCTGCTCCTTTAATTCTACAGAAGAGCTTTCAGAAACGTCTGATCAGGATGACTATGTTCTTGATGAAATGTGCACA
 910 - I C S F N S T E E L S E T S D Q D D Y V L D E M C T
2808 — GGAAATGATCTATACTACGTTGGTGTGGCTTCTAATGATGAGGAAAATGGGGAAGAGGAGAAAGAAAGTTTCCTAATG
 936 - G N D L Y Y V G V A S N D E E N G E E E K E S F L M
2886 — GAAAATTCTCATGTGCCTTCTCAAGACAGAGATGAGATTATGCAAGAATCAAATCCTTTATTAGGCTGCCATTCTTTT
 962 - E N S H V P S Q D R D E I M Q E S N P L L G C H S F
2964 — CTGAATGTAAAAGTGAACAAGAAAGATGTGCCTGCAAAGAATATCCCTTTGTACATGCCTCAGTTCCAAACATCGTCC
 988 - L N V K V N K K D V P A K N I P L Y M P Q F Q T S S
3042 — AATAAAATTCTGAAAGCCAAAGCTCAA
1014 - N K I L K A K A Q
```
