# Supplementary material for: Duplicated Leptin Receptors in Two Species of Eel Bring New Insights into the Evolution of the Leptin System in Vertebrates
Source: PLoS One. 2015 May 6;10(5):e0126008. doi: 10.1371/journal.pone.0126008 (PMC4422726; doi:10.1371/journal.pone.0126008)
Supplement: S9 Fig — The sequences were aligned by Clustal Omega and manually adjusted. The amino acids presenting similar physico-chemical properties are shaded in the same color. (PDF) [file pone.0126008.s009.pdf]

```
                                1
Homo_sapiens_LEPR               MICQKFC--- --------VV L-----LHWE FIYVIT---- AFNLSYPITP
Macaca_mulatta_LEPR             MICQKFC--- --------VV L-----LHWE FICVIT---- AFNLSYPITP
Bos_taurus_LEPR                 MISQKFC--- --------VA L-----LHWE FIYVIT---- AFNLAYPITP
Sus_scrofa_LEPR                 MTCPKFS--- --------VA L-----LHWE FIYVIT---- AFDLAYPITP
Rattus_novegicus_LEPR           MTCQKFY--- --------VV L-----LHWE FLYVIT---- ALNLAYPTSP
Gallus_gallus_LEPR              MYHQIIL--- --------TM S-----LLLG FLHVAA---- AHCMVHEIHP
Ornithorhynchus_anatinus_LEPR   VKCYRAS--- --------VV V-----CRSN FIQLLV---- ARDTTFQIPP
Xenopus_tropicalis_LEPR         MFWHWIL--- --------PF F-----LLLR YMQITA---- AYSEMYWTPP
Anolis_carolinensis_LEPR        MNWYNIL--- --------TG F-----LQLN FFQMAI---- SHCMVYQIPP
Chrysemys_picta_bellii_LEPR     MYSHKLL--- --------TV F-----LHLD FIRMAV---- AHCMVHQIPA
Latimeria_chalumnae_LEPR        MFWFTLL--- --------TG L-----LQHG VST------- ---LTIAYDP
Danio_rerio_LEPR                MFFSDVLSCP PRSVFIMLAL LVIFIAVSQG LADLNPSDGV S-DGVYEDLK
Carassius_carassius_LEPR        ---------- MMSLFIMLAL LVNFITISQG LAALSPSDGR --GGVYEDLK
Takifugu_rubripes_LEPR          -------MSS TMFGRVTLSV MVLGFLLSRG VLSLENSDAG GRHSGVLDLP
Orizias_latipes_LEPR            ---------- -MVRAAMLVV LIQILLIPHG AQYLKPADGA SNHLGPLGLP
Salmo_salar_LEPR                --------MK TMIMSAMLTF LVHILIVSHG AVSVEPMGVS P-HGDLLDLP
Oreochromis_mossambicus_LEPR    -------MTA TMVQSVMLAG LVYVFLVSYG AQSLKPEDGA SLRSGAVDLP
Tachysurus_fulvidraco_LEPR      ---------- -----MRLFV IVNVFTASRA AVDVRPLNGW --PGVYHDLQ
Epinephelus                     -------MTT TMVRSVMLTV LMHFFLVPHG AQCLEPENGA SDHSGALDLP
Anguilla_anguilla_LEPRA         ---------- -MSFSTMGMF LLCLILALHS VVGVTLSDG- ----RLLGTP
Anguilla_anguilla_LEPRB         MHWLN--SFY SGPFSAKGMF AVSFLLTLHG VVAVHSAVA- --PPPEFEYP
Homo_sapiens_GCSFR              ---------- ---------- ---------- ---------- ----------
Danio_rerio_GCSFR               ---------- ---------- ---------- ---------- ----------

                               51
Homo_sapiens_LEPR               WRFKLSCMPP NSTYDYFLLP AGLSKNTSNS NGHYETAVEP KFNSSGTHFS
Macaca_mulatta_LEPR             WRFKLSCMPP NSTYDYFLLP AGLSKNTSNL NGHYETAVE- -FNSSDTHFS
Bos_taurus_LEPR                 WKFKLSCMPS NTTYDF-LFP PGISKNTSNL NGRYEAVVET KLNSSGTYLS
Sus_scrofa_LEPR                 WKFKLSCMPP NTTYDF-LLP AGISKNTSTL NGHDEAVVET ELNISGTYLS
Rattus_novegicus_LEPR           WRFKLFCAPP STTDDSFLSP AGVPNNTSSL KGASEALVEA KFNSTGIYVS
Gallus_gallus_LEPR              RSFTLPCLLL NETSSSP-SA AGVVRSRSGW RREHG----- ---VAETNPS
Ornithorhynchus_anatinus_LEPR   WSFTWSCVAP DQTSEFPVAV EGSW-SGRRL TGDQG-AMEA RSNWSDASLL
Xenopus_tropicalis_LEPR         SDFFLTCILT NKSVNYP-LL AGILQNKSDI SGKYD-TLEG RSDFRCTHLA
Anolis_carolinensis_LEPR        QSFQLPCVQP QKISNFS-LS PEIVENSPSS NKEHG----- ---TSKINAT
Chrysemys_picta_bellii_LEPR     SSFMLPCMSP NETSVIP-LT AGVA-SGPGL HGEHG----- ---TAETNSL
Latimeria_chalumnae_LEPR        WNFTLSCNLQ NKNSVTPQEN T-QADRVQSL HDDHGETT-- ---NASTGPP
Danio_rerio_LEPR                WKALLCCDHP V-QTLN---- ---------- ---------- ------SGLS
Carassius_carassius_LEPR        WKALLCCELP FAQTLD---- ---------- ---------- ------DGLS
Takifugu_rubripes_LEPR          WKDELCCESR PAS-TV---- EG-------- ---------A SAPAERPNGS
Orizias_latipes_LEPR            WQDELCCDSP SAYLGE---- DR-------- ---------G VTNRSKTNGT
Salmo_salar_LEPR                WQTELCCSSR PAQLHN---- RG-------- ----KEEDRG SVGS-RATPS
Oreochromis_mossambicus_LEPR    WQDELCCESP SAHLGV---- EG-------- ---------G SANSPEANLS
Tachysurus_fulvidraco_LEPR      WRVQLCCALT SNGKAN---- ---------- ---------- ------QSLS
Epinephelus                     WQDELCCDSP SAHLTA---- EG-------- ---------D DMHAPETNHS
Anguilla_anguilla_LEPRA         WAAALCCDLA EQA------- ---------- ---------- ----------
Anguilla_anguilla_LEPRB         GKLGLCCYLG DGWAEE---- TG-------- ----GGNGEG DNSRDGMGGG
Homo_sapiens_GCSFR              ---------- ---------- ---------- ---------- ----------
Danio_rerio_GCSFR               ---------- ---------- ---------- ---------- ----------

                              101
Homo_sapiens_LEPR               NLS-KTTFHC CFRSEQDRNC SLCADNIEGK TFVST-VNSL VFQQID-ANW
Macaca_mulatta_LEPR             NLS-KTTFHC CFRSEQDRNC SLCADNIEGK TFVST-VNSS VFQQMG-ANW
Bos_taurus_LEPR                 NLSSRTTFHC CFWSEEDKNC SVYTDDIEGK AFVTT-VNSL VFQQTG-ANW
Sus_scrofa_LEPR                 NLSSKTTFHC CFWSEEDKNC SVHADNIAGK AFVSA-VNSL VFQQTG-ANW
Rattus_novegicus_LEPR           ELS-KTIFHC CFGNEQGQNC SALTGNTEGK TLASV-VKPL VFRQLG-VNW
Gallus_gallus_LEPR              LLMDEESFLC CLWSNNNASC SLYSANMQAR MLIPSEMSIS ASQERD-SNW
Ornithorhynchus_anatinus_LEPR   LLESKESLRC CLWSSQQKNR SLPGDGLEEK SCISSPVPTS ALQPTGIASW
Xenopus_tropicalis_LEPR         DLKSQGRVSC CLWDHLNTNF SSQNGVAELK EMLFASFA-S D-IQLEDFSW
Anolis_carolinensis_LEPR        ALAAEDKFLC CHWSEIYINC FIYTAEMEAR TLTYSEPGAL SSHQID-SLW
Chrysemys_picta_bellii_LEPR     VLVAEESFLC CLWGETHANC SLYTAGMETK KFTSSEISSL APQQTD-LSW
Latimeria_chalumnae_LEPR        KLMNVDDISC CSWGDTDDHC SQQFHSMEPE ---------- ------SMNW
Danio_rerio_LEPR                E--HPPEQHC QLLNATKQQ- ---------- ---------- ----SFGSCL
Carassius_carassius_LEPR        D--HPPVGQC QLQNATKPE- ---------- ---------- ----FSRNCL
Takifugu_rubripes_LEPR          NRSLPHDSQC SFKNLTSKLH ---------- ---------- PLE-FSGTCL
Orizias_latipes_LEPR            ISSLRHLPRC KYRRLTPESL ---------- ---------- PQKPSEGNCL
Salmo_salar_LEPR                DPGHPHTLQC LFRNFTSILG PHP------- ---------- QTEPSRATCW
Oreochromis_mossambicus_LEPR    QSNLPHSPGC TFKSSRIDSH ---------- ---------- HPRSSGGTCL
Tachysurus_fulvidraco_LEPR      HINSPHQYQC HIQNSTNTTD DSS------- ---------V SQVVSENIRL
Epinephelus                     ESNLPQHPLC SFRGSTSESH ---------- ---------- PHEPSGGTCL
Anguilla_anguilla_LEPRA         -----RSPRC HISPHSSSLA SLN------- ---------- HLPSRGVVCL
Anguilla_anguilla_LEPRB         SLGHTPPGRC SVSSPARFRQ SAL------- ---------- TSQNHSVVCL
Homo_sapiens_GCSFR              ---------- ---------- ---------- ---------- ----------
Danio_rerio_GCSFR               ---------- ---------- ---------- ---------- ----------
```

```
                               151
Homo_sapiens_LEPR              NIQCWLKGDL KLFICYVESL FKNLFRNY-- NYKVHLLYVL PEV-LEDSPL
Macaca_mulatta_LEPR            NIQCWLKGDL KLFICYVESL FKNPFKNY-- KHKVHLLYVL PEV-LEDSPL
Bos_taurus_LEPR                NIQCWMKEDL KLFICNIESL FKNPFKNY-- DLKVHLLYVL LDV-LEESPL
Sus_scrofa_LEPR                NIQCWMKEDL KLFICYMESL FKNPFKNY-- DLKVHLLYVL LEV-LEGSPL
Rattus_novegicus_LEPR          DIECWMKGDL TLFICHMEPL LKNPFKNY-- DSKVHLLYDL PEV-IDDLPL
Gallus_gallus_LEPR             NIECWVEGKL DLLVCSLQFP KFH--VRL-- DMKVHLLYAV SELSLGDAST
Ornithorhynchus_anatinus_LEPR  NILCWTKGDL KTFICHLTTF SKISFKSY-- DFRLRLLYVM SELPPEDSSM
Xenopus_tropicalis_LEPR        KVQCSFEEKA NTLICDLQLL PETKHIVT-- DYRISLHYSL VAK-------
Anolis_carolinensis_LEPR       NLQFWTQEST QELLCIVELS AKSPYWNE-- DLTVNLHYAL TDVSLEATST
Chrysemys_picta_bellii_LEPR    NIQCWTKGDL EQFVCILKLS NKKSYLNG-- EFKINLLYVL SELSLEDMST
Latimeria_chalumnae_LEPR       ITLCWTDGNL EHLVCHLKPT YKKTYFSG-- ICRTKYIHNL SELTVEELPV
Danio_rerio_LEPR               DILCWLEGER ENLICNAKTR RAAAAAAST- --LVSVSPHQ LVVQMDV---
Carassius_carassius_LEPR       DILCWLEGER TNLICNAKSH RAAAATS--- --LLTI-RPQ LVVQMNV---
Takifugu_rubripes_LEPR         DILCGIDEKW ENVTCHLEPH ALPLSLPDAG HMAVSLQRRF QKSQSRV---
Orizias_latipes_LEPR           EILCQINEKW ENLTCYLQP- ---SRKLDTG GMTFSFQQLP DKD-------
Salmo_salar_LEPR               DILCRVDETW DNVICDLKHP ATSSDTSTPG SVALSLQHLA TL--------
Oreochromis_mossambicus_LEPR   DILCRIDGNW QNLICDLRSR GQPSDS---- LMAVSLQRQL FQEDG-----
Tachysurus_fulvidraco_LEPR     DIFCRLEDEQ ANVICLLKHQ RTSATDASH- --LIVSL-WR VTLESDP---
Epinephelus                    DILCRIDENW ENLTCDLQSH SQPSTTLDAG LMAVSLQRLL SQKDAEV---
Anguilla_anguilla_LEPRA        NILCWVDGNL ENVICNLKLY VGLSDTSR-- PIAISLQRLP SQFGSD----
Anguilla_anguilla_LEPRB        DVLCWVTGDR RHLVCERNPR GGNVAVGG-- VVTLSLLRLQ SDQQMQATP-
Homo_sapiens_GCSFR             ---------- ---------- ---------- ---------- ----------
Danio_rerio_GCSFR              ---------- ---------- ---------- ---------- ----------

                               201
Homo_sapiens_LEPR              VPQKGSFQM- VHCNCSVHEC CECLVPVPTA KLNDTLLM-C LKITSGGVIF
Macaca_mulatta_LEPR            VPQKGSFQM- VHCNCSVHER CECLVPVPTA KLNDTLLM-C LKITSGGVIF
Bos_taurus_LEPR                LPQKDSFQV- VQCNCSVHEC CECHVPVPTA KLNDTLLM-Y LKITSGGAVF
Sus_scrofa_LEPR                LPQKGSFQS- VQCNCSAREC CECHVPVSAA KLNYTLLM-Y LKITSGGAVF
Rattus_novegicus_LEPR          PPLKDSFQT- VQCNCSVRE- CECHVPVPRA KVNYALLM-Y LEITSAGVSF
Gallus_gallus_LEPR             SSLKRTALA- AQCNCSEYGK CECHVPS--P RLNHTYVM-W LKTVIGVTPL
Ornithorhynchus_anatinus_LEPR  MPQKDSFKV- IPCNCRVPEG CECEIPLPAT KLNCTHIL-Y LEILNEVTSL
Xenopus_tropicalis_LEPR        ----SELKGT AECRCFGYEK CECIVPS--V KFNDTYIL-W IEILNITALL
Anolis_carolinensis_LEPR       HSLKDNFTV- THCNSIRDQK YACQISS--V KLNHTYIM-W LKITNGIALL
Chrysemys_picta_bellii_LEPR    GSLKGNFMV- TPCNSNGSDK HECHIPS--L KLNYTYIT-W LNIINGVTLL
Latimeria_chalumnae_LEPR       KYQKKSLVVV NQQSCLGHDN FECIIPS--V KLNYTYFM-W MEITTNAGML
Danio_rerio_LEPR               HSDET----N STAQCVGEET AICSVSLHG- -GDATVSL-V IIISENGTTA
Carassius_carassius_LEPR       RSDEIMSTGS HAAQCAGEET VMCSVSLHG- -NDATVLL-T ISITLNETTA
Takifugu_rubripes_LEPR         --DSEEAASD PPVFCEAEDS FTCSVALDA- -ESSFHAVVT VTIAD---AR
Orizias_latipes_LEPR           ----GTEVNS NPVVCEAEES FTCSLPLHP- -AASFVTTVT VNLSS---VV
Salmo_salar_LEPR               --PDSEVNTT HGTDCVGEDS ITCSIALHV- -VSSIVVV-T ANVSNT--TA
Oreochromis_mossambicus_LEPR   ----DYPASE NPVVCEAQDS FMCSLTLDP- -TTSFVAMVT VSISD---AV
Tachysurus_fulvidraco_LEPR     LTN--ETNIT TQVQCPGEDE ITCFFVLQP- -NDVSVSL-S VSGFLGGRPL
Epinephelus                    --DDGNAATD NPVVCEAKDS FMCSVALDT- -KTSFVTRVT VSISD---AV
Anguilla_anguilla_LEPRA        NVTDSG---- AHSKCEGQDV RTCSVALHS- -VNSTVSL-M ISISDGSSSV
Anguilla_anguilla_LEPRB        SALPDGVTHS CQEEGEGVGV FWCALPPGS- -MRDAVTL-R VNVARGNRSA
Homo_sapiens_GCSFR             ---------- ---------- ---------- ---------- ----------
Danio_rerio_GCSFR              ---------- ---------- ---------- ---------- ----------

                               251
Homo_sapiens_LEPR              QSPLMSVQPI NMVKPDPPLG LHMEITDDGN LKISWSSPPL VPFP--LQYQ
Macaca_mulatta_LEPR            QSPLMSVQPI NMVKPDPPLG LRMEITDDGN LKISWSSPPL VPFP--LQYE
Bos_taurus_LEPR                HSPPMSAQPI NVVKPDPPLG LRMEITDTGS LKISWSSPTL VPFQ--LQYQ
Sus_scrofa_LEPR                HSPLMSVQPI NVVKPDPPLG LHMEITDTGN LKISWSSPTL VPFQ--LQYQ
Rattus_novegicus_LEPR          QSPLMSLQPM LVVKPDPPLG LRMEVTDDGN LKISWDSQTK APFP--LQYP
Gallus_gallus_LEPR             WSPLMSVKPI DIVKPEPPLN VRLEMTERGQ VKICWSEPVP MPYP--LRCE
Ornithorhynchus_anatinus_LEPR  QSPIMATQPI NVVKPDPPIR LQMEMVEYRQ LKVSWLSPPL SPYP--LRYQ
Xenopus_tropicalis_LEPR        HSPPMSVVPY HIVKPDPPDD LRAEIMEQGT LKVFWLKPIS AAYE--LQYQ
Anolis_carolinensis_LEPR       QSPLMSVKPI DIVKPEPPLH LKMEMTDKGQ LKISWSSPAS KSYP--LQYE
Chrysemys_picta_bellii_LEPR    QSPLMSVRPI NIVKPEPPLN LRLEMTDKGQ LKICWSNPVL TPYP--LQYE
Latimeria_chalumnae_LEPR       QSPLMSIMPI DIVKPNPPLN LQGDITNEGL LRINWTTPDP LPYE--LQYE
Danio_rerio_LEPR               QSQKMQVSTY ELQAGDPSRE LKPSPLSMKS PVFKHFG-FP VSYV--DNVR
Carassius_carassius_LEPR       LSPKMQVSTH HLRRPDAPVN LHYNVTTEGE VIFRWSDTQP DSYA--VNYE
Takifugu_rubripes_LEPR         APSVLLRVPA RPVKPAPPVN LSHVQTIEAE LILHWGDPKD I-KTDLLQYE
Orizias_latipes_LEPR           APPVLLIIPA RPVKPSPPVN VTHYQTIEAE LFVQWESPPH F-DAAQLRYE
Salmo_salar_LEPR               GPLVMLSVPP RLWKPSPPLN LTHTQTTEGE LILSWSDPQP HASPVQLSYE
Oreochromis_mossambicus_LEPR   APPVLLRVPA RPEKPSPPGN LSHIQTIEAE LIVLWDDPAD F-DAGPLRYE
Tachysurus_fulvidraco_LEPR     QTPEMRISTD LLRKPEAPFY LRYNVTTEGE VMIAWNDSQN NKLP--LYYE
Epinephelus                    APPVLLRIPA RPVKPSPPVN LLHNQTIEAD LILQWDDPSD S-DTGPLRYE
Anguilla_anguilla_LEPRA        QSPVMDIVPL SFLKPDPPLN LQYHMTIEGE LRLSWTHALP SAER--FVYD
Anguilla_anguilla_LEPRB        LSPEISFVPQ KLVRPDPPVK LWYNMTTEGE LRLHWTPPQP VTGP--LTYD
Homo_sapiens_GCSFR             ---------- ---------- ---------- ---------- ----------
Danio_rerio_GCSFR              ---------- ---------- ---------- ---------- ----------
```

```
                            301
Homo_sapiens_LEPR           VKYSEN--ST TVIREADKIV SATSLLVDSI LPGSSYEVQV RGKRLD---G
Macaca_mulatta_LEPR         VKYSEN--ST TVIREADKIV SATSLLVDGI LPGSSYEVQV RGKRLD---G
Bos_taurus_LEPR             VQYSEN--ST KYIRKTDEIV SATSLLVDSV LPGSSYGAQV RCKRLD---G
Sus_scrofa_LEPR             VKYSEN--ST TNMREADEIV SDTSLLVDSV LPGSSYEVQV RGKRLD---G
Rattus_novegicus_LEPR       VKYLEN--ST -IVREAAEIV SDTSLLVDSV LPGSSYEVQV RSKRLD---G
Gallus_gallus_LEPR          VNISGN--SD QNDWQVVQVA LNTSLDIDNM LLDSSSFAQV RCKSHC---G
Ornithorhynchus_anatinus_LEPR VKYSVN--TT KTAKQVAEVV SATSLLVDDV LPGSSYAVQV RAKRLQ---G
Xenopus_tropicalis_LEPR     VRYTVK--AA ETNSQVYLLV NETSVIISDI QPCTEMVIEV RCINLH---K
Anolis_carolinensis_LEPR    IKCFAN--ST KNVWQVVQIT LETSLIINNA LFDSSYNIQV RCKRHY---G
Chrysemys_picta_bellii_LEPR VKFSGN--AT QNAWQVVEIV IETSLIIGNM LAGSSYLVQV RCKSLH---G
Latimeria_chalumnae_LEPR    VKYSIN--ST DSTWQIVKVV MDTSLQLGSV QLGFLYLAQV RCKRLL---G
Danio_rerio_LEPR            VVDKKK-TQF CKLCYVLKVE GRSWVALNEL SSDIRYTVQV RCQN-----H
Carassius_carassius_LEPR    IRYS-S-NSS LQQWEMVKVK GRSWVPLNDL SSAIRYTVQV RCQS-----H
Takifugu_rubripes_LEPR      VRYSPD--TI HPAWQVMSVS GDTKTSL-DL KACVNYTVQV RRSSRS---D
Orizias_latipes_LEPR        VRYNTK---S DLAWQVVSVT GEPRLSL-DL QPEQEYTFQV RCSRLD---E
Salmo_salar_LEPR            VRYNTSQSTS HLNWLHVEVS GCQWVSLTGL RPGLHYTVQI RSHHPA---R
Oreochromis_mossambicus_LEPR VRYSSG--TT HPAWQVVSAP GEPKVSL-DL KPELKYSVQV RCSGPE---E
Tachysurus_fulvidraco_LEPR  LRYSP--NTS LTHWEVLNVQ -HPWVSLSEL TSGVRYTVQV RCKSLHHLHN
Epinephelus                 VRYSSN--TT HPQWQVVSAP EEPRLPL-EL KPRLNYTIQV RCSGLE---N
Anguilla_anguilla_LEPRA     IRYSS---SS LHSWMRMSAE GAPGASLKGL SVGLNYTVQV RCKTPG---N
Anguilla_anguilla_LEPRB     VRYSSN--TS LNSWVHVNKV ITQPVTLTGM NAGVTYTVQV RCKILG---K
Homo_sapiens_GCSFR          ---------- ---------- ---------- ---------- -------MAR
Danio_rerio_GCSFR           ---------- ---------- ---------- ---------- -------MAS

                            351
Homo_sapiens_LEPR           PGIW-SDWST PRVFTT---- --QDVIYFPP KILTSVGSNV SFHCIYKKEN
Macaca_mulatta_LEPR         PGIW-SDWST PHVFTT---- --QDVIYFPP KILTSVGSNV SFHCIYKNEN
Bos_taurus_LEPR             LGIW-SDWSA LLTFTT---- --QDVIYFPP KILTAVGSNI SFHCIYKNEK
Sus_scrofa_LEPR             PGIW-SDWST PFTFTT---- --QDVIYFPP KILTSVGSNI SFHCIYKNEN
Rattus_novegicus_LEPR       SGVW-SDWSL PQLFTT---- --QDVMYFPP KILTSVGSNA SFCCIYKNEN
Gallus_gallus_LEPR          PGFW-SEWST LYNLNV---- -GAEVLYFPT KILTSVGSNV SFHCIYKNKT
Ornithorhynchus_anatinus_LEPR PGLW-SDWSP SHALNI---- --QACFYFFP GITLLFQPSV NYHQRVKYKN
Xenopus_tropicalis_LEPR     SGLW-SDWSK TWVLNS---- --QDVFYFPQ KVLVSSGSST SVSCLFCDNG
Anolis_carolinensis_LEPR    SGLW-SDWSI PYSMNL---- --QDVMYFPQ KILASVGTNV SFYCISKTKD
Chrysemys_picta_bellii_LEPR PGFW-SDWST PYNLNT---- --EDVMYFPP KILTSVGSNV SFHCLYNDKN
Latimeria_chalumnae_LEPR    LGFW-SNWST PYKLDA---- --REVIYFPP RVLASAGSSV TISCLYNNKS
Danio_rerio_LEPR            LGYW-SEWSQ PFYFKL---- ---DVSYIPA EVFTTQESEV TVYSVFHNRS
Carassius_carassius_LEPR    FSYW-SEWSQ PFYFTL---- ---DVSYIPA EVFTAPGSEV TVSAVFHNRS
Takifugu_rubripes_LEPR      PPLW-SGWSE SHHIFL---- --DEVSYIPE KVVVKAGENV TVYCVFNDHN
Orizias_latipes_LEPR        PPLW-SEWSA PYKFYQ---- --YIVTYIPE KMVARAGESV TVYCLFNNRS
Salmo_salar_LEPR            PHLW-SDWSQ QHRIRL---- --ENVTYLPE RVVASFGDSV TVYCVFNDLR
Oreochromis_mossambicus_LEPR PPLW-SEWSE PHHIRL---- --DTVSYIPK IVVARPGENV TVYCVFNDHR
Tachysurus_fulvidraco_LEPR  WSEW-SNWSQ PFFLTL---- ---DVSYIPA EVFTRPGAEV TVYGVVHNHS
Epinephelus                 PPVW-SDWSE PYHIYL---- --DTVSYIPE KVVARPGDNV TVYCVFNDHS
Anguilla_anguilla_LEPRA     QGVW-SDWSR PLYVYL---- --NEVSYIPE RLFTSTGSNV TIYCIFNNRS
Anguilla_anguilla_LEPRB     PGLW-SEWSQ SLFIYL---- --HEVTYLPK SVFTSEGANV TVYCIFNNQS
Homo_sapiens_GCSFR          LGNCSLTWAA LIILLLPGSL EECGHISVSA P-IVHLGDPI TASCIIKQNC
Danio_rerio_GCSFR           VSLEIKLWMY IYVVIKVTGA SSCGNVYTPA P-VVLAGSPV SVSCSIEEDC

                            401
Homo_sapiens_LEPR           KIVPSK--EI VWWM-NLAEK IPQSQYDV-V SDHVSKVTFF NLNETKPRGK
Macaca_mulatta_LEPR         KIVSSK--KI VWWM-NLAEK IPQSQYDV-V SDHVSKVTFF NLNETKPRGK
Bos_taurus_LEPR             KIVSSK--KI VWWL-NLAEK IPQSQYDV-V DDHISKVTFP NLNATKPRGK
Sus_scrofa_LEPR             KIVSSK--KI VWWM-NLAEK IPQSQYDV-V GDHVSKVTFP NMNATKPRGK
Rattus_novegicus_LEPR       QTISSK--QI VWWM-NLAEK IPETQYNT-V SDHISKVTFS NLKATRPRGK
Gallus_gallus_LEPR          QSVASK--KI VWWL-NLAEE IPESQYTL-V NDRVSKVTLF NLKATKPRGS
Ornithorhynchus_anatinus_LEPR KYTPSQ--KC ELLPLFLRDV LP---LDS-I YCHCSCLSIS RMNEFKMNGS
Xenopus_tropicalis_LEPR     KKVPSS--NI TWWL-NFGEK IPKHQYRT-T SDYFSKVFLT HLNTTKPKGK
Anolis_carolinensis_LEPR    RIISSR--KI IWWL-NLAKE IPRSQYTI-V NDYISKVTLV NLSAMNPGGK
Chrysemys_picta_bellii_LEPR KMILSK--KI VWWL-NLAEE IPERQYTL-V NDRVSRVTLF NLSATKPRGK
Latimeria_chalumnae_LEPR    GT--VK--DV DWWL-NLAVK IPENQYTI-I NDHIASVTVT KLNATKNRGK
Danio_rerio_LEPR            W--SAS--KA VWFL-NGKMK IPESQYRV-I NEQVSTVTLK ----MDKA--
Carassius_carassius_LEPR    W--SAS--KA VWML-NGQVK IPESHYRV-I NEQVSAVTVT ----VDEP--
Takifugu_rubripes_LEPR      F--NAS--TA LWTL-NFDQE LDYSLYHP-I NQWVSQVT-- -MRPSETG--
Orizias_latipes_LEPR        M--NAS--EA VWKL-NFHQL LHS--SQS-V SGRVSKIT-- -MRASESR--
Salmo_salar_LEPR            V--NAS--TT VWIL-NSRDR LPKSQYTA-V NDRVSKIT-- -VRPSEQR--
Oreochromis_mossambicus_LEPR M--NAS--MA VWKL-NFKPP LQPTLYHP-V NQWVSKIT-- -VRPSENQ--
Tachysurus_fulvidraco_LEPR  R--TAS--KA VWML-NGL-V LPESQYQT-I NERVSAVTIR ----SKEP--
Epinephelus                 I--NAS--TA MWMR-NFQQR LHS--SQP-V NQWVSQIT-- -VRPSESQ--
Anguilla_anguilla_LEPRA     H--SAK--NA VWWL-SYQ-K VPESQYTI-I SDHVSSVTLL NVKPLKQR--
Anguilla_anguilla_LEPRB     L--SAR--NV VWWL-NIQEK VPESLYTI-V NDRVSRVTVP NVRPLKRQ--
Homo_sapiens_GCSFR          SHLDPE-PQI LWRL-GAELQ PGGRQQRLSD GTQESIITLP HLNHTQ----
Danio_rerio_GCSFR           PLTKGKVFYV QWRI-DGQVV PRTYTYQ--E SNMTYSVLIP RLQDTS----
```

```
                                 451
Homo_sapiens_LEPR                FTYDAVYCCN E----HECHH --RYAELYVI DVN-INISCE T--DGYLTKM
Macaca_mulatta_LEPR              FTYDAVYCCN E----HECHH --RYAELYVI DVN-INISCE T--DGHLTKM
Bos_taurus_LEPR                  FTYDAVYCCN E----QECHH --RYAELYVI DVN-INISCE T--DGYLTKM
Sus_scrofa_LEPR                  FTYDAVYCCN E----HECHH --RYAELYVI DVN-INISCE T--DGYLTKM
Rattus_novegicus_LEPR            FTYDAVYCCN E----QACHH --RYAELYVI DVN-INISCE T--DGYLTKM
Gallus_gallus_LEPR               FFYNALYCCH QN---RECHH --RYAELYVV DVN-INIKCE T--DGYLTKM
Ornithorhynchus_anatinus_LEPR    FILKATALCF K----KECPR --QECNLFQI DVN-INISCE T--DGYLTKM
Xenopus_tropicalis_LEPR          FRYDALHCCI NH---NECHH --RYAEIYVL DVN-ISISCE T--DGNQKMM
Anolis_carolinensis_LEPR         FLFNALYCCN EN---KECNH --HYAELYII DVN-ISITCE T--DGNLQKM
Chrysemys_picta_bellii_LEPR      FFYNALYCCN QN---RECHH --RYAELYVV DVN-INITCE T--DGYLTKM
Latimeria_chalumnae_LEPR         FHYDALYCCH RNGKNITCNH --HYAEIHVI DVK-LNISCE T--DGNLTHM
Danio_rerio_LEPR                 -GFDTLMCCL TLGEKSMCSI --AYAKIYTE GRFNANITCE SE-YSYVDTM
Carassius_carassius_LEPR         -GFDTLMCCH QWGERFKCFI --AYAKIYTQ GMFNADITCQ SK-NSEEDTM
Takifugu_rubripes_LEPR           -MYDLLQCTK KR------MI --AYSQVYVE GAS-ISISCE T--NGEIDAM
Orizias_latipes_LEPR             -MYDLLTCTQ KA------AL --PYSQISIE GAS-LDIRCE T--NGNMDTM
Salmo_salar_LEPR                 -LSDTLHCCQ PLGETYSCNY --RYSTIYIK DPV-IDISCV T--NGDLDSM
Oreochromis_mossambicus_LEPR     -MYDLLSCTE GW------SI --PYSQIYVE GAD-IDIKCV T--NGDIDAM
Tachysurus_fulvidraco_LEPR       -GFDTLLCCY PFEQSYKCSI --AYTKVYVE GFFDANITCT TEQHSSVDTM
Epinephelus                      -MYDLLQCTQ EW------TI --PYSQIYVQ GAS-IDITCE T--NGDIDAM
Anguilla_anguilla_LEPRA          -GYDILHCCQ REGEKSLCSF --PYAQIYVK DIS-VAISCE V--DGDLTAM
Anguilla_anguilla_LEPRB          -QYNVLQCCQ RSGETSLCSY --RYASLYTD GVS-VAISCE T--NGDLSAM
Homo_sapiens_GCSFR               ---AFLSCCL NWGNSLQILD QVELRAGYPP AIP-HNLSCL MN-L-TTSSL
Danio_rerio_GCSFR                ---ASILCLV CVQEDCQIVD GVEVKTGNPP -VP-QNLSCA LT-LVKMPSL

                                 501
Homo_sapiens_LEPR                TCRWSTSTIQ SLAESTLQLR YHRSSLYCSD IPSIHPI--- ------SEPK
Macaca_mulatta_LEPR              TCRWSTNTIQ SLAGSTLQLR YRRSSLYCFD IPSIHPI--- ------SKPK
Bos_taurus_LEPR                  TCRWSPNAIQ SLAGSNLQLR YHRSSLYCSD VPSIHPV--- ------SEPK
Sus_scrofa_LEPR                  TCRWSTNAIQ SLVGSTLQLR YHRSSLYCSD VPSVHPI--- ------SEPK
Rattus_novegicus_LEPR            TCRWSPSTIQ SLVGSTVQLR YHRRSLYCPD NPSIRPT--- ------SELK
Gallus_gallus_LEPR               TCRWSANPNA LLLGSSLQLK YHMSKIYCSN FPSTPPE--- ------SEVK
Ornithorhynchus_anatinus_LEPR    TCRWPVDPKT LLTGSTLQLR YYRSSLYCAD FPSAHHA--- ------TDAK
Xenopus_tropicalis_LEPR          TCRWSSQNMT LPEGSVLQFK YYRNKLYCLD KDLKGNV--- ------PISK
Anolis_carolinensis_LEPR         TCRWFTNGDP LLSECTLLLR YYRSDVYCSE SPTISSY--- ------SKIK
Chrysemys_picta_bellii_LEPR      TCRWSANTNT LLVGSSLQLK YYRNSIYCSD FPSIPPK--- ------SEAK
Latimeria_chalumnae_LEPR         TCKWNPNTQN LPIGSSFKLK YYRNNIYCPS SQESQHN--- ------SDFK
Danio_rerio_LEPR                 IYAYG----- ---------- --------CR RQYRSKCT-E EAEEDTSLVK
Carassius_carassius_LEPR         RCEWNKST-- ---WAQVRLL YSSRQHTMCE TISEVEGS-E EAEESMSLVK
Takifugu_rubripes_LEPR           DCRWNSTQ-- ---WLNPNFR TRWADLSCDV MEERERAGDN VG----HEGP
Orizias_latipes_LEPR             ECSWNSTQ-- ---WLSFNLQ HKWTHMSCER MKEKEEAGDN VG----KIVD
Salmo_salar_LEPR                 TCRWNNLP-- ---IGGINFM SRVADLSCDV MEEAERV--- GVPVGVVRQA
Oreochromis_mossambicus_LEPR     DCSWTHKQ-- ---LTKLRFR SKWADLSCDV MEESERAGEN LG----EMGP
Tachysurus_fulvidraco_LEPR       TCKWNKSA-- ---WAVIRFL YRRYRRTCDE IQYEEGTLTQ AQGDPMVDVE
Epinephelus                      TCSWKSTQ-- ---WTRLKFR SRWADLQCDV MEERERAGEK VG----EMGP
Anguilla_anguilla_LEPRA          TCTWNTSQ-- ---WAEVRCL YQRHMLP--- CEEEMEL--- ------PPAV
Anguilla_anguilla_LEPRB          TCRWNISL-- ---G--VRFY YRTSDVPFDI AEEQMAV--- ------SKEE
Homo_sapiens_GCSFR               ICQWEPGPET -HLPTSFTLK SFKSRGNCQT QG-------- ------DSIL
Danio_rerio_GCSFR                RCDWNPVQEI KNLPINYTLH VFRAKSQKV- ---------- ----------

                                 551
Homo_sapiens_LEPR                DCYLQ-SDGF YECIFQPIFL LSGYTMWIRI NHSLGSLDSP PTCVLPDSVV
Macaca_mulatta_LEPR              DCYLQ-SDGF YECVFQPIFL LSGYTMWIRI NHPLGSLDSP PTCVLPDSVV
Bos_taurus_LEPR                  DCHLQ-RDGF YECIFQPIFL LSGYTMWIRI NHTLGSLDSP PACVIPDSVV
Sus_scrofa_LEPR                  DCQLQ-RDGF YECIFQPIFL LSGYTMWIRI NHPLGSLDSP PTCVIPDSVV
Rattus_novegicus_LEPR            NCVLQ-TDGF YECVFQPIFL LSGYTMWIRI NHSLGSLDSP PTCVLPDSVV
Gallus_gallus_LEPR               ECHFQ-RNHS YECTFQPVFL LSGYTMWIEL KHSLGTLESS PTCVVPADVV
Ornithorhynchus_anatinus_LEPR    DCRFQ-DEGF YECTFQPIYL LSGYTMWITI SHPLGTLESP QKCVVPDSLV
Xenopus_tropicalis_LEPR          DCQLQ-MDGF YECTFEPVHL VSGYIMWIEI QHHLGALNSP PVCILPINTV
Anolis_carolinensis_LEPR         ECQPQ-RNNS YECIFQPFYL LSGYTMWIEI KHSLGTVTSQ PVCILPKEVV
Chrysemys_picta_bellii_LEPR      ECHLQ-RNHY YECTFQPIFL LSGYTMWIEI KHQLGKLESP PTCVIPADVV
Latimeria_chalumnae_LEPR         DCHLQ-RNKL YECIFQPISL FSGYIMWVEI YHYLGKLESS PTCVIPMDVV
Danio_rerio_LEPR                 ECPSK-AGDH RQCTLSQISM IFCYKFWLEV EGGRG--QSF PVYVTPIDYV
Carassius_carassius_LEPR         ECPSG-AGDH RECTLRNLSL YSCYKFWLEV EGGHGKVRSF PVYVAPIDYV
Takifugu_rubripes_LEPR           SCLQV-DSRK RLCTIQPLRT -NCYKLWLEV SSHLGLIRSK PVYLTPNDHV
Orizias_latipes_LEPR             ACYSI---KP RTCTFKPLRF -GCYKLWLEL RTDSGSVRSK PIYLSSKGQV
Salmo_salar_LEPR                 KCESSGYRGV KSCNLQPIRV TSCYKLWMEA KTD-NSMRSH PVYITPMDHV
Oreochromis_mossambicus_LEPR     ACME---GGQ ETCTIHPLRM -NCYKLWLEL PSQLGPIRSK PVYLSPVDHV
Tachysurus_fulvidraco_LEPR       ECTAG-AGDY YQCTLQDLSL ISCYKLWLVV EDGYNKVRSL PVFVSPIDCV
Epinephelus                      SCLQV-RSKQ KTCTIQPLRM -NCYKLWLEV PSRLGPIRSK PIYLSPIDHV
Anguilla_anguilla_LEPRA          ECPVL---GA KSCRLEPLFL TSCYIMWVEG REQEGTVKSH PIYILPMDLV
Anguilla_anguilla_LEPRB          ECPSE-GRGL KSCTFQPFLP FSYYMMWLEF GTEEGTVKSQ PVYALPMDLV
Homo_sapiens_GCSFR               DCVPKDGQSH CCIPRKHLLL YQNMGIWVQA ENALGTSMSP QLCLDPMDVV
Danio_rerio_GCSFR                -YAVPPGQHF YVVPRDAYGY FSELEISVTA ANVLGNTTSD PLKLTPLNTV
```

```
                                601
Homo_sapiens_LEPR               KPLPPSSVKA EI-----TIN IGLLKISWEK PVFP---ENN LQFQIRYGLS
Macaca_mulatta_LEPR             KPLPPSSVKA EI-----IKN IGLLKISWEK PVFP---ENN LQFQIRYGLS
Bos_taurus_LEPR                 KPLPPSSVKA EI-----TVK IGLLKISWEK PVFP---ENN LQFQIRYGLS
Sus_scrofa_LEPR                 KPLPPSSVKA EI-----TAK IGLLKISWEK PVFP---ENN LQFQIRYGLS
Rattus_novegicus_LEPR           KPLPPSNVKA EI-----TIN TGLLKVSWEK PVFP---ENN LQFQIRYGLN
Gallus_gallus_LEPR              KPLPPSNIKA EI-----TRN DGLLNVSWTN PVFT---NDD LKFQIRYAVN
Ornithorhynchus_anatinus_LEPR   KPLPPSSIKA EI-----TVY TGLLNVSWER PTFP---ENH LQFQIRYAVR
Xenopus_tropicalis_LEPR         KPLAPSRVRA EM-----TKG SGHLYVSWKR PALP---STD LQFQVRYCLQ
Anolis_carolinensis_LEPR        KPFSPSNVKA EI-----TEE VGLLLVRWNN PEFP---KYD LQFQIRYAAN
Chrysemys_picta_bellii_LEPR     KPFPPSNVKA EI-----TKN VGLLNVSWTN PAFP---NSD LKFQIRYSVN
Latimeria_chalumnae_LEPR        KPLPPTSVEA NI-----TKP DGQLNVTWEI PKLP---EYD LQFQIRYSKN
Danio_rerio_LEPR                KPSPPFDLEA -I-----TLP SKTLSVRWKR PSLP---VYG MQYELQFKAL
Carassius_carassius_LEPR        KPDPPSDLEA -T-----TLP NKTLSVSWRR PNLP---VYD MQYELRFVAL
Takifugu_rubripes_LEPR          KPHTPTDVKA -V-----SRS GGVLNVTWKR PYLP---VE- VQCQFRYHSP
Orizias_latipes_LEPR            KPYTPTNVKA -V-----TLR SGVLSVTWEP PSLP---IDG LQYELQYHPL
Salmo_salar_LEPR                KPHPPSGLEA -V-----SMP SGVLKLAWVP PELP---IYD MQYQVRYALS
Oreochromis_mossambicus_LEPR    KPHAPANVKA -V-----SHS SGVLEVTWQA PPLP---ADG LQCQFQYHSP
Tachysurus_fulvidraco_LEPR      KPSPPSELKA -V-----TLP NKTLSATWKR PYLP---AYD LQYELRYVSM
Epinephelus                     KPHSPTNVKA -V-----SRS SGVLLISWEP PSLP---VEG LQCQFRYHSP
Anguilla_anguilla_LEPRA         KPHPPFDLGA -T-----FLP DAHLSIWWKR PELP---VYE LQFEVRYSVD
Anguilla_anguilla_LEPRB         KPYPPFDLEA -V-----TVP EGYLRATWKR PELP---TYD LLFEVRYAVD
Homo_sapiens_GCSFR              KLEPPMLRTM DPSPEAAPPQ AGCLQLCWEP WQPGLHI--N QKCELRHKPQ
Danio_rerio_GCSFR               KFDPPSITRI EA------HK YGCLKYSWSL SETQKWLQLT FIVQLRLKTV

                                651
Homo_sapiens_LEPR               ---GKEVQWK M-YEVY-DAK SKSVSLPVPD LCAVYAVQVR CKRLDGLGYW
Macaca_mulatta_LEPR             ---GKEIQWK M-YDVY-DAK SKSVSLPVPD FCAVYAVQVR CKRSDGLGLW
Bos_taurus_LEPR                 ---GKQVQWK M-FEVY-DAK LKSASLPVPD LCAVYTVQVR CKSLDGLGYW
Sus_scrofa_LEPR                 ---GKEVQWK I-YEVY-DTK LKSTSLPVPD LCAVYAVQVR CKRLDGLGYW
Rattus_novegicus_LEPR           ---GKEIQWK T-HEVF-DAK SKSASLPVSD LCAVYVVQVR CRRLDGLGYW
Gallus_gallus_LEPR              ---REELTWE L-YEVL-SVP TRSAVIE-VQ LCVEYIVQIR CRALDGLGYW
Ornithorhynchus_anatinus_LEPR   ---GKDGQWK T-YLHSPKAK SESASIEVLD LCMVYVVQVR CKRLDGLGYW
Xenopus_tropicalis_LEPR         ---GQGIIWK V-LDIF---E EEFVSIQVPD VCASYTVQVR SRRTDGVGYW
Anolis_carolinensis_LEPR        ---GTKINWE M-QEIS-TPS VSSAIVIVPD PCTVYIVQVR CSLTDGVGYW
Chrysemys_picta_bellii_LEPR     ---REEILWE I-FEVS-NAP TRSVMIKVLD LCVVYIVQVR CSGLDGLGYW
Latimeria_chalumnae_LEPR        ---GKEKNWK T-QDVV---M TSSGIVEVSN PCTIYIVQVR CTRYEGPGYW
Danio_rerio_LEPR                -AGMANTQWK V-IGPL---L EPQAEIQLEE SCVQFKVEVR CKDVNDTGYW
Carassius_carassius_LEPR        -RDMPNTQWK V-IGPL---L EPQAEVQLAE SCVQFNVEVR CRRLNGSGYW
Takifugu_rubripes_LEPR          SADHPKPDWK V-QAIV---R EPWAEVNVSD VCRVFVVQVR CMHISGAGYW
Orizias_latipes_LEPR            STV--KEEWK V-QRSK---Q PPPMTVQVPE MCRSYVVQVR CMHIAGKGYW
Salmo_salar_LEPR                -TGRAHPFWQ V-LALQ---T ESWAEVLEPD VCGVYNVQVR CRHINGSGTW
Oreochromis_mossambicus_LEPR    STVSPRPKWK L-QDPV---R VPWAEVAVPD MCRVYVVQVR CKHTNGTGYW
Tachysurus_fulvidraco_LEPR      -HGMVDLKWK V-FGSL---L ESRATFTVLD PCIQYQVQVR CRRLNGPGYW
Epinephelus                     SAVRAQPEWK I-QSPV---R VPWAEVLVPD MCRVYVVQVR CMHTSGTGHW
Anguilla_anguilla_LEPRA         ---KDDTLQK V-IRSV---S NQSAVVPIVD PCVVYTIQVR CKRLAGPGFW
Anguilla_anguilla_LEPRB         ---GPDPLWR V-YKSE---V NLTVVFPVSD PCAVYTIMVR CKRLHGSGFW
Homo_sapiens_GCSFR              ---RGEASWA L-VGPL-PLE ALQYELCGLL PATAYTLQIR CIRWPLPGHW
Danio_rerio_GCSFR               ---SNQPNKD LVYTSR-QLQ LNPIEVCSLL HWTDYRSTVR V-KYYATSEW

                                701
Homo_sapiens_LEPR               SNWSNPAYTV VMDIKVPMRG PEFWRIINGD TMKKE--KNV TLLWK-P-LM
Macaca_mulatta_LEPR             SNWSNPAYTV VMDIKVPMRG PEFWRIINGD TMKKE--KNV TLLWK-P-LM
Bos_taurus_LEPR                 SNWSTPAHTV VMDVKVPIRG PEFWRLISED TTKKE--RNV TLLWK-P-LM
Sus_scrofa_LEPR                 SNWSTPAYTV VTDVKVPIRG PEFWRIINED ATKKE--RNI TLLWK-P-LM
Rattus_novegicus_LEPR           SNWSSPAYTL VMDVKVPMRG PEFWRIMDGD ITKKE--RNV TLLWK-P-LM
Gallus_gallus_LEPR              SNWSRSAYAA VKDIQAPLHG PEFWRTVTED PATGQ--KNV TLLWK-P-LM
Ornithorhynchus_anatinus_LEPR   SEWSNPASTL IRDVKAPVRG PEFWRVIKED SGKKA--RNI TLFWK-P-LT
Xenopus_tropicalis_LEPR         SDWSQPVHTV VRDIRVPLQG PTFWRTTHNN PMQKG--DNI SIIWQ-P-LP
Anolis_carolinensis_LEPR        SDWSRPAYTV IKDIKAPLRG PEFWRIVDED PVTNQ--NNV TLFWK-P-LM
Chrysemys_picta_bellii_LEPR     SDWSKPAYTI VQDIQAPLRG PEFWRVINED PIRKQ--KNV TLVWK-P-LM
Latimeria_chalumnae_LEPR        SAWSGPAYTT VYDLKTPEKG PDFWRVINED PLTKV--TNV TLLWQ-P-V-
Danio_rerio_LEPR                SDWSNSHIST VFNLKAPEMG PDFWRILQED PTRNVT--NV TLIFKQP-IL
Carassius_carassius_LEPR        SDWSMSYTSV VYNRKAPEMG PDFWRIIQED PLRNVTNTNV TLIIKQP-IL
Takifugu_rubripes_LEPR          SEWSPSVYSS PQNSRAPERG PNFWRFLQDD PHRKQ--TNV TLLFKDL--Q
Orizias_latipes_LEPR            SEWSDLIYST PNNSKAPERG PDFWRIRQDN QHINK--SNI TLLFEHF--P
Salmo_salar_LEPR                SDWSHLLYTT THNSRAPERG PDFWRVFQED PASTQ--TNV TLLF-EH-SP
Oreochromis_mossambicus_LEPR    SDWSESVYST PQNSRAPERG PDFWRIRQDD PHGNQ--SNI TLLFENF--P
Tachysurus_fulvidraco_LEPR      SDWSYTHASS VYNVKAPEMG PDFWRIIQET PEPY---TNV TLLF-KP-LP
Epinephelus                     SEWSDSVYST PQNSRAPERG PDFWRVLQDD PYRNQ--TNV TLLFEQHHLQ
Anguilla_anguilla_LEPRA         SDWSSPYYTT INNIKAPEQG PDFWRVLQEY PKLNQ--THV TLLF-TL-SQ
Anguilla_anguilla_LEPRB         SEWSDPHYSA VQISRAPERG PDFWRVLKDD RERNQ--SNV TLLF-AP-LT
Homo_sapiens_GCSFR              SDWSPSLELR TTERAPTVRL DTWWRQRQLD PRTVQ----- -LFWK-P-VP
Danio_rerio_GCSFR               SEWSDPKTAT TLNKAPAGRL DTWLKV---N NQTAQ----- -LYWK-P-SQ
```

```
                                 751
Homo_sapiens_LEPR                KNDSLCSVQR YVINHHTSCN GTWSEDVG-- NHTKFTFLWT EQAHTVTVLA
Macaca_mulatta_LEPR              KNESLCSVQR YVINHHTSCN GTWSEDVG-- NHTKFTFLWT EQAHTVTVLA
Bos_taurus_LEPR                  KNDSLCSVRR YVVKHHTSHN GTWLEDVG-- NHTKLTFLWT EQAHSVMVLA
Sus_scrofa_LEPR                  KNDSLCSVRS YVVKHHTSRH GTWSEDVG-- NHTKLTFLWT EQAHSVTVLA
Rattus_novegicus_LEPR            KNDSLCSVRR YVVKHRTAHN GTWSQDVG-- NQTNLTFLWA ESAHTVTVLA
Gallus_gallus_LEPR               KNHSLCSVSR YVIKHQTSEN TSWSEYVD-- NGTTCSFPWT ESTHTITILA
Ornithorhynchus_anatinus_LEPR    RNQSLCSVRS YWVEHHTSNQ VTWTHDAG-- NSTESTFPWT GQDHTVSVVA
Xenopus_tropicalis_LEPR          SEHSLCSIQG YEVIHLNSKN VTWSKYVG-- NTTKHTFTLS DNAVTVTLLA
Anolis_carolinensis_LEPR         KNLSLCSVLG YMVEHTTSDN VTWSDYVE-- NDTTYTFSWA EDVHTIKVIA
Chrysemys_picta_bellii_LEPR      KNYSLCSVCG YIVKHHTSEN ITWTEYVT-- NGTTYTYPWM EDADTITILA
Latimeria_chalumnae_LEPR         --EVLCTVKG FRIQYQTSKN VTWTEHSR-- NETSYTFTWM SSIHTVSILV
Danio_rerio_LEPR                 AGDPNSCVEG LVIKHQASGG VMWSNETT-- LARFHSFQWR KEAHTVTVMS
Carassius_carassius_LEPR         AGDPYSCVEG LVFEHQASGR AVWSNETT-- LVQFHSFQWR KEAHTVTVMS
Takifugu_rubripes_LEPR           TSGQPYCVEG FLVKRLGSTG PV-QEPIL-- MQSSYSFEWN QMPQTVTVEA
Orizias_latipes_LEPR             GTWNSYCVDG FIVQHEASNR SVVRKQIN-- LGSSYSFEWN QEPQTVTVEA
Salmo_salar_LEPR                 IVEPTYCVEE LVVQHQDSGG TVTEERIG-- LVSSYSFEWR KEVHSVTVKA
Oreochromis_mossambicus_LEPR     PSGNSYCVDG FVVQRRSSSG SVLRETIE-- LMSSYSFEWN QELQTVTVEA
Tachysurus_fulvidraco_LEPR       EVEAAICVQG LVVVHQTSGG NVWSDDII-A PSSFYTFQWR EEVHSITVMS
Epinephelus                      LSARSYCIDG FIVQYQALSG SVMREQIE-- LASSFSFEWN QVPQTVTVEA
Anguilla_anguilla_LEPRA          SEGPFCCVEG LTVKHQTSRG SVWSENLG-- RVSTYSFSWT EDVHTVTVLA
Anguilla_anguilla_LEPRB          GEGTLCCVTG IVVQHQTTGG AVWIEQLG-- LVSTYTFPWR EEVHTVTVMA
Homo_sapiens_GCSFR               LEEDSGRIQG YVVSWRPSGQ AGAILPLCNT TELSCTFHLP SEAQEVALVA
Danio_rerio_GCSFR                QFRANGQNLS YSVDSK---- -DTKKRLCVT TETYCFFSLT KWDKKIFLRA

                                 801
Homo_sapiens_LEPR                INSIGASVAN FNLTFSWPMS KVNIVQSLSA YPLNSSCVIV SWILSPSDYK
Macaca_mulatta_LEPR              INSIGASVAN FNLTFSWPMS KVNIVQSLSA YPLNSSCVIL SWILSPSDYK
Bos_taurus_LEPR                  INSIGASSAN FNLTFSRAIS KVNIVQSLSA YPLNSSCVIL SWMLSPSDYN
Sus_scrofa_LEPR                  VNSIGASSAN FNLTFSWPMS KVNIVQSLSA YPLNSSCVGL SWLLSPSDYN
Rattus_novegicus_LEPR            INSIGASLVN FNLTFSWPMS KVNAVQSLSA YPLSSSCVIL SWTLSPNDYS
Gallus_gallus_LEPR               VNSIGASSVN FNLTLSQQMS TVNAVQSLIA YPVNSTCVIL TWTLSPQIYV
Ornithorhynchus_anatinus_LEPR    TNSVGSSSAN FNLTFSRPIS EVDIVQSLGA YPLNSSCVIL SWVLSSHGYN
Xenopus_tropicalis_LEPR          VNSLGYSLTN SKLTFSCEMS TVTSVESFRV YHMNNTCAVA VWTMLPKSDM
Anolis_carolinensis_LEPR         INSIGASSVN FILTLSKQMS TVNIVESLRI YPVNSSCVIV TWTLSPVSYI
Chrysemys_picta_bellii_LEPR      VNSVGASSMN FNLTLSQQMS TVNIVQSLSA YPVNSSCVIL TWTLAPQMYV
Latimeria_chalumnae_LEPR         FNSIGSSTVN YNLTLSKQTS TVQVVQSLHA YLSNSSCVIL SWNLLPGDYL
Danio_rerio_LEPR                 RNALGISTWN RNITLLRQ-A KRRCVRSFSA -VANVSCVHL SWSLLSDQPV
Carassius_carassius_LEPR         RNALGISTRN RNITLFHQ-P KRRVVRSFSV -VANASCVHL SWSLLHDHPV
Takifugu_rubripes_LEPR           FNSLGSSSDN INMTLEKS-P KRRCVHHFSV TVINSTCVSL SWTLIDKSSP
Orizias_latipes_LEPR             YNSLGNSTNN KNMTLGKT-S RRKAVHSVHA LVLNSTHVSL SWSLLNDGIV
Salmo_salar_LEPR                 QNSQGSSTRN THMTLDRH-P KRQCVRLFSA SRVNSSCVVL LWSLQPNSSV
Oreochromis_mossambicus_LEPR     YNSLGNSRDN INMTLERQ-P KGHCVRSFHV LLINGTCVSL SWSLLENSSV
Tachysurus_fulvidraco_LEPR       RNSLGSSAEN SNMTLVRQ-P KRQCVRWFHV -TANASCVFL SWSLLSEQPS
Epinephelus                      YNNLGSSANN FNMTLERQ-P KRRSVRSFSV LVINSTCVSL SWTLLDNNSV
Anguilla_anguilla_LEPRA          HNALGSSTKN SNMTLTRH-T KSQSVHSFSS MMVNSSCVAL SWTLFPNSSA
Anguilla_anguilla_LEPRB          INSLGPSTRN THMTLMRKAS KPRSVSSFSS VMINDSCVAL VWSLFPNSSA
Homo_sapiens_GCSFR               YNSAGTSRPT PVVLSESRGP ALTRLHAM-- -ARDPHSLWV GWE-PPN-PW
Danio_rerio_GCSFR                RNEVGFSDHN EVPVAVHRNK GLEPVSNFSV HPQSNTSLHV IWK-SPAFSN

                                 851
Homo_sapiens_LEPR                LMYFIIEWKN LNED------ --GEIKWLRI SSSV-KKYYI HD--------
Macaca_mulatta_LEPR              LMYFIIEWKN LNED------ --GEIKWLRI SSSV-KKYYI HD--------
Bos_taurus_LEPR                  LMYFILEWKI LNED------ --SEIKWLRI PSSV-KKYYV HD--------
Sus_scrofa_LEPR                  LMYFILEWKI LNED------ --HEIKWLRI PSSV-KKYYI HD--------
Rattus_novegicus_LEPR            LLYLVIEWKN LNDD------ --DGMKWLRI PSNV-NKYYI HD--------
Gallus_gallus_LEPR               ITSFIIEWRN LNKE------ --EEMKWVQV PPNI-SKHYI YD--------
Ornithorhynchus_anatinus_LEPR    LKSLVIEWKN LNED------ --NEMKWLRV STNV-NKYYI HD--------
Xenopus_tropicalis_LEPR          PLEFIVEWKN LGNE------ --EKVQWMNI PRNM-SRCYI ED--------
Anolis_carolinensis_LEPR         ITSFVIEWIN LNGE------ --EQIKWIMV PSDI-RRHHI FD--------
Chrysemys_picta_bellii_LEPR      ITSFVIEWKN LNEE------ --EQMKWIRV APNI-SKYYI YD--------
Latimeria_chalumnae_LEPR         LSSFIIEWKI LNEK------ --ENIKWIRV PSDF-NKFPI KD--------
Danio_rerio_LEPR                 PQSFVIEWLD LNKDPEKDVS LTERIQWVRV ESRS-RDLSL CPRAKHPERK
Carassius_carassius_LEPR         PQSFVIEWLD LNKDPEQDMS LIERLQWVRV QSTA-RDLSL CR--------
Takifugu_rubripes_LEPR           PIFMVVQWSL LWKQDSGRPR GQSTDTWVRL PYTD-GPTYL GG--------
Orizias_latipes_LEPR             PLFMVVQWSE S--------S GLSGLKWARL PYSN-HVVYI KG--------
Salmo_salar_LEPR                 PWSLVVEWSG QNHQDRPDQT SESRERWTRF PPTD-KLLYL YG--------
Oreochromis_mossambicus_LEPR     PLFMVVEWLP HKQQDS---- GPRAETWTRL RYTD-HPVYL RG--------
Tachysurus_fulvidraco_LEPR       LLSFVLEWQE QSGVSSQGWA SDGRVEWLRV ASTA-RDLQL CR--------
Epinephelus                      PLFMVVQWSP HKQQDSDHHK GRIGETWARL PYTD-HPIYL KG--------
Anguilla_anguilla_LEPRA          PSSFVIQWSG QSRSRQ-QDK QGGRVKWVRV PPNN-RAFHL HE--------
Anguilla_anguilla_LEPRB          PASFVVEWSS RSRGRG-RGD AWLRVKWVRV SAPS-RSLYL HD--------
Homo_sapiens_GCSFR               PQGYVIEWGL GPPSASNS-- ---NKTWRME QNGRATGFLL KE--------
Danio_rerio_GCSFR                VTSYVLEWRS LCGTTAAP-- ---L-SFTLI HKN--KSNTT LT--------
```

```
                              901
Homo_sapiens_LEPR             ---------- -----HFIPI EKYQFSLYPI FMEGVGKPKI INSFTQDDIE
Macaca_mulatta_LEPR           ---------- -----HFIPI EKYQFSLYPI FMEGVGKPKI INSFAQDNTE
Bos_taurus_LEPR               ---------- -----YFIPI EKYQFSLYPI FTEGVGKPKI INSFAQDD-E
Sus_scrofa_LEPR               ---------- -----HFIPI EKYQFSLYPI FMEGVGKPKI INSFTQDG-E
Rattus_novegicus_LEPR         ---------- -----NFIPI EKYQFSLYPV FMEGVGKPKI INGFTKDDIA
Gallus_gallus_LEPR            ---------- -----HFILI EKYRFSLYPV FAAGVGKSRA TDQFSKDGYA
Ornithorhynchus_anatinus_LEPR ---------- -----HFIFI EKYQFTLYPV FLEGVGKPKM TNQFIRDENE
Xenopus_tropicalis_LEPR       ---------- -----NFFAI EKYVFSLYPV FPEGVGRSKV VNGFSTVELT
Anolis_carolinensis_LEPR      ---------- -----NFILI DKYRFSLYPI TCEGVMKPYV TDGFSKGEME
Chrysemys_picta_bellii_LEPR   ---------- -----HFILI EKYQFSLYPI SPEGVGNPKT TDEFIKDRSE
Latimeria_chalumnae_LEPR      ---------- -----HFPVF EKYQFTLYPI LSDGVAQPLM IDEFFKGERE
Danio_rerio_LEPR              ESPLVFDNII CFYAGRFYGS EE--FTLYPV FADGEGEPAR YTATRG----
Carassius_carassius_LEPR      ---------- -----RFYGS EE--FTLYPV FVDGEGEPVR YTATRG----
Takifugu_rubripes_LEPR        ---------- -----HFFGS EDYGFYLYPV FAHGEGEPAF ATATRR----
Orizias_latipes_LEPR          ---------- -----SFSRS EDYSFHLYPV FADMEGEPMY IIAAKR----
Salmo_salar_LEPR              ---------- -----HFYDT DEYEFILYPV FADGEGEPVY TKVFRG----
Oreochromis_mossambicus_LEPR  ---------- -----DFFAS EEYGFFLYPV FAEGEGEPIY TLATRG----
Tachysurus_fulvidraco_LEPR    ---------- -----PFYGT EE--FKLYPV FVDGEGEAVR CTAVRS----
Epinephelus                   ---------- -----DFFGS EECGFYLYPV FADGEGEPVY AIASRG----
Anguilla_anguilla_LEPRA       ---------- -----TFFAS EEYQFILYPI FENTEGEPIY AK-DRG----
Anguilla_anguilla_LEPRB       ---------- -----RFYVS EEYQFALHPI FANGEGEPFY NKEDRG----
Homo_sapiens_GCSFR            ---------- -----NIRPF QLYEIIVTPL YQDTMGPSQH VYAYSQEMAP
Danio_rerio_GCSFR             ---------- -----GLEPS KPYEISIYPR YVKGIGRPVT VLAYSSETAP

                              951
Homo_sapiens_LEPR             KH-------- ---------- ---------- ---------- ----------
Macaca_mulatta_LEPR           KH-------- ---------- ---------- ---------- ----------
Bos_taurus_LEPR               KH-------- ---------- ---------- ---------- ----------
Sus_scrofa_LEPR               KH-------- ---------- ---------- ---------- ----------
Rattus_novegicus_LEPR         KQ-------- ---------- ---------- ---------- ----------
Gallus_gallus_LEPR            -S-------- ---------- ---------- ---------- ----------
Ornithorhynchus_anatinus_LEPR KR-------- ---------- ---------- ---------- ----------
Xenopus_tropicalis_LEPR       EA-------- ---------- ---------- ---------- ----------
Anolis_carolinensis_LEPR      -N-------- ---------- ---------- ---------- ----------
Chrysemys_picta_bellii_LEPR   -K-------- ---------- ---------- ---------- ----------
Latimeria_chalumnae_LEPR      K--------- ---------- ---------- ---------- ----------
Danio_rerio_LEPR              ---------- ---------- ---------- ---------- ----------
Carassius_carassius_LEPR      ---------- ---------- ---------- ---------- ----------
Takifugu_rubripes_LEPR        ---------- ---------- ---------- ---------- ----------
Orizias_latipes_LEPR          ---------- ---------- ---------- ---------- ----------
Salmo_salar_LEPR              GD-------- ---------- ---------- ---------- ----------
Oreochromis_mossambicus_LEPR  ---------- ---------- ---------- ---------- ----------
Tachysurus_fulvidraco_LEPR    ---------- ---------- ---------- ---------- ----------
Epinephelus                   ---------- ---------- ---------- ---------- ----------
Anguilla_anguilla_LEPRA       RP-------- ---------- ---------- ---------- ----------
Anguilla_anguilla_LEPRB       RP-------- ---------- ---------- ---------- ----------
Homo_sapiens_GCSFR            SHAPELHLKH IGKTWAQLEW VPEPPELGKS PLTHYTIFWT NAQNQSFSAI
Danio_rerio_GCSFR             SDAPELNYEE ISRSHLKFHW GQIPLEKRNG IIQGYRFYFW HNKNEIKE-I

                             1001
Homo_sapiens_LEPR             ---------- --QSDAGLYV IVPVII---- ---------- ----------
Macaca_mulatta_LEPR           ---------- --QNDAGLYV IVPVII---- ---------- ----------
Bos_taurus_LEPR               ---------- --QHDADLYV IVPIII---- ---------- ----------
Sus_scrofa_LEPR               ---------- --RNDAGLYV IVPIII---- ---------- ----------
Rattus_novegicus_LEPR         ---------- --QNDAGLYV IVPIII---- ---------- ----------
Gallus_gallus_LEPR            ---------- --QTSSNLYM VLPIVI---- ---------- ----------
Ornithorhynchus_anatinus_LEPR ---------- --QSDAGLYV IVPIII---- ---------- ----------
Xenopus_tropicalis_LEPR       ---------- --PKDAGLYV ILPVIS---- ---------- ----------
Anolis_carolinensis_LEPR      ---------- --HNDVNVYV ILPLVI---- ---------- ----------
Chrysemys_picta_bellii_LEPR   ---------- --RNDAGLYV ILPIVI---- ---------- ----------
Latimeria_chalumnae_LEPR      ---------- --IKSDGSYI VLPIII---- ---------- ----------
Danio_rerio_LEPR              ---------- ----DPAAYI LLLIIA---- ---------- ----------
Carassius_carassius_LEPR      ---------- ----GPAAYI LLLIIA---- ---------- ----------
Takifugu_rubripes_LEPR        ---------- ----DPAIYM MLMMIS---- ---------- ----------
Orizias_latipes_LEPR          ---------- ----NPAAYM IIMSIS---- ---------- ----------
Salmo_salar_LEPR              ---------- --A-GPAAYM LLMIIA---- ---------- ----------
Oreochromis_mossambicus_LEPR  ---------- ----DPKAYM MLMIIS---- ---------- ----------
Tachysurus_fulvidraco_LEPR    ---------- ----DPAAYM LLMIIA---- ---------- ----------
Epinephelus                   ---------- ----DPAAYM MLMIIS---- ---------- ----------
Anguilla_anguilla_LEPRA       ---------- --RGDHAAYM LLLIIT---- ---------- ----------
Anguilla_anguilla_LEPRB       ---------- --SAQHAAYA LLLIIA---- ---------- ----------
Homo_sapiens_GCSFR            LNASSRGFVL HGLEPASLYH IHLMAASQAG ATNSTVLTLM TLTPEGSELH
Danio_rerio_GCSFR             -MTTETSVEV KDLQPHTKYH ALLSICTKGG CVNGSFSTLT TERLDGIEMV
```

```
                              1051
Homo_sapiens_LEPR              ------SSSI LLLGTL---- -------LIS HQRMKKLFWE DVPNPKNCSW
Macaca_mulatta_LEPR            ------SSSI LLLGTL---- -------LIL HQRMKKLFWE DVPNPKNCSW
Bos_taurus_LEPR                ------SSSI LLLGIL---- -------SVS HQRMKKLFWE DVPNPKNCSW
Sus_scrofa_LEPR                ------SSSI LLLGTL---- -------LMS HQRMKKLFWE DVPNPKNCSW
Rattus_novegicus_LEPR          ------SSCV LLLGTL---- -------LIS HQRMKKLFWD DVPNPKNCSW
Gallus_gallus_LEPR             ------STSV LLLGAL---- -------LVS HRRMKKLLWE DVPNPKNCSW
Ornithorhynchus_anatinus_LEPR  ------SSSV LLFGTL---- -------LIS HQRMKKVFWE DVPNPKNCSW
Xenopus_tropicalis_LEPR        ------FSVF LLMGTI---- -------LIS HQRMKKLFWK DVPNPKHCSW
Anolis_carolinensis_LEPR       ------SCSV LLFGGF---- -------LIL HQRMKTLFWD DVPNPKNCSW
Chrysemys_picta_bellii_LEPR    ------SSFV LLLGTL---- -------LIS QQRMKKLFWE DVPNPKNCSW
Latimeria_chalumnae_LEPR       ------SSLI LLFGTL---- -------LVS QQRMRKLLWE DVPNPQNCSW
Danio_rerio_LEPR               ------FLSV VLFVTL---- -------MMS QNQMKKLMWK DVPNPNKCSW
Carassius_carassius_LEPR       ------FLSV VLFVTL---- -------LMS QNQMRKLTWK DVPNPNNCSW
Takifugu_rubripes_LEPR         ------FLSI VLLISL---- -------ILS QNQMKKLMWK DVPNPNQCSW
Orizias_latipes_LEPR           ------FLCI L-LLTL---- -------VLT QNQIKRN--- LVPNPKKCSW
Salmo_salar_LEPR               ------FLSI VLFVTL---- -------VIS QNHMKKFMWK DVPNPNNCSW
Oreochromis_mossambicus_LEPR   ------FLFI ILFVTL---- -------VLS QNQMKKFVSK DVPNPRKCSW
Tachysurus_fulvidraco_LEPR     ------FLFV VLFVTL---- -------IIS QNQLKKLMWR DVPNPNNCSW
Epinephelus                    ------FLSI VLFVTL---- -------ILT QNQMKRFVWK DVPNPNKCSW
Anguilla_anguilla_LEPRA        ------FLSV VLFVTL---- -------AVS QNQMRKLVWK DVPNPNNCSW
Anguilla_anguilla_LEPRB        ------FMSV VLFLTL---- -------AAS QRQMMKLVWK DVPNPNNCSW
Homo_sapiens_GCSFR             IIL--G---- -LFGLLLLLT CLCGTAWLCC SPNRKNPLWP SVPDPAHSSL
Danio_rerio_GCSFR              IFVIPACIGA SLLVIIIVFT CFGK------ QERVKMCLWP IIPDPANSSI

                              1101
Homo_sapiens_LEPR              AQGLNFQK-- ---------- ---------- ---------P ETFEHLFIKH
Macaca_mulatta_LEPR            AQGLNFQKIR GFVMLPRLVL NSQAQVIHPP RPPKVLELQP ETFEHLFIKH
Bos_taurus_LEPR                AQGLNFQK-- ---------- ---------- ---------P ETFEHLFIKH
Sus_scrofa_LEPR                AQGLNFQK-- ---------- ---------- ---------P ETFEHLFIKH
Rattus_novegicus_LEPR          AQGLNFQK-- ---------- ---------- ---------P ETFEHLFTKH
Gallus_gallus_LEPR             AQGVDFQQ-- ---------- ---------- ---------P ETFEHLFVKH
Ornithorhynchus_anatinus_LEPR  AQGVNFQK-- ---------- ---------- ---------P ESFEQLFTKP
Xenopus_tropicalis_LEPR        AQGVNFEK-- ---------- ---------- ---------P DTLENLFMKH
Anolis_carolinensis_LEPR       AQGVNFQK-- ---------- ---------- ---------P ETFEHLFLKH
Chrysemys_picta_bellii_LEPR    AQGVNFQK-- ---------- ---------- ---------P ETFEHLFIKH
Latimeria_chalumnae_LEPR       AQGVNFQK-- ---------- ---------- ---------P ETLDDLFVRP
Danio_rerio_LEPR               AKGMDFRQ-- ---------- ---------- ---------I DTMESLFP-H
Carassius_carassius_LEPR       AKGMDFRQ-- ---------- ---------- ---------I DTMENLFP-H
Takifugu_rubripes_LEPR         ARGIDLN--- ---------- ---------- ---------- -AFDHMFH-P
Orizias_latipes_LEPR           AKGIDFQK-- ---------- ---------- ---------V DTFD-LFQ-P
Salmo_salar_LEPR               AQGIDFGK-- ---------- ---------- ---------A DTMEQLFL-H
Oreochromis_mossambicus_LEPR   AKGIDFKK-- ---------- ---------- ---------V DTFDYLFR-P
Tachysurus_fulvidraco_LEPR     AKGIDFKK-- ---------- ---------- ---------L D--GNLFS-H
Epinephelus                    AKGLDLKK-- ---------- ---------- ---------A DNFDHLFQ-P
Anguilla_anguilla_LEPRA        AQGVDFRK-- ---------- ---------- ---------A ETIESLFR-H
Anguilla_anguilla_LEPRB        AQGVDFRK-- ---------- ---------- ---------A EAVENLFR-H
Homo_sapiens_GCSFR             GSWVPTI--- ---------- ---------- ---------- -MEEDAFQLP
Danio_rerio_GCSFR              KRWTTTD--- ---------- ---------- ---------- -SLQGLPAFK

                              1151
Homo_sapiens_LEPR              TASVT-CGPL LL-------- --EPETISED ISVDTSWKNK DEMMPTTVVS
Macaca_mulatta_LEPR            TASVT-CGPL LL-------- --EPETISED ISVDTSWKNK DEMVPTTVVS
Bos_taurus_LEPR                TESTT-FGPL LL-------- --EPETISED ISVDTSWKNK DEMVPATTDA
Sus_scrofa_LEPR                TESVT-FGPL LL-------- --EPETISED ISVDTSWKNK DEMVPPTTVS
Rattus_novegicus_LEPR          AESVI-FGPL LL-------- --EPEPVSEE ISVDTAWKNK DEMVPAAMVS
Gallus_gallus_LEPR             PEAMS-FEPL LL-------- --EPEIVLED ISVTKALEQE DTQDFLVLDS
Ornithorhynchus_anatinus_LEPR  PEAVA-FGPL LL-------- --EPEAVSED VTVDSPWSGE GEQYSVAHAS
Xenopus_tropicalis_LEPR        HKHPANGSPF LF-------- --EPEAVFED LSIDKQVPHE IIDNIPAVTS
Anolis_carolinensis_LEPR       PEALS-FGPL LL-------- --EPEIVLED VTIDKARNNE EKQDLRAIDS
Chrysemys_picta_bellii_LEPR    PEAIS-FGPL LL-------- --EPEIVLED INVAKALKSE DKQDLLAVDS
Latimeria_chalumnae_LEPR       HGKFA-ISPF LP-------- --ATETVSEA FSIEKIIHIE QRKGATAFTS
Danio_rerio_LEPR               SEGLT-ACPL LL-------- --VSESICEV EIIEKPHPLT IENVKDNEEL
Carassius_carassius_LEPR       SEGLT-ACPL LL-------- --VSESICEV EIIEKPHPVT LEHEKDNVVL
Takifugu_rubripes_LEPR         PEGFP-AWPL LL-------- --PPEKISNL VIVDKADLSA LSTP------
Orizias_latipes_LEPR           AEGLQ-ICPL LP-------- --SDNIISKV IIMEKVEKRA F----METQL
Salmo_salar_LEPR               PEGLP-AWPL LL-------- --VSETISQA TIMEKTGPPT SVPDKDLIPA
Oreochromis_mossambicus_LEPR   REGLP-VWPL LM-------- --PSENISQV IIVDKVLT-- --TALIQNPL
Tachysurus_fulvidraco_LEPR     HEGLT-ACPL LP-------- --TSENVCEV EIVEKLFVLE DDQEEKALLH
Epinephelus                    AESLS-AWPL LL-------- --PAENISKV VIVDEVDLSA LTTALIQVPL
Anguilla_anguilla_LEPRA        PERLI-SCPL LL-------- --ESETISEA VIVEKTSPAV QNNERDQVLG
Anguilla_anguilla_LEPRB        PERLT-SCPL LL-------- --EMETISEA VIVEKAHPKA ASEKDRAAWA
Homo_sapiens_GCSFR             GLGTP---PI ---TKLTVLE EDEKKPV--- -----PWE-- --SHNS----
Danio_rerio_GCSFR              EDKDP---VL VYLSHLSLLD MTEKEPFKSG YVKENQWPDD LNIHAD----
```

```
                                1201
Homo_sapiens_LEPR                L-LS--TTDL EKGSVCISDQ FNSVNFS--- ---------- ----------
Macaca_mulatta_LEPR              L-LS--TTDL EKGSVCISDQ FNSVNFS--- ---------- ----------
Bos_taurus_LEPR                  LLLT--TPDL EKGSICISDQ CSSAQFS--- ---------- ----------
Sus_scrofa_LEPR                  LLLT--TPDL EKSSICISDQ RSSAHFS--- ---------- ----------
Rattus_novegicus_LEPR            LLLT--TPDS TRGSICISDQ CNSANFS--- ---------- ----------
Gallus_gallus_LEPR               TFTK--PEDS EHDSACPSSH FSGRSSL--- ---------- ----------
Ornithorhynchus_anatinus_LEPR    LLLLAGNGKA QPGSACSSGP RAGSSFS--- ---------- ----------
Xenopus_tropicalis_LEPR          LFTV--SEEP DHDSACESSN FSSGCAF--- ---------- ----------
Anolis_carolinensis_LEPR         LFAT--IQDL EHDSACSSGH FNSASLS--- ---------- ----------
Chrysemys_picta_bellii_LEPR      MFTT--IQDS EHDSACSSSH FSNSLSE--- ---------- ----------
Latimeria_chalumnae_LEPR         ICRK--NHEI DADSACFSSF FNSDSID--- ---------- ----------
Danio_rerio_LEPR                 --LS--GDKT TTDS---G-- ---------- ------LQGD -SSEA-----
Carassius_carassius_LEPR         TYNS--GDKT TTSS---A-- ---------- ------LLAD -SSEP-----
Takifugu_rubripes_LEPR           ------PDPS VASSVRLHGE PDSPVGQAWP EESHLLPGGD RSSPPNLDYP
Orizias_latipes_LEPR             MSLN--DDSV TSSSACLAPP FERSCLDA-- --------SA PSSQ-SLD--
Salmo_salar_LEPR                 SSP------- -------A-- ---------- ------LCVD -SEVPGL---
Oreochromis_mossambicus_LEPR     ------PDHA DALAGSHSPG FDLNVDQF-- MENETLPVGG PSSAVDLDTL
Tachysurus_fulvidraco_LEPR       RSVD--TEAK SNNL---S-- ---------- ------SIEG -SLDP-----
Epinephelus                      VSLT--PDPD TALSISLPPG YDSEADQAQA TESEVLLSGA PSLAHDTDAL
Anguilla_anguilla_LEPRA          KAGQ--GEMA -SSL---P-- ---------- ------PCGN -SEEPPV---
Anguilla_anguilla_LEPRB          EKAL--HAAV -TP------- ---------- ---------- ----------
Homo_sapiens_GCSFR               ---------- -------S-- ---ETCGLPT LVQTYVLQGD ----------
Danio_rerio_GCSFR                ---------- -------T-- ---QSCDLEL E--------- ----------

                                1251
Homo_sapiens_LEPR                ---------- ---------- EAEGTEVTYE AESQRQPFVK YATLISNSK-
Macaca_mulatta_LEPR              ---------- ---------- EAEGTEVTCE DESQRQPFVK YATLISNSK-
Bos_taurus_LEPR                  ---------- ---------- EAESTDITCE DESRRQPSVK YATLLSNSK-
Sus_scrofa_LEPR                  ---------- ---------- EAESMEITRE DENRRQPSIK YATLLSSPK-
Rattus_novegicus_LEPR            ---------- ---------- GAQSTQGTCE DECQSQPSVK YATLVSNVK-
Gallus_gallus_LEPR               ---------- ---------- ECSP-SDPTS GETASQSNIK YATVITNSR-
Ornithorhynchus_anatinus_LEPR    ---------- ---------- EGRGP-GSPW RAARRPSNVK YATLVNNSK-
Xenopus_tropicalis_LEPR          ---------- ---------- ETDH--QEMV YSSICQSSIE YATIMNNTQ-
Anolis_carolinensis_LEPR         ---------- ---------- ESVC-DVETS RGMTGQSNVK YATIITNSM-
Chrysemys_picta_bellii_LEPR      ---------- ---------- -SSH-DDKVS EGITRQSNIK YATIISNLK-
Latimeria_chalumnae_LEPR         ---------- ---------- SARN--EKAY PESTGQSNIK YATILNISE-
Danio_rerio_LEPR                 ---------- ---------- -LEASTAAPT PETSGQSSVT YSTILLSDQ-
Carassius_carassius_LEPR         ---------- ---------L SLETSTAAPT PETSGQSSVT YSTILLSDQ-
Takifugu_rubripes_LEPR           TGSAPDDG-- ---------- ----SCPAGV TDSSAQSSVI YTAVLLCGPK
Orizias_latipes_LEPR             -------E-- ---------- -ANQADPIVP VDSSTSSSVR YAKLLLPCLK
Salmo_salar_LEPR                 ----PE---- -------EEE TLQLPDLPRS LESSAQPSVT YATVLLSDD-
Oreochromis_mossambicus_LEPR     TSSSSRADLQ PADPSVNQHP GST------- -ENSGQSSVT YTAVLVSNPS
Tachysurus_fulvidraco_LEPR       ---------- ---------L SLDTSTVSAS PDTSGQSSVR YSTILVFDQ-
Epinephelus                      TSSSPTTDQL QLIHPLAEQP GSTDSSAQSS AQNSAQSSVT YATVLLPDM-
Anguilla_anguilla_LEPRA          ---------- ---------- -QETSLSPGS PDDSAQSRIS YAMVLFPGT-
Anguilla_anguilla_LEPRB          ---------- ---------- -------FSS AGSSAQSSVA YATVLPAAE-
Homo_sapiens_GCSFR               ---------- ---------- ------PRAV STQPQSQSGT SDQVLYGQLL
Danio_rerio_GCSFR                ---------- ---------- ---------- -----RESVP YATVLFSTPY

                                1301
Homo_sapiens_LEPR                ----PSE-TG E-EQGLINSS VTKCFSSKNS ---PLKDSFS NS-------S
Macaca_mulatta_LEPR              ----PSE-TD E-EQGLINSS VTKCFSSKNS ---PLKDSFS NS-------S
Bos_taurus_LEPR                  ----SGE-TE E-EQGLINSS VSKCFLSNNS ---PPKDSSS KR-------S
Sus_scrofa_LEPR                  ----SGE-TE Q-EQELVSSL VSRCFSSSNS ---LPKESFS NS-------S
Rattus_novegicus_LEPR            ----TVE-TD E-EQGAIHSS VSQCIARKHS ---PLRQSFS SN-------S
Gallus_gallus_LEPR               ----SGG-LY E-QNKNPRCH FDGCFLAEDS ---LAAGACS GS-------S
Ornithorhynchus_anatinus_LEPR    ----SGG-LD E-QDRGASGS LGRGFSSKSS ---LLRGAQD GE-------S
Xenopus_tropicalis_LEPR          ----QCR-KY SSERKTSLSS FDGCLLGNSS ---MVIGN-- ----------
Anolis_carolinensis_LEPR         ----SGG-LY E-PPKDLSSS LDRGFIGHHS ---LASASFS SS-------S
Chrysemys_picta_bellii_LEPR      ----SSG-LY E-EQKNLSGS FNGCFLGEDS ---LVTDPF- SR-------S
Latimeria_chalumnae_LEPR         ----SSE-LC E-QQKGLSST HAFGNIP--- ---SSDSDIS CN-------Q
Danio_rerio_LEPR                 ----PSQ-LQ KQQESLSSSS DEGNFSANNS ---DISGSFP GG-------L
Carassius_carassius_LEPR         ----PSL-LR KQQESLSSSS DEGNFSANNS ---DISGSFP GG-------L
Takifugu_rubripes_LEPR           -QQQQHHHLH DKDCSCSSSS DEGNFSANNS ---DISASFN GG-------L
Orizias_latipes_LEPR             QEKQP--GNP DKDGSGSNSS DEGNFSANNS ---EISESSP TG-------L
Salmo_salar_LEPR                 ----PHH-LY KQEGSLSSSS DEGNFSGNNS ---DISGSFP GG-------L
Oreochromis_mossambicus_LEPR     QDQQPPIHRP YKDGSGNSSS DEGNFSANNS ---DISGSFP GG-------L
Tachysurus_fulvidraco_LEPR       ----PVL-QR KQQESLSSSS DEGNFSANNS ---DISGSFP GG-------L
Epinephelus                      KQEQPPIHLH YKDGSGSSSS DEGNFSANNS ---DISGSFP GG-------L
Anguilla_anguilla_LEPRA          ----PGL-LY KQQESLSSSS DEGNFSANNS ---DISGSFP GG-------L
Anguilla_anguilla_LEPRB          ----GGRARR RPQESLSSCS DEGNFSADTS ---DMSGSYP GA-------L
Homo_sapiens_GCSFR               GSP------- -------TSP GPGHYLRCDS TQPLLAGLTP SP--KSYENL
Danio_rerio_GCSFR                -QI------- -------NSS NPPAYVRSES TQPLLAGDEP GSPPPPYENV
```

```
                              1351
Homo_sapiens_LEPR              WEIEAQAFF- ILSDQHPNII SPHL----TF SEGLDELLKL EGNFPEENND
Macaca_mulatta_LEPR            WEIEAQAFF- ILSDQRPNII LPHL----TF SEGLDELLRL EGNFPEENND
Bos_taurus_LEPR                WEIETQAFF- ILSDQHPNII SPHL----PF SEGLDELLKL EGNFPEENNN
Sus_scrofa_LEPR                WEIETQAFF- ILSDQHPNMT SPHL----SF SEGLDELMKF EGNFPKEHND
Rattus_novegicus_LEPR          WEIEAQAFF- LLSDHPPNVI SPQL----SF S-GLDELLEL EGNFPEENHG
Gallus_gallus_LEPR             WELGNEAFL- LLPDQPGSQP CKT--LSLIS SEGFSEPSDQ DDAFTDGGSP
Ornithorhynchus_anatinus_LEPR  EA-------- -----RPP-- -----YPLPS SSGLPEAVAQ GVRRPRDGGP
Xenopus_tropicalis_LEPR        HDVDKQTLV- FLAGLHTKQP DKMSCNSTVS SEGFSEPLDH EDSFLEADGL
Anolis_carolinensis_LEPR       WAMGNQGFV- ILPECHQTLP RKSLSLS-VS SEGFSELSEQ DKAFTGEDSL
Chrysemys_picta_bellii_LEPR    WEVGNQAFL- ILPDQHPSQA SKTISLSVVS SEGFSEPSDH DDTFSDGDSP
Latimeria_chalumnae_LEPR       WESEKQITL- QLEEIKPIGI NKITFCSSNS SEGFSEPSNE EENDLEEYSP
Danio_rerio_LEPR               WDLEN----- ---------S NPRHSSSYNS VEEFSETSEP DYEASENTGL
Carassius_carassius_LEPR       WDLENHVC-- ------SDSA NPRHSSSYNS VEEFSDTSDQ DYEASENTGV
Takifugu_rubripes_LEPR         WELD------ ---------- VPRRSCCYNS TEELSEKPEQ GD--RDVG--
Orizias_latipes_LEPR           WELDSCHS-- ------AEMD DQRRFCSYAS EGELSEISEH EA-VMEQR--
Salmo_salar_LEPR               WELEISHS-- ---GTGESDL DPRHSCSYNS VEEFSETSEQ EDEALGGERD
Oreochromis_mossambicus_LEPR   WELESCRG-- ------LEMD DPRRSCSYNS VEELSQNSDQ EE-EHEVR--
Tachysurus_fulvidraco_LEPR     CELERQSS-- ------SDAI NPRNSCSYNS VEEFSETSEQ EDEASESIQV
Epinephelus                    WELDSCRG-- ------GEID DPRRSCSYNS VEELSETSEQ EDEEEEAR--
Anguilla_anguilla_LEPRA        WELENPP--- ------SNDS DPRHSCCYNS AEEFSDTSDQ EDEVLDGTGA
Anguilla_anguilla_LEPRB        WEPEGMP--- ------SNPR HPCHSCA--S TEEFSENSDQ EDQSLDGTGS
Homo_sapiens_GCSFR             WFQAS--PL- --GTLVTPAP SQEDDCVFGP LLNFPLLQGI RVHGMEALGS
Danio_rerio_GCSFR              PRGGAVSALN RFSAFSQSTQ SEESDEL--- WEEFPMLRSL EVNHI-----

                              1401
Homo_sapiens_LEPR              -------KKS IYYLGVTSIK KRESGVLLTD K--------- ---------S
Macaca_mulatta_LEPR            -------EKS IYYLGVTSIK KRESGVLLTD K--------- ---------S
Bos_taurus_LEPR                -------ERP VYYLGVTSIK KRESDVFLTN E--------- ---------S
Sus_scrofa_LEPR                -------ERS VYYLGVTSIK KRESDVFLTD E--------- ---------S
Rattus_novegicus_LEPR          -------EKS VYYLGVSSGN KRENDMLLTD E--------- ---------A
Gallus_gallus_LEPR             -------ERG LHYLGITSLG KRENDIFLTE S--------- ---------S
Ornithorhynchus_anatinus_LEPR  -------GQP LYYPSLPLGR SGQNEALVAE S--------- ---------P
Xenopus_tropicalis_LEPR        -------ERN LYYLEFGSIQ QCGQQDCYSE K--------- ---------P
Anolis_carolinensis_LEPR       -------EGG MFYLGMSPFE RQETDLFLTE S--------- ---------S
Chrysemys_picta_bellii_LEPR    -------ERS LYYLGLTSIK KNENDIFLTE N--------- ---------S
Latimeria_chalumnae_LEPR       -------ERN LCYLGLTSNE EEEKESLMKA D--------- ---------S
Danio_rerio_LEPR               -------AKD LYYLEMTGEE KEEEEEEEEE EEEEDEPEEG QSK------N
Carassius_carassius_LEPR       -------AKD LYYLEVNEEE DEDEEEI--- ----KETQGE QEK------N
Takifugu_rubripes_LEPR         ------EEKD LYYIGADYGD EDEESEEEL- -------NAK LI--QTVPLN
Orizias_latipes_LEPR           ------REQT LCYLQIGYPD EDEESAEEVQ REEEEKRKEQ PA--KDASLN
Salmo_salar_LEPR               GGIEVIEEKD LYYLGMGYQE ESEGEEEE-- EEKEEEDTGA MLLKEVMVLG
Oreochromis_mossambicus_LEPR   ------REKD LYYLGMDYPA EDEESEKEDG QSEDEEAKVE LL--KSTPLN
Tachysurus_fulvidraco_LEPR     -------SKE LYYIGMNEEE EEDELEEKFK EDDATK---- ----------
Epinephelus                    ------EEKD LYYLGMDYPA EDEESEEDKE QIE----DIE LL--KNVVLS
Anguilla_anguilla_LEPRA        -------GKE LYYLGMTSQS EEEEDEKQGE DEEEEEEVGA RYP----GED
Anguilla_anguilla_LEPRB        -------RQD LYYLGTISQS EEGEEG---- ---------K PFH----SES
Homo_sapiens_GCSFR             ---------- -------F-- ---------- ---------- ----------
Danio_rerio_GCSFR              ---------- ---------- ---------- ---------- ----------

                              1451
Homo_sapiens_LEPR              RVSCPFPAPC LFTDIRVLQD SCSHFVENNI NLG-----TS SKKTFASYMP
Macaca_mulatta_LEPR            RVLCPFPAPC LFTDIRVLQD SCSHFVENNF NLG-----TS SKKTFASYMP
Bos_taurus_LEPR                RVLSPFPAHC LFTDIRILQD SCSHLVENNF NLG-----TS GQKTFVSYMP
Sus_scrofa_LEPR                RVRCPFPAHC LFADIKILQE SCSHLVENNF NLG-----TS GQKTFVSYMP
Rattus_novegicus_LEPR          GVLCPFPAHC LFSDIRILQE SCSHFVENNL NLG-----TS GK-NFVPYMP
Gallus_gallus_LEPR             RLMCHFHTAD LLRGVGFLQN TPPNL--NAF IQS-S----- -IKAIVPYVP
Ornithorhynchus_anatinus_LEPR  G-LTPGPSAA DPGGGGAPRD AGSEF--NPF VEQDPQMGAP GKRTFVSYMP
Xenopus_tropicalis_LEPR        LGTFPFQENI SYKEIDFKKD KASEFIDNYD IK------NS FKKAFLCYMP
Anolis_carolinensis_LEPR       NMACQFHTSA LIGSIRFPQN IASNLNLNPF IS----RHET SVQTFISYMP
Chrysemys_picta_bellii_LEPR    RVMCHLHTNG LFKDMGFLQD ISSDL--NPF IKN-SLKYEN SVKTFVPYMP
Latimeria_chalumnae_LEPR       NISCQFQTNG QFNTTEYPED NTLSNFDSIF KVS-LNIKLS PKKNMRSYMP
Danio_rerio_LEPR               KRVMGVNPRP LLE------S QNSTAS-N-- -------SNN MSHSIPLYLP
Carassius_carassius_LEPR       EIVVRVDARP LLE------S KDSTSV-D-- -------SNN ISHSIPLYLP
Takifugu_rubripes_LEPR         SEGCSAESRR LLE------- ---------- L------TES KCDFSPLYLP
Orizias_latipes_LEPR           GK-------- ---------- --------DF F------VPL ICDLSSQYMP
Salmo_salar_LEPR               REGSSVESIP LLG------S QDSMFS-EYS DEG----LVV GMRSVPLYLP
Oreochromis_mossambicus_LEPR   RGHCSLELHP LLG------Q DNPSEP-GIL P------SPS TCGFAMPYLP
Tachysurus_fulvidraco_LEPR     ---------- ---------- ----EQ-I-- -------SDS GDSNVPLYLP
Epinephelus                    REDCSVELHP LLS------P EDSSE----L L------LAS TRGFSSLYLP
Anguilla_anguilla_LEPRA        AQELPLESSP LLG------R WDPRLD-R-- -------TDA AAKGPPLYVP
Anguilla_anguilla_LEPRB        TLGRCLESSP LLG------Q QEPWSH-G-- -------EER SGKGVPLYMP
Homo_sapiens_GCSFR             ---------- ---------- ---------- ---------- ----------
Danio_rerio_GCSFR              ---------- ---------- ---------- ---------- ----------
```

```
                                1501
Homo_sapiens_LEPR               QFQTCSTQTH KIMENK---M CDLTV
Macaca_mulatta_LEPR             QFQTCSTQTH KIMENK---M CDLTV
Bos_taurus_LEPR                 QFQTCSTQTQ KIMENK---M CDLTV
Sus_scrofa_LEPR                 QFQTCSTQTQ KIMENK---M YDLTV
Rattus_novegicus_LEPR           QFQSCSTHSH KIIENK---M CDLTV
Gallus_gallus_LEPR              QFQMTAAKVQ ETTENS---C -----
Ornithorhynchus_anatinus_LEPR   QFQRSTVRAQ EAAESK---T CHLSA
Xenopus_tropicalis_LEPR         QFQTHSIKLP GEMESE---T LN---
Anolis_carolinensis_LEPR        QFQPLAIKLH EKAGGK---A -----
Chrysemys_picta_bellii_LEPR     QFQATTIKVQ ETTETK---T -----
Latimeria_chalumnae_LEPR        QFQIKAAKI- ---------- -----
Danio_rerio_LEPR                QFRSECINPT ---------- -----
Carassius_carassius_LEPR        QFRTECINPP ---------- -----
Takifugu_rubripes_LEPR          QFRTAPSCTR QLSAKPQEGR CHP--
Orizias_latipes_LEPR            QYRTAA-YRS QLV------- -----
Salmo_salar_LEPR                QFRTVPSSPL KAQDSAHQL- -----
Oreochromis_mossambicus_LEPR    QFRTAT-CTA QHTQREP--- -QL--
Tachysurus_fulvidraco_LEPR      QFQTAAIKLL REPAGNSTIQ ----L
Epinephelus                     QFRTAP-YTR QLTAQPHDGK PQQ--
Anguilla_anguilla_LEPRA         QFRTASRKSQ QAKAKERSGV ESLQV
Anguilla_anguilla_LEPRB         QFQTVVTESQ STKGL----- -----
Homo_sapiens_GCSFR              ---------- ---------- -----
Danio_rerio_GCSFR               ---------- ---------- -----
```
