# Supplementary material for: Duplicated Leptin Receptors in Two Species of Eel Bring New Insights into the Evolution of the Leptin System in Vertebrates
Source: PLoS One. 2015 May 6;10(5):e0126008. doi: 10.1371/journal.pone.0126008 (PMC4422726; doi:10.1371/journal.pone.0126008)
Supplement: S10 Fig — The sequences were aligned by Clustal Omega and manually adjusted. The amino acids presenting similar physico-chemical properties are shaded in the same color. (PDF) [file pone.0126008.s010.pdf]

```
                                   1
Homo_sapiens_LEPR                  ---------- -----ICYVE SLFKNLFRNY NYKV--HLLY VLPEV-LEDS
Macaca_mulatta_LEPR                NWNIQCWLKG DLKLFICYVE SLFKNPFKNY KHKV--HLLY VLPEV-LEDS
Bos_taurus_LEPR                    NWNIQCWMKE DLKLFICNIE SLFKNPFKNY DLKV--HLLY VLLDV-LEES
Sus_scrofa_LEPR                    NWNIQCWMKE DLKLFICYME SLFKNPFKNY DLKV--HLLY VLLEV-LEGS
Rattus_novegicus_LEPR              NWDIECWMKG DLTLFICHME PLLKNPFKNY DSKV--HLLY DLPEV-IDDL
Gallus_gallus_LEPR                 NWNIECWVEG KLDLLVCSLQ FPKFH--VRL DMKV--HLLY AVSELSLGDA
Ornithorhynchus_anatinus_LEPR      SWNILCWTKG DLKTFICHLT TFSKISFKSY DFRL--RLLY VMSELPPEDS
Xenopus_tropicalis_LEPR            SWKVQCSFEE KANTLICDLQ LLPETKHIVT DYRI--SLHY SLVAK-----
Anolis_carolinensis_LEPR           LWNLQFWTQE STQELLCIVE LSAKSPYWNE DLTV--NLHY ALTDVSLEAT
Chrysemys_picta_bellii_LEPR        SWNIQCWTKG DLEQFVCILK LSNKKSYLNG EFKI--NLLY VLSELSLEDM
Latimeria_chalumnae_LEPR           NWITLCWTDG NLEHLVCHLK PTYKKTYFSG ICRT--KYIH NLSELTVEEL
Danio_rerio_LEPR                   CLDILCWLEG ERENLICNAK TRRAAAAAAS TLVSVSP-HQ LVV--QM-D-
Carassius_carassius_LEPR           CLDILCWLEG ERTNLICNAK SHRAAAATS- -LLTI-R-PQ LVV--QM-N-
Takifugu_rubripes_LEPR             CLDILCGIDE KWENVTCHLE PHALPLSLPD AGHMAVSLQR RFQKSQS-R-
Orizias_latipes_LEPR               CLEILCQINE KWENLTCYLQ P----SRKLD TGGMTFSFQQ LPDKD-----
Salmo_salar_LEPR                   CWDILCRVDE TWDNVICDLK HPATSSDTST PGSVALSLQH LATLPD----
Oreochromis_mossambicus_LEPR       CLDILCRIDG NWQNLICDLR SRGQPSDS-- --LMAVSLQR QLFQEDG---
Tachysurus_fulvidraco_LEPR         RLDIFCRLED EQANVICLLK HQRTSATDAS HLIVSL--WR VTL--ES-D-
Epinephelus_coioides_LEPR          CLDILCRIDE NWENLTCDLQ SHSQPSTTLD AGLMAVSLQR LLSQKDA-E-
Anguilla_anguilla_LEPRA            CLNILCWVDG NLENVICNLK LYVGLSDTSR PIAI--SLQR LPSQFGS-D-
Anguilla_anguilla_LEPRB            CLDVLCWVTG DRRHLVCERN PRGGNVAVGG VVTL--SLLR LQSDQQM-QA
Lepisosteus_oculatus               CLNILCWVDG NLEHLICNLK PHTKYPDRSR FLTI--RLRY SFVSSKQ-K-
Homo_sapiens_GCSFR                 ---------- ---------- ---------- ---------- ----------
Danio_rerio_GCSFR                  ---------- ---------- ---------- ---------- ----------

                                  51
Homo_sapiens_LEPR                  PLVPQKGSFQ M-VHCNCSVH ECCECLVPVP TAKLNDTLLM -CLKITSGGV
Macaca_mulatta_LEPR                PLVPQKGSFQ M-VHCNCSVH ERCECLVPVP TAKLNDTLLM -CLKITSGGV
Bos_taurus_LEPR                    PLLPQKDSFQ V-VQCNCSVH ECCECHVPVP TAKLNDTLLM -YLKITSGGA
Sus_scrofa_LEPR                    PLLPQKGSFQ S-VQCNCSAR ECCECHVPVS AAKLNYTLLM -YLKITSGGA
Rattus_novegicus_LEPR              PLPPLKDSFQ T-VQCNCSVR E-CECHVPVP RAKVNYALLM -YLEITSAGV
Gallus_gallus_LEPR                 STSSLKRTAL A-AQCNCSEY GKCECHVPS- -PRLNHTYVM -WLKTVIGVT
Ornithorhynchus_anatinus_LEPR      SMMPQKDSFK V-IPCNCRVP EGCECEIPLP ATKLNCTHIL -YLEILNEVT
Xenopus_tropicalis_LEPR            ------SELK GTAECRCFGY EKCECIVPS- -VKFNDTYIL -WIEILNITA
Anolis_carolinensis_LEPR           STHSLKDNFT V-THCNSIRD QKYACQISS- -VKLNHTYIM -WLKITNGIA
Chrysemys_picta_bellii_LEPR        STGSLKGNFM V-TPCNSNGS DKHECHIPS- -LKLNYTYIT -WLNIINGVT
Latimeria_chalumnae_LEPR           PVKYQKKSLV VVNQQSCLGH DNFECIIPS- -VKLNYTYFM -WMEITTNAG
Danio_rerio_LEPR                   -VHSDET--- -NSTAQCVGE ETAICSVSL- -HGGDATVSL -VIIISENGT
Carassius_carassius_LEPR           -VRSDEIMST GSHAAQCAGE ETVMCSVSL- -HGNDATVLL -TISITLNET
Takifugu_rubripes_LEPR             ---VDSEEAA SDPPVFCEAE DSFTCSVAL- -DAESSFHAV VTVTIAD---
Orizias_latipes_LEPR               ------GTEV NSNPVVCEAE ESFTCSLPL- -HPAASFVTT VTVNLSS---
Salmo_salar_LEPR                   ------SEVN TTHGTDCVGE DSITCSIAL- -HVVSSIVVV -TANVSNT--
Oreochromis_mossambicus_LEPR       ------DYPA SENPVVCEAQ DSFMCSLTL- -DPTTSFVAM VTVSISD---
Tachysurus_fulvidraco_LEPR         -PLTN--ETN ITTQVQCPGE DEITCFFVL- -QPNDVSVSL -SVSGFLGGR
Epinephelus_coioides_LEPR          ---VDDGNAA TDNPVVCEAK DSFMCSVAL- -DTKTSFVTR VTVSISD---
Anguilla_anguilla_LEPRA            ------NVTD SGAHSKCEGQ DVRTCSVAL- -HSVNSTVSL -MISISDGSS
Anguilla_anguilla_LEPRB            TPSALPDGVT HSCQEEGEGV GVFWCALPP- -GSMRDAVTL -RVNVARGNR
Lepisosteus_oculatus               ------EEIV YAAESSCEGE DTLRCAIPL- -KSLNSTYTL -RVTISDGKS
Homo_sapiens_GCSFR                 ---------- ---------- ---------- ---------- ----------
Danio_rerio_GCSFR                  ---------- ---------- ---------- ---------- ----------
```

```
                              101
Homo_sapiens_LEPR             IFQSPLMSVQ PINMVKPDPP LGLHMEITDD GNLKISWSSP PLVPF--PLQ
Macaca_mulatta_LEPR           IFQSPLMSVQ PINMVKPDPP LGLRMEITDD GNLKISWSSP PLVPF--PLQ
Bos_taurus_LEPR               VFHSPPMSAQ PINVVKPDPP LGLRMEITDT GSLKISWSSP TLVPF--QLQ
Sus_scrofa_LEPR               VFHSPLMSVQ PINVVKPDPP LGLHMEITDT GNLKISWSSP TLVPF--QLQ
Rattus_novegicus_LEPR         SFQSPLMSLQ PMLVVKPDPP LGLRMEVTDD GNLKISWDSQ TKAPF--PLQ
Gallus_gallus_LEPR            PLWSPLMSVK PIDIVKPEPP LNVRLEMTER GQVKICWSEP VPMPY--PLR
Ornithorhynchus_anatinus_LEPR SLQSPIMATQ PINVVKPDPP IRLQMEMVEY RQLKVSWLSP PLSPY--PLR
Xenopus_tropicalis_LEPR       LLHSPPMSVV PYHIVKPDPP DDLRAEIMEQ GTLKVFWLKP ISAAY--ELQ
Anolis_carolinensis_LEPR      LLQSPLMSVK PIDIVKPEPP LHLKMEMTDK GQLKISWSSP ASKSY--PLQ
Chrysemys_picta_bellii_LEPR   LLQSPLMSVR PINIVKPEPP LNLRLEMTDK GQLKICWSNP VLTPY--PLQ
Latimeria_chalumnae_LEPR      MLQSPLMSIM PIDIVKPNPP LNLQGDITNE GLLRINWTTP DPLPY--ELQ
Danio_rerio_LEPR              TAQSQKMQVS TYELQAGDPS RELKPSPLSM KSPVFKHFG- FPVSY--VDN
Carassius_carassius_LEPR      TALSPKMQVS THHLRRPDAP VNLHYNVTTE GEVIFRWSDT QPDSY--AVN
Takifugu_rubripes_LEPR        ARAPSVLLRV PARPVKPAPP VNLSHVQTIE AELILHWGDP KDIK-TDLLQ
Orizias_latipes_LEPR          VVAPPVLLII PARPVKPSPP VNVTHYQTIE AELFVQWESP PHFD-AAQLR
Salmo_salar_LEPR              TAGPLVMLSV PPRLWKPSPP LNLTHTQTTE GELILSWSDP QPHASPVQLS
Oreochromis_mossambicus_LEPR  AVAPPVLLRV PARPEKPSPP GNLSHIQTIE AELIVLWDDP ADFD-AGPLR
Tachysurus_fulvidraco_LEPR    PLQTPEMRIS TDLLRKPEAP FYLRYNVTTE GEVMIAWNDS QNNKL--PLY
Epinephelus_coioides_LEPR     AVAPPVLLRI PARPVKPSPP VNLLHNQTIE ADLILQWDDP SDSD-TGPLR
Anguilla_anguilla_LEPRA       SVQSPVMDIV PLSFLKPDPP LNLQYHMTIE GELRLSWTHA LPSAE--RFV
Anguilla_anguilla_LEPRB       SALSPEISFV PQKLVRPDPP VKLWYNMTTE GELRLHWTPP QPVTG--PLT
Lepisosteus_oculatus          AMESPAMHIV PGNVVKPDPP LKLEYHMTLE GELWLNWTEP QPAPH--KLK
Homo_sapiens_GCSFR            ---------- ---------- ---------- ---------- ----------
Danio_rerio_GCSFR             ---------- ---------- ---------- ---------- ----------

                              151
Homo_sapiens_LEPR             YQVKYSEN-- STTVIREADK IVSATSLLVD SILPGSSYEV QVRGKRLD--
Macaca_mulatta_LEPR           YEVKYSEN-- STTVIREADK IVSATSLLVD GILPGSSYEV QVRGKRLD--
Bos_taurus_LEPR               YQVQYSEN-- STKYIRKTDE IVSATSLLVD SVLPGSSYGA QVRCKRLD--
Sus_scrofa_LEPR               YQVKYSEN-- STTNMREADE IVSDTSLLVD SVLPGSSYEV QVRGKRLD--
Rattus_novegicus_LEPR         YPVKYLEN-- ST-IVREAAE IVSDTSLLVD SVLPGSSYEV QVRSKRLD--
Gallus_gallus_LEPR            CEVNISGN-- SDQNDWQVVQ VALNTSLDID NMLLDSSSFA QVRCKSHC--
Ornithorhynchus_anatinus_LEPR YQVKYSVN-- TTKTAKQVAE VVSATSLLVD DVLPGSSYAV QVRAKRLQ--
Xenopus_tropicalis_LEPR       YQVRYTVK-- AAETNSQVYL LVNETSVIIS DIQPCTEMVI EVRCINLH--
Anolis_carolinensis_LEPR      YEIKCFAN-- STKNVWQVVQ ITLETSLIIN NALFDSSYNI QVRCKRHY--
Chrysemys_picta_bellii_LEPR   YEVKFSGN-- ATQNAWQVVE IVIETSLIIG NMLAGSSYLV QVRCKSLH--
Latimeria_chalumnae_LEPR      YEVKYSIN-- STDSTWQIVK VVMDTSLQLG SVQLGFLYLA QVRCKRLL--
Danio_rerio_LEPR              VRVVDKKKT- QFCKLCYVLK VEGRSWVALN ELSSDIRYTV QVRCQN----
Carassius_carassius_LEPR      YEIRYS-SN- SSLQQWEMVK VKGRSWVPLN DLSSAIRYTV QVRCQS----
Takifugu_rubripes_LEPR        YEVRYSPD-- TIHPAWQVMS VSGDTKTSL- DLKACVNYTV QVRRSSRS--
Orizias_latipes_LEPR          YEVRYNTK-- -SDLAWQVVS VTGEPRLSL- DLQPEQEYTF QVRCSRLD--
Salmo_salar_LEPR              YEVRYNTSQS TSHLNWLHVE VSGCQWVSLT GLRPGLHYTV QIRSHHPA--
Oreochromis_mossambicus_LEPR  YEVRYSSG-- TTHPAWQVVS APGEPKVSL- DLKPELKYSV QVRCSGPE--
Tachysurus_fulvidraco_LEPR    YELRYSP-N- TSLTHWEVLN VQ-HPWVSLS ELTSGVRYTV QVRCKSLHHL
Epinephelus_coioides_LEPR     YEVRYSSN-- TTHPQWQVVS APEEPRLPL- ELKPRLNYTI QVRCSGLE--
Anguilla_anguilla_LEPRA       YDIRYSSS-- S-LHSWMRMS AEGAPGASLK GLSVGLNYTV QVRCKTPG--
Anguilla_anguilla_LEPRB       YDVRYSSN-- TSLNSWVHVN KVITQPVTLT GMNAGVTYTV QVRCKILG--
Lepisosteus_oculatus          YEVRYSAD-- TAEDSWLYTE GLMASHVLLN NLRPGVNYSA QVRCRNGN--
Homo_sapiens_GCSFR            ---------- ---------- ---------- ---------- ---------M
Danio_rerio_GCSFR             ---------- ---------- ---------- ---------- ---------M

                              201
Homo_sapiens_LEPR             -GPGIW-SDW STPRVFTT-- ----QDVIYF PPKILTSVGS NVSFHCIYKK
Macaca_mulatta_LEPR           -GPGIW-SDW STPHVFTT-- ----QDVIYF PPKILTSVGS NVSFHCIYKN
Bos_taurus_LEPR               -GLGIW-SDW SALLTFTT-- ----QDVIYF PPKILTAVGS NISFHCIYKN
Sus_scrofa_LEPR               -GPGIW-SDW STPFTFTT-- ----QDVIYF PPKILTSVGS NISFHCIYKN
Rattus_novegicus_LEPR         -GSGVW-SDW SLPQLFTT-- ----QDVMYF PPKILTSVGS NASFCCIYKN
Gallus_gallus_LEPR            -GPGFW-SEW STLYNLNV-- ---GAEVLYF PTKILTSVGS NVSFHCIYKN
Ornithorhynchus_anatinus_LEPR -GPGLW-SDW SPSHALNI-- ----QACFYF FPGITLLFQP SVNYHQRVKY
Xenopus_tropicalis_LEPR       -KSGLW-SDW SKTWVLNS-- ----QDVFYF PQKVLVSSGS STSVSCLFCD
Anolis_carolinensis_LEPR      -GSGLW-SDW SIPYSMNL-- ----QDVMYF PQKILASVGT NVSFYCISKT
Chrysemys_picta_bellii_LEPR   -GPGFW-SDW STPYNLNT-- ----EDVMYF PPKILTSVGS NVSFHCLYND
Latimeria_chalumnae_LEPR      -GLGFW-SNW STPYKLDA-- ----REVIYF PPRVLASAGS SVTISCLYNN
Danio_rerio_LEPR              -HLGYW-SEW SQPFYFKL-- -----DVSYI PAEVFTTQES EVTVYSVFHN
Carassius_carassius_LEPR      -HFSYW-SEW SQPFYFTL-- -----DVSYI PAEVFTAPGS EVTVSAVFHN
Takifugu_rubripes_LEPR        -DPPLW-SGW SESHHIFL-- ----DEVSYI PEKVVVKAGE NVTVYCVFND
Orizias_latipes_LEPR          -EPPLW-SEW SAPYKFYQ-- ----YIVTYI PEKMVARAGE SVTVYCLFNN
Salmo_salar_LEPR              -RPHLW-SDW SQQHRIRL-- ----ENVTYL PERVVASFGD SVTVYCVFND
Oreochromis_mossambicus_LEPR  -EPPLW-SEW SEPHHIRL-- ----DTVSYI PKIVVARPGE NVTVYCVFND
Tachysurus_fulvidraco_LEPR    HNWSEW-SNW SQPFFLTL-- -----DVSYI PAEVFTRPGA EVTVYGVVHN
Epinephelus_coioides_LEPR     -NPPVW-SDW SEPYHIYL-- ----DTVSYI PEKVVARPGD NVTVYCVFND
Anguilla_anguilla_LEPRA       -NQGVW-SDW SRPLYVYL-- ----NEVSYI PERLFTSTGS NVTIYCIFNN
Anguilla_anguilla_LEPRB       -KPGLW-SEW SQSLFIYL-- ----HEVTYL PKSVFTSEGA NVTVYCIFNN
Lepisosteus_oculatus          -KSGLW-SNW SIPLFIYL-- ----NEVTYL PAKILASTGS NVTIHCVFNN
Homo_sapiens_GCSFR            ARLGNCSLTW AALIILLLPG SLEECGHISV SAP-IVHLGD PITASCIIKQ
Danio_rerio_GCSFR             ASVSLEIKLW MYIYVVIKVT GASSCGNVYT PAP-VVLAGS PVSVSCSIEE
```

```
                                 251
Homo_sapiens_LEPR                ENKIVPSK-- EIVWWM-NLA EKIPQ-SQYD V-VSDHVSKV TFFNLNETKP
Macaca_mulatta_LEPR              ENKIVSSK-- KIVWWM-NLA EKIPQ-SQYD V-VSDHVSKV TFFNLNETKP
Bos_taurus_LEPR                  EKKIVSSK-- KIVWWL-NLA EKIPQ-SQYD V-VDDHISKV TFPNLNATKP
Sus_scrofa_LEPR                  ENKIVSSK-- KIVWWM-NLA EKIPQ-SQYD V-VGDHVSKV TFPNMNATKP
Rattus_novegicus_LEPR            ENQTISSK-- QIVWWM-NLA EKIPE-TQYN T-VSDHISKV TFSNLKATRP
Gallus_gallus_LEPR               KTQSVASK-- KIVWWL-NLA EEIPE-SQYT L-VNDRVSKV TLFNLKATKP
Ornithorhynchus_anatinus_LEPR    KNKYTPSQ-- KCELLPLFLR DVLP----LD S-IYCHCSCL SISRMNEFKM
Xenopus_tropicalis_LEPR          NGKKVPSS-- NITWWL-NFG EKIPK-HQYR T-TSDYFSKV FLTHLNTTKP
Anolis_carolinensis_LEPR         KDRIISSR-- KIIWWL-NLA KEIPR-SQYT I-VNDYISKV TLVNLSAMNP
Chrysemys_picta_bellii_LEPR      KNKMILSK-- KIVWWL-NLA EEIPE-RQYT L-VNDRVSRV TLFNLSATKP
Latimeria_chalumnae_LEPR         KSGT--VK-- DVDWWL-NLA VKIPE-NQYT I-INDHIASV TVTKLNATKN
Danio_rerio_LEPR                 RSW--SAS-- KAVWFL-NGK MKIPE-SQYR V-INEQVSTV TLKMDK----
Carassius_carassius_LEPR         RSW--SAS-- KAVWML-NGQ VKIPE-SHYR V-INEQVSAV TVTVDE----
Takifugu_rubripes_LEPR           HNF--NAS-- TALWTL-NFD QELDY-SLYH P-INQWVSQV TM---RPSET
Orizias_latipes_LEPR             RSM--NAS-- EAVWKL-NFH QLLHS---SQ S-VSGRVSKI TM---RASES
Salmo_salar_LEPR                 LRV--NAS-- TTVWIL-NSR DRLPK-SQYT A-VNDRVSKI TV---RPSEQ
Oreochromis_mossambicus_LEPR     HRM--NAS-- MAVWKL-NFK PPLQP-TLYH P-VNQWVSKI TV---RPSEN
Tachysurus_fulvidraco_LEPR       HSR--TAS-- KAVWML-NGL -VLPE-SQYQ T-INERVSAV TIRSKE----
Epinephelus_coioides_LEPR        HSI--NAS-- TAMWMR-NFQ QRLHS---SQ P-VNQWVSQI TV---RPSES
Anguilla_anguilla_LEPRA          RSH--SAK-- NAVWWL-SY- QKVPE-SQYT I-ISDHVSSV TLLNVKPLKQ
Anguilla_anguilla_LEPRB          QSL--SAR-- NVVWWL-NIQ EKVPE-SLYT I-VNDRVSRV TVPNVRPLKR
Lepisosteus_oculatus             RST--NAT-- NIVWWL-NTH EKVPV-SQYS T-INDHVSSV TLVNVKPHKR
Homo_sapiens_GCSFR               NCSHLDPE-P QILWRL-GA- ELQPGGRQQR LSDGTQESII TLPHLNHT--
Danio_rerio_GCSFR                DCPLTKGKVF YVQWRI-DG- QVVPRTYTYQ --ESNMTYSV LIPRLQDT--

                                 301
Homo_sapiens_LEPR                RGKFTYDAVY CCNE----HE CHH--RYAEL YVIDV-NINI SCET--DGYL
Macaca_mulatta_LEPR              RGKFTYDAVY CCNE----HE CHH--RYAEL YVIDV-NINI SCET--DGHL
Bos_taurus_LEPR                  RGKFTYDAVY CCNE----QE CHH--RYAEL YVIDV-NINI SCET--DGYL
Sus_scrofa_LEPR                  RGKFTYDAVY CCNE----HE CHH--RYAEL YVIDV-NINI SCET--DGYL
Rattus_novegicus_LEPR            RGKFTYDAVY CCNE----QA CHH--RYAEL YVIDV-NINI SCET--DGYL
Gallus_gallus_LEPR               RGSFFYNALY CCHQN---RE CHH--RYAEL YVVDV-NINI KCET--DGYL
Ornithorhynchus_anatinus_LEPR    NGSFILKATA LCFK----KE CPR--QECNL FQIDV-NINI SCET--DGYL
Xenopus_tropicalis_LEPR          KGKFRYDALH CCINH---NE CHH--RYAEI YVLDV-NISI SCET--DGNQ
Anolis_carolinensis_LEPR         GGKFLFNALY CCNEN---KE CNH--HYAEL YIIDV-NISI TCET--DGNL
Chrysemys_picta_bellii_LEPR      RGKFFYNALY CCNQN---RE CHH--RYAEL YVVDV-NINI TCET--DGYL
Latimeria_chalumnae_LEPR         RGKFHYDALY CCHRNGKNIT CNH--HYAEI HVIDV-KLNI SCET--DGNL
Danio_rerio_LEPR                 AG---FDTLM CCLTLGEKSM CSI--AYAKI YTEGRFNANI TCESE-YSYV
Carassius_carassius_LEPR         PG---FDTLM CCHQWGERFK CFI--AYAKI YTQGMFNADI TCQSK-NSEE
Takifugu_rubripes_LEPR           GM---YDLLQ CTKKR----- -MI--AYSQV YVEGA-SISI SCET--NGEI
Orizias_latipes_LEPR             RM---YDLLT CTQKA----- -AL--PYSQI SIEGA-SLDI RCET--NGNM
Salmo_salar_LEPR                 RL---SDTLH CCQPLGETYS CNY--RYSTI YIKDP-VIDI SCVT--NGDL
Oreochromis_mossambicus_LEPR     QM---YDLLS CTEGW----- -SI--PYSQI YVEGA-DIDI KCVT--NGDI
Tachysurus_fulvidraco_LEPR       PG---FDTLL CCYPFEQSYK CSI--AYTKV YVEGFFDANI TCTTEQHSSV
Epinephelus_coioides_LEPR        QM---YDLLQ CTQEW----- -TI--PYSQI YVQGA-SIDI TCET--NGDI
Anguilla_anguilla_LEPRA          RG---YDILH CCQREGEKSL CSF--PYAQI YVKDI-SVAI SCEV--DGDL
Anguilla_anguilla_LEPRB          QQ---YNVLQ CCQRSGETSL CSY--RYASL YTDGV-SVAI SCET--NGDL
Lepisosteus_oculatus             KGGIDYDVLQ CCQQNGENSV CNY--RYAAI YAKDI-NVTI SCET--NEHL
Homo_sapiens_GCSFR               -----QAFLS CCLNWGNSLQ ILDQVELRAG YPPAI-PHNL SCLMN-L-TT
Danio_rerio_GCSFR                -----SASIL CLVCVQEDCQ IVDGVEVKTG NPP-V-PQNL SCALT-LVKM

                                 351
Homo_sapiens_LEPR                TKMTCRWSTS TIQSLAESTL QLRYHRSSLY CSDIPSIHPI ---------S
Macaca_mulatta_LEPR              TKMTCRWSTN TIQSLAGSTL QLRYRRSSLY CFDIPSIHPI ---------S
Bos_taurus_LEPR                  TKMTCRWSPN AIQSLAGSNL QLRYHRSSLY CSDVPSIHPV ---------S
Sus_scrofa_LEPR                  TKMTCRWSTN AIQSLVGSTL QLRYHRSSLY CSDVPSVHPI ---------S
Rattus_novegicus_LEPR            TKMTCRWSPS TIQSLVGSTV QLRYHRRSLY CPDNPSIRPT ---------S
Gallus_gallus_LEPR               TKMTCRWSAN PNALLLGSSL QLKYHMSKIY CSNFPSTPPE ---------S
Ornithorhynchus_anatinus_LEPR    TKMTCRWPVD PKTLLTGSTL QLRYYRSSLY CADFPSAHHA ---------T
Xenopus_tropicalis_LEPR          KMMTCRWSSQ NMTLPEGSVL QFKYYRNKLY CLDKDLKGNV ---------P
Anolis_carolinensis_LEPR         QKMTCRWFTN GDPLLSECTL LLRYYRSDVY CSESPTISSY ---------S
Chrysemys_picta_bellii_LEPR      TKMTCRWSAN TNTLLVGSSL QLKYYRNSIY CSDFPSIPPK ---------S
Latimeria_chalumnae_LEPR         THMTCKWNPN TQNLPIGSSF KLKYYRNNIY CPSSQESQHN ---------S
Danio_rerio_LEPR                 DTMIYAYG-- ---------- ---------- -CRRQYRSKC T-EEAEEDTS
Carassius_carassius_LEPR         DTMRCEWNKS T---WA--QV RLLYSSRQHT MCETISEVEG S-EEAEESMS
Takifugu_rubripes_LEPR           DAMDCRWNST Q---WL--NP NFRTRWADLS CDVMEERERA GDNVG----H
Orizias_latipes_LEPR             DTMECSWNST Q---WL--SF NLQHKWTHMS CERMKEKEEA GDNVG----K
Salmo_salar_LEPR                 DSMTCRWNNL P---IG--GI NFMSRVADLS CDVMEEAERV ---GVPVGVV
Oreochromis_mossambicus_LEPR     DAMDCSWTHK Q---LT--KL RFRSKWADLS CDVMEESERA GENLG----E
Tachysurus_fulvidraco_LEPR       DTMTCKWNKS A---WA--VI RFLYRRYRRT CDEIQYEEGT LTQAQGDPMV
Epinephelus_coioides_LEPR        DAMTCSWKST Q---WT--RL KFRSRWADLQ CDVMEERERA GEKVG----E
Anguilla_anguilla_LEPRA          TAMTCTWNTS Q---WA--EV RCLYQRH-ML --PCEEEMEL ---------P
Anguilla_anguilla_LEPRB          SAMTCRWNIS L-------GV RFYYRTSDVP FDIAEEQMAV ---------S
Lepisosteus_oculatus             TNMTCKWNPN W---VLGSDV KFLYRKG-LS CGALEEDVRV ---------S
Homo_sapiens_GCSFR               SSLICQWEPG PET-HLPTSF TLKSFKSRGN CQTQG----- ---------D
Danio_rerio_GCSFR                PSLRCDWNPV QEIKNLPINY TLHVFRAKSQ KV-------- ----------
```

```
                                401
Homo_sapiens_LEPR               EPKDCYLQS- DGFYECIFQP IFLLSGYTMW IRINHSLGSL DSPPTCVLPD
Macaca_mulatta_LEPR             KPKDCYLQS- DGFYECVFQP IFLLSGYTMW IRINHPLGSL DSPPTCVLPD
Bos_taurus_LEPR                 EPKDCHLQR- DGFYECIFQP IFLLSGYTMW IRINHTLGSL DSPPACVIPD
Sus_scrofa_LEPR                 EPKDCQLQR- DGFYECIFQP IFLLSGYTMW IRINHPLGSL DSPPTCVIPD
Rattus_novegicus_LEPR           ELKNCVLQT- DGFYECVFQP IFLLSGYTMW IRINHSLGSL DSPPTCVLPD
Gallus_gallus_LEPR              EVKECHFQR- NHSYECTFQP VFLLSGYTMW IELKHSLGTL ESSPTCVVPA
Ornithorhynchus_anatinus_LEPR   DAKDCRFQD- EGFYECTFQP IYLLSGYTMW ITISHPLGTL ESPQKCVVPD
Xenopus_tropicalis_LEPR         ISKDCQLQM- DGFYECTFEP VHLVSGYIMW IEIQHHLGAL NSPPVCILPI
Anolis_carolinensis_LEPR        KIKECQPQR- NNSYECIFQP FYLLSGYTMW IEIKHSLGTV TSQPVCILPK
Chrysemys_picta_bellii_LEPR     EAKECHLQR- NHYYECTFQP IFLLSGYTMW IEIKHQLGKL ESPPTCVIPA
Latimeria_chalumnae_LEPR        DFKDCHLQR- NKLYECIFQP ISLFSGYIMW VEIYHYLGKL ESSPTCVIPM
Danio_rerio_LEPR                LVKECPSKA- GDHRQCTLSQ ISMIFCYKFW LEVEGGRG-- QSFPVYVTPI
Carassius_carassius_LEPR        LVKECPSGA- GDHRECTLRN LSLYSCYKFW LEVEGGHGKV RSFPVYVAPI
Takifugu_rubripes_LEPR          EGPSCLQVD- SRKRLCTIQP LRT-NCYKLW LEVSSHLGLI RSKPVYLTPN
Orizias_latipes_LEPR            IVDACYSI-- -KPRTCTFKP LRF-GCYKLW LELRTDSGSV RSKPIYLSSK
Salmo_salar_LEPR                RQAKCESSGY RGVKSCNLQP IRVTSCYKLW MEAKTD-NSM RSHPVYITPM
Oreochromis_mossambicus_LEPR    MGPACME--- GGQETCTIHP LRM-NCYKLW LELPSQLGPI RSKPVYLSPV
Tachysurus_fulvidraco_LEPR      DVEECTAGA- GDYYQCTLQD LSLISCYKLW LVVEDGYNKV RSLPVFVSPI
Epinephelus_coioides_LEPR       MGPSCLQVR- SKQKTCTIQP LRM-NCYKLW LEVPSRLGPI RSKPIYLSPI
Anguilla_anguilla_LEPRA         PAVECPVLG- --AKSCRLEP LFLTSCYIMW VEGREQEGTV KSHPIYILPM
Anguilla_anguilla_LEPRB         KEEECPSEG- RGLKSCTFQP FLPFSYYMMW LEFGTEEGTV KSQPVYALPM
Lepisosteus_oculatus            RVEECPVMG- WGSHKCTFQP LYLSFCYVMW LEIHHELGPV KSRPTYVTPM
Homo_sapiens_GCSFR              SILDCVPKDG QSHCCIPRKH LLLYQNMGIW VQAENALGTS MSPQLCLDPM
Danio_rerio_GCSFR               ----YAVPPG QHFYVVPRDA YGYFSELEIS VTAANVLGNT TSDPLKLTPL

                                451
Homo_sapiens_LEPR               SVVKPLPPSS VKAEI----- TINIGLLKIS WEKPVFP--- ENNLQFQIRY
Macaca_mulatta_LEPR             SVVKPLPPSS VKAEI----- IKNIGLLKIS WEKPVFP--- ENNLQFQIRY
Bos_taurus_LEPR                 SVVKPLPPSS VKAEI----- TVKIGLLKIS WEKPVFP--- ENNLQFQIRY
Sus_scrofa_LEPR                 SVVKPLPPSS VKAEI----- TAKIGLLKIS WEKPVFP--- ENNLQFQIRY
Rattus_novegicus_LEPR           SVVKPLPPSN VKAEI----- TINTGLLKVS WEKPVFP--- ENNLQFQIRY
Gallus_gallus_LEPR              DVVKPLPPSN IKAEI----- TRNDGLLNVS WTNPVFT--- NDDLKFQIRY
Ornithorhynchus_anatinus_LEPR   SLVKPLPPSS IKAEI----- TVYTGLLNVS WERPTFP--- ENHLQFQIRY
Xenopus_tropicalis_LEPR         NTVKPLAPSR VRAEM----- TKGSGHLYVS WKRPALP--- STDLQFQVRY
Anolis_carolinensis_LEPR        EVVKPFSPSN VKAEI----- TEEVGLLLVR WNNPEFP--- KYDLQFQIRY
Chrysemys_picta_bellii_LEPR     DVVKPFPPSN VKAEI----- TKNVGLLNVS WTNPAFP--- NSDLKFQIRY
Latimeria_chalumnae_LEPR        DVVKPLPPTS VEANI----- TKPDGQLNVT WEIPKLP--- EYDLQFQIRY
Danio_rerio_LEPR                DYVKPSPPFD LEAI------ TLPSKTLSVR WKRPSLP--- VYGMQYELQF
Carassius_carassius_LEPR        DYVKPDPPSD LEAT------ TLPNKTLSVS WRRPNLP--- VYDMQYELRF
Takifugu_rubripes_LEPR          DHVKPHTPTD VKAV------ SRSGGVLNVT WKRPYLP--- VE-VQCQFRY
Orizias_latipes_LEPR            GQVKPYTPTN VKAV------ TLRSGVLSVT WEPPSLP--- IDGLQYELQY
Salmo_salar_LEPR                DHVKPHPPSG LEAV------ SMPSGVLKLA WVPPELP--- IYDMQYQVRY
Oreochromis_mossambicus_LEPR    DHVKPHAPAN VKAV------ SHSSGVLEVT WQAPPLP--- ADGLQCQFQY
Tachysurus_fulvidraco_LEPR      DCVKPSPPSE LKAV------ TLPNKTLSAT WKRPYLP--- AYDLQYELRY
Epinephelus_coioides_LEPR       DHVKPHSPTN VKAV------ SRSSGVLLIS WEPPSLP--- VEGLQCQFRY
Anguilla_anguilla_LEPRA         DLVKPHPPFD LGAT------ FLPDAHLSIW WKRPELP--- VYELQFEVRY
Anguilla_anguilla_LEPRB         DLVKPYPPFD LEAV------ TVPEGYLRAT WKRPELP--- TYDLLFEVRY
Lepisosteus_oculatus            DVVKPHPPLN LEAEI----- TVPEGHLSIS WQRTELP--- VYDLQFEIRY
Homo_sapiens_GCSFR              DVVKLEPPML RTMDPSPEAA PPQAGCLQLC WEPWQPGLHI --NQKCELRH
Danio_rerio_GCSFR               NTVKFDPPSI TRIEA----- -HKYGCLKYS WSLSETQKWL QLTFIVQLRL

                                501
Homo_sapiens_LEPR               GLSG---KEV QWKM-YEVY- DAKSKSVSLP VPDLCAVYAV QVRCKRLDGL
Macaca_mulatta_LEPR             GLSG---KEI QWKM-YDVY- DAKSKSVSLP VPDFCAVYAV QVRCKRSDGL
Bos_taurus_LEPR                 GLSG---KQV QWKM-FEVY- DAKLKSASLP VPDLCAVYTV QVRCKSLDGL
Sus_scrofa_LEPR                 GLSG---KEV QWKI-YEVY- DTKLKSTSLP VPDLCAVYAV QVRCKRLDGL
Rattus_novegicus_LEPR           GLNG---KEI QWKT-HEVF- DAKSKSASLP VSDLCAVYVV QVRCRRLDGL
Gallus_gallus_LEPR              AVNR---EEL TWEL-YEVL- SVPTRSAVIE -VQLCVEYIV QIRCRALDGL
Ornithorhynchus_anatinus_LEPR   AVRG---KDG QWKT-YLHSP KAKSESASIE VLDLCMVYVV QVRCKRLDGL
Xenopus_tropicalis_LEPR         CLQG---QGI IWKV-LDIF- --EEEFVSIQ VPDVCASYTV QVRSRRTDGV
Anolis_carolinensis_LEPR        AANG---TKI NWEM-QEIS- TPSVSSAIVI VPDPCTVYIV QVRCSLTDGV
Chrysemys_picta_bellii_LEPR     SVNR---EEI LWEI-FEVS- NAPTRSVMIK VLDLCVVYIV QVRCSGLDGL
Latimeria_chalumnae_LEPR        SKNG---KEK NWKT-QDVV- --MTSSGIVE VSNPCTIYIV QVRCTRYEGP
Danio_rerio_LEPR                KAL-AGMANT QWKV-IGPL- --LEPQAEIQ LEESCVQFKV EVRCKDVNDT
Carassius_carassius_LEPR        VAL-RDMPNT QWKV-IGPL- --LEPQAEVQ LAESCVQFNV EVRCRRLNGS
Takifugu_rubripes_LEPR          HSPSADHPKP DWKV-QAIV- --REPWAEVN VSDVCRVFVV QVRCMHISGA
Orizias_latipes_LEPR            HPLSTV--KE EWKV-QRSK- --QPPPMTVQ VPEMCRSYVV QVRCMHIAGK
Salmo_salar_LEPR                ALS-TGRAHP FWQV-LALQ- --TESWAEVL EPDVCGVYNV QVRCRHINGS
Oreochromis_mossambicus_LEPR    HSPSTVSPRP KWKL-QDPV- --RVPWAEVA VPDMCRVYVV QVRCKHTNGT
Tachysurus_fulvidraco_LEPR      VSM-HGMVDL KWKV-FGSL- --LESRATFT VLDPCIQYQV QVRCRRLNGP
Epinephelus_coioides_LEPR       HSPSAVRAQP EWKI-QSPV- --RVPWAEVL VPDMCRVYVV QVRCMHTSGT
Anguilla_anguilla_LEPRA         SVD-K--DDT LQKV-IRSV- --SNQSAVVP IVDPCVVYTI QVRCKRLAGP
Anguilla_anguilla_LEPRB         AVD-G--PDP LWRV-YKSE- --VNLTVVFP VSDPCAVYTI MVRCKRLHGS
Lepisosteus_oculatus            AVD-G--PNK QWKL-LRTV- --YNETVVTQ VADPCVVYTV QIRCKRFNGP
Homo_sapiens_GCSFR              KPQR---GEA SWAL-VGPL- PLEALQYELC GLLPATAYTL QIRCIRWPLP
Danio_rerio_GCSFR               KTVS---NQP NKDLVYTSR- QLQLNPIEVC SLLHWTDYRS TVRV-KYYAT
```

```
                               551
Homo_sapiens_LEPR              GYWSNWSNPA YTVVMDIKVP MRGPEFWRII NGDTMKKE-- KNVTLLW-KP
Macaca_mulatta_LEPR            GLWSNWSNPA YTVVMDIKVP MRGPEFWRII NGDTMKKE-- KNVTLLW-KP
Bos_taurus_LEPR                GYWSNWSTPA HTVVMDVKVP IRGPEFWRLI SEDTTKKE-- RNVTLLW-KP
Sus_scrofa_LEPR                GYWSNWSTPA YTVVTDVKVP IRGPEFWRII NEDATKKE-- RNITLLW-KP
Rattus_novegicus_LEPR          GYWSNWSSPA YTLVMDVKVP MRGPEFWRIM DGDITKKE-- RNVTLLW-KP
Gallus_gallus_LEPR             GYWSNWSRSA YAAVKDIQAP LHGPEFWRTV TEDPATGQ-- KNVTLLW-KP
Ornithorhynchus_anatinus_LEPR  GYWSEWSNPA STLIRDVKAP VRGPEFWRVI KEDSGKKA-- RNITLFW-KP
Xenopus_tropicalis_LEPR        GYWSDWSQPV HTVVRDIRVP LQGPTFWRTT HNNPMQKG-- DNISIIW-QP
Anolis_carolinensis_LEPR       GYWSDWSRPA YTVIKDIKAP LRGPEFWRIV DEDPVTNQ-- NNVTLFW-KP
Chrysemys_picta_bellii_LEPR    GYWSDWSKPA YTIVQDIQAP LRGPEFWRVI NEDPIRKQ-- KNVTLVW-KP
Latimeria_chalumnae_LEPR       GYWSAWSGPA YTTVYDLKTP EKGPDFWRVI NEDPLTKV-- TNVTLLW-QP
Danio_rerio_LEPR               GYWSDWSNSH ISTVFNLKAP EMGPDFWRIL QEDPTRNVT- -NVTLIFKQP
Carassius_carassius_LEPR       GYWSDWSMSY TSVVYNRKAP EMGPDFWRII QEDPLRNVTN TNVTLIIKQP
Takifugu_rubripes_LEPR         GYWSEWSPSV YSSPQNSRAP ERGPNFWRFL QDDPHRKQ-- TNVTLLFKDL
Orizias_latipes_LEPR           GYWSEWSDLI YSTPNNSKAP ERGPDFWRIR QDNQHINK-- SNITLLFEHF
Salmo_salar_LEPR               GTWSDWSHLL YTTTHNSRAP ERGPDFWRVF QEDPASTQ-- TNVTLLF-EH
Oreochromis_mossambicus_LEPR   GYWSDWSESV YSTPQNSRAP ERGPDFWRIR QDDPHGNQ-- SNITLLFENF
Tachysurus_fulvidraco_LEPR     GYWSDWSYTH ASSVYNVKAP EMGPDFWRII QETPEPY--- TNVTLLF-KP
Epinephelus_coioides_LEPR      GHWSEWSDSV YSTPQNSRAP ERGPDFWRVL QDDPYRNQ-- TNVTLLFEQH
Anguilla_anguilla_LEPRA        GFWSDWSSPY YTTINNIKAP EQGPDFWRVL QEYPKLNQ-- THVTLLF-TL
Anguilla_anguilla_LEPRB        GFWSEWSDPH YSAVQISRAP ERGPDFWRVL KDDRERNQ-- SNVTLLF-AP
Lepisosteus_oculatus           GHWSTWSSPV YTVVHDIQAP EQGPDFWRMI REDPAMKQ-- TNVTLLI-KP
Homo_sapiens_GCSFR             GHWSDWSPSL ELRTTERAPT VRLDTWWRQR QLDPRTVQ-- ----LFW-KP
Danio_rerio_GCSFR              SEWSEWSDPK TATTLNKAPA GRLDTWLKV- --NNQTAQ-- ----LYW-KP

                               601
Homo_sapiens_LEPR              -LMKNDSLCS VQRYVINHHT SCNGTWSEDV G--NHTKFTF LWTEQAHTVT
Macaca_mulatta_LEPR            -LMKNESLCS VQRYVINHHT SCNGTWSEDV G--NHTKFTF LWTEQAHTVT
Bos_taurus_LEPR                -LMKNDSLCS VRRYVVKHHT SHNGTWLEDV G--NHTKLTF LWTEQAHSVM
Sus_scrofa_LEPR                -LMKNDSLCS VRSYVVKHHT SRHGTWSEDV G--NHTKLTF LWTEQAHSVT
Rattus_novegicus_LEPR          -LMKNDSLCS VRRYVVKHRT AHNGTWSQDV G--NQTNLTF LWAESAHTVT
Gallus_gallus_LEPR             -LMKNHSLCS VSRYVIKHQT SENTSWSEYV D--NGTTCSF PWTESTHTIT
Ornithorhynchus_anatinus_LEPR  -LTRNQSLCS VRSYWVEHHT SNQVTWTHDA G--NSTESTF PWTGQDHTVS
Xenopus_tropicalis_LEPR        -LPSEHSLCS IQGYEVIHLN SKNVTWSKYV G--NTTKHTF TLSDNAVTVT
Anolis_carolinensis_LEPR       -LMKNLSLCS VLGYMVEHTT SDNVTWSDYV E--NDTTYTF SWAEDVHTIK
Chrysemys_picta_bellii_LEPR    -LMKNYSLCS VCGYIVKHHT SENITWTEYV T--NGTTYTY PWMEDADTIT
Latimeria_chalumnae_LEPR       -V---EVLCT VKGFRIQYQT SKNVTWTEHS R--NETSYTF TWMSSIHTVS
Danio_rerio_LEPR               -ILAGDPNSC VEGLVIKHQA SGGVMWSNET T--LARFHSF QWRKEAHTVT
Carassius_carassius_LEPR       -ILAGDPYSC VEGLVFEHQA SGRAVWSNET T--LVQFHSF QWRKEAHTVT
Takifugu_rubripes_LEPR         --QTSGQPYC VEGFLVKRLG STGPV-QEPI L--MQSSYSF EWNQMPQTVT
Orizias_latipes_LEPR           --PGTWNSYC VDGFIVQHEA SNRSVVRKQI N--LGSSYSF EWNQEPQTVT
Salmo_salar_LEPR               -SPIVEPTYC VEELVVQHQD SGGTVTEERI G--LVSSYSF EWRKEVHSVT
Oreochromis_mossambicus_LEPR   --PPSGNSYC VDGFVVQRRS SSGSVLRETI E--LMSSYSF EWNQELQTVT
Tachysurus_fulvidraco_LEPR     -LPEVEAAIC VQGLVVVHQT SGGNVWSDDI I-APSSFYTF QWREEVHSIT
Epinephelus_coioides_LEPR      HLQLSARSYC IDGFIVQYQA LSGSVMREQI E--LASSFSF EWNQVPQTVT
Anguilla_anguilla_LEPRA        -SQSEGPFCC VEGLTVKHQT SRGSVWSENL G--RVSTYSF SWTEDVHTVT
Anguilla_anguilla_LEPRB        -LTGEGTLCC VTGIVVQHQT TGGAVWIEQL G--LVSTYTF PWREEVHTVT
Lepisosteus_oculatus           -LRKEDLFCS VDGLIVQHQT SSDVLRYEYL N--ITTTYSF PWIEDVHSVS
Homo_sapiens_GCSFR             -VPLEEDSGR IQGYVVSWRP SGQAGAILPL CNTTELSCTF HLPSEAQEVA
Danio_rerio_GCSFR              -SQQFRANGQ NLSYSVDSK- ----DTKKRL CVTTETYCFF SLTKWDKKIF

                               651
Homo_sapiens_LEPR              VLAINSIGAS VANFNLTFSW PMSKVNIVQS LSAYPLNSSC VIVSWILSPS
Macaca_mulatta_LEPR            VLAINSIGAS VANFNLTFSW PMSKVNIVQS LSAYPLNSSC VILSWILSPS
Bos_taurus_LEPR                VLAINSIGAS SANFNLTFSR AISKVNIVQS LSAYPLNSSC VILSWMLSPS
Sus_scrofa_LEPR                VLAVNSIGAS SANFNLTFSW PMSKVNIVQS LSAYPLNSSC VGLSWLLSPS
Rattus_novegicus_LEPR          VLAINSIGAS LVNFNLTFSW PMSKVNAVQS LSAYPLSSSC VILSWTLSPN
Gallus_gallus_LEPR             ILAVNSIGAS SVNFNLTLSQ QMSTVNAVQS LIAYPVNSTC VILTWTLSPQ
Ornithorhynchus_anatinus_LEPR  VVATNSVGSS SANFNLTFSR PISEVDIVQS LGAYPLNSSC VILSWVLSSH
Xenopus_tropicalis_LEPR        LLAVNSLGYS LTNSKLTFSC EMSTVTSVES FRVYHMNNTC AVAVWTMLPK
Anolis_carolinensis_LEPR       VIAINSIGAS SVNFILTLSK QMSTVNIVES LRIYPVNSSC VIVTWTLSPV
Chrysemys_picta_bellii_LEPR    ILAVNSVGAS SMNFNLTLSQ QMSTVNIVQS LSAYPVNSSC VILTWTLAPQ
Latimeria_chalumnae_LEPR       ILVFNSIGSS TVNYNLTLSK QTSTVQVVQS LHAYLSNSSC VILSWNLLPG
Danio_rerio_LEPR               VMSRNALGIS TWNRNITLLR QAK-RRCVRS FSA-VANVSC VHLSWSLLSD
Carassius_carassius_LEPR       VMSRNALGIS TRNRNITLFH QPK-RRVVRS FSV-VANASC VHLSWSLLHD
Takifugu_rubripes_LEPR         VEAFNSLGSS SDNINMTLEK SPK-RRCVHH FSVTVINSTC VSLSWTLIDK
Orizias_latipes_LEPR           VEAYNSLGNS TNNKNMTLGK TSR-RKAVHS VHALVLNSTH VSLSWSLLND
Salmo_salar_LEPR               VKAQNSQGSS TRNTHMTLDR HPK-RQCVRL FSASRVNSSC VVLLWSLQPN
Oreochromis_mossambicus_LEPR   VEAYNSLGNS RDNINMTLER QPK-GHCVRS FHVLLINGTC VSLSWSLLEN
Tachysurus_fulvidraco_LEPR     VMSRNSLGSS AENSNMTLVR QPK-RQCVRW FHV-TANASC VFLSWSLLSE
Epinephelus_coioides_LEPR      VEAYNNLGSS ANNFNMTLER QPK-RRSVRS FSVLVINSTC VSLSWTLLDN
Anguilla_anguilla_LEPRA        VLAHNALGSS TKNSNMTLTR HTK-SQSVHS FSSMMVNSSC VALSWTLFPN
Anguilla_anguilla_LEPRB        VMAINSLGPS TRNTHMTLMR KASKPRSVSS FSSVMINDSC VALVWSLFPN
Lepisosteus_oculatus           VLAFNSVGSS VMNYNLTFTR HTRKVQTVQS FSCAMVNSSC VALSWNLLPN
Homo_sapiens_GCSFR             LVAYNSAGTS RPTPVVLSES RGPALTRLHA M---ARDPHS LWVGWE-PPN
Danio_rerio_GCSFR              LRARNEVGFS DHNEVPVAVH RNKGLEPVSN FSVHPQSNTS LHVIWK-SPA
```

```
                               701
Homo_sapiens_LEPR              DYKLMYFIIE WKNLNED--- -----GEIKW LRISSSV-KK YYIHD-----
Macaca_mulatta_LEPR            DYKLMYFIIE WKNLNED--- -----GEIKW LRISSSV-KK YYIHD-----
Bos_taurus_LEPR                DYNLMYFILE WKILNED--- -----SEIKW LRIPSSV-KK YYVHD-----
Sus_scrofa_LEPR                DYNLMYFILE WKILNED--- -----HEIKW LRIPSSV-KK YYIHD-----
Rattus_novegicus_LEPR          DYSLLYLVIE WKNLNDD--- -----DGMKW LRIPSNV-NK YYIHD-----
Gallus_gallus_LEPR             IYVITSFIIE WRNLNKE--- -----EEMKW VQVPPNI-SK HYIYD-----
Ornithorhynchus_anatinus_LEPR  GYNLKSLVIE WKNLNED--- -----NEMKW LRVSTNV-NK YYIHD-----
Xenopus_tropicalis_LEPR        SDMPLEFIVE WKNLGNE--- -----EKVQW MNIPRNM-SR CYIED-----
Anolis_carolinensis_LEPR       SYIITSFVIE WINLNGE--- -----EQIKW IMVPSDI-RR HHIFD-----
Chrysemys_picta_bellii_LEPR    MYVITSFVIE WKNLNEE--- -----EQMKW IRVAPNI-SK YYIYD-----
Latimeria_chalumnae_LEPR       DYLLSSFIIE WKILNEK--- -----ENIKW IRVPSDF-NK FPIKD-----
Danio_rerio_LEPR               QPVPQSFVIE WLDLNKDPEK DVSLTERIQW VRVESRS-RD LSLCPRAKHP
Carassius_carassius_LEPR       HPVPQSFVIE WLDLNKDPEQ DMSLIERLQW VRVQSTA-RD LSLCR-----
Takifugu_rubripes_LEPR         SSPPIFMVVQ WSLLWKQDSG RPRGQSTDTW VRLPYTD-GP TYLGG-----
Orizias_latipes_LEPR           GIVPLFMVVQ WSES------ --SGLSGLKW ARLPYSN-HV VYIKG-----
Salmo_salar_LEPR               SSVPWSLVVE WSGQNHQDRP DQTSESRERW TRFPPTD-KL LYLYG-----
Oreochromis_mossambicus_LEPR   SSVPLFMVVE WLPHKQQDS- ---GPRAETW TRLRYTD-HP VYLRG-----
Tachysurus_fulvidraco_LEPR     QPSLLSFVLE WQEQSGVSSQ GWASDGRVEW LRVASTA-RD LQLCR-----
Epinephelus_coioides_LEPR      NSVPLFMVVQ WSPHKQQDSD HHKGRIGETW ARLPYTD-HP IYLKG-----
Anguilla_anguilla_LEPRA        SSAPSSFVIQ WSGQSRSRQ- QDKQGGRVKW VRVPPNN-RA FHLHE-----
Anguilla_anguilla_LEPRB        SSAPASFVVE WSSRSRGRG- RGDAWLRVKW VRVSAPS-RS LYLHD-----
Lepisosteus_oculatus           RSLPESFIIE WKLQNREKQ- LQNTDEVVKW VRAPPKI-HT FYLYD-----
Homo_sapiens_GCSFR             -PWPQGYVIE WGLGPPSASN S-----NKTW RMEQNGRATG FLLKE-----
Danio_rerio_GCSFR              FSNVTSYVLE WRSLCGTTAA P-----L-SF TLIHKN--KS NTTLT-----

                               751
Homo_sapiens_LEPR              ---------- --------HF IPIEKYQFSL YPIFMEGVGK PKIINSFTQD
Macaca_mulatta_LEPR            ---------- --------HF IPIEKYQFSL YPIFMEGVGK PKIINSFAQD
Bos_taurus_LEPR                ---------- --------YF IPIEKYQFSL YPIFTEGVGK PKIINSFAQD
Sus_scrofa_LEPR                ---------- --------HF IPIEKYQFSL YPIFMEGVGK PKIINSFTQD
Rattus_novegicus_LEPR          ---------- --------NF IPIEKYQFSL YPVFMEGVGK PKIINGFTKD
Gallus_gallus_LEPR             ---------- --------HF ILIEKYRFSL YPVFAAGVGK SRATDQFSKD
Ornithorhynchus_anatinus_LEPR  ---------- --------HF IFIEKYQFTL YPVFLEGVGK PKMTNQFIRD
Xenopus_tropicalis_LEPR        ---------- --------NF FAIEKYVFSL YPVFPEGVGR SKVVNGFSTV
Anolis_carolinensis_LEPR       ---------- --------NF ILIDKYRFSL YPITCEGVMK PYVTDGFSKG
Chrysemys_picta_bellii_LEPR    ---------- --------HF ILIEKYQFSL YPISPEGVGN PKTTDEFIKD
Latimeria_chalumnae_LEPR       ---------- --------HF PVFEKYQFTL YPILSDGVAQ PLMIDEFFKG
Danio_rerio_LEPR               ERKESPLVFD NIICFYAGRF YGSEE--FTL YPVFADGEGE PARYTAT---
Carassius_carassius_LEPR       ---------- --------RF YGSEE--FTL YPVFVDGEGE PVRYTAT---
Takifugu_rubripes_LEPR         ---------- --------HF FGSEDYGFYL YPVFAHGEGE PAFATAT---
Orizias_latipes_LEPR           ---------- --------SF SRSEDYSFHL YPVFADMEGE PMYIIAA---
Salmo_salar_LEPR               ---------- --------HF YDTDEYEFIL YPVFADGEGE PVYTKVF---
Oreochromis_mossambicus_LEPR   ---------- --------DF FASEEYGFFL YPVFAEGEGE PIYTLAT---
Tachysurus_fulvidraco_LEPR     ---------- --------PF YGTEE--FKL YPVFVDGEGE AVRCTAV---
Epinephelus_coioides_LEPR      ---------- --------DF FGSEECGFYL YPVFADGEGE PVYAIAS---
Anguilla_anguilla_LEPRA        ---------- --------TF FASEEYQFIL YPIFENTEGE PIYAK-D---
Anguilla_anguilla_LEPRB        ---------- --------RF YVSEEYQFAL HPIFANGEGE PFYNKED---
Lepisosteus_oculatus           ---------- --------TF FLSEEYLFIL YPIFLEGEGE PIYK--D---
Homo_sapiens_GCSFR             ---------- --------NI RPFQLYEIIV TPLYQDTMGP SQHVYAYSQE
Danio_rerio_GCSFR              ---------- --------GL EPSKPYEISI YPRYVKGIGR PVTVLAYSSE

                               801
Homo_sapiens_LEPR              DIEKH----- ---------- ---------- ---------- ----------
Macaca_mulatta_LEPR            NTEKH----- ---------- ---------- ---------- ----------
Bos_taurus_LEPR                D-EKH----- ---------- ---------- ---------- ----------
Sus_scrofa_LEPR                G-EKH----- ---------- ---------- ---------- ----------
Rattus_novegicus_LEPR          DIAKQ----- ---------- ---------- ---------- ----------
Gallus_gallus_LEPR             GYA-S----- ---------- ---------- ---------- ----------
Ornithorhynchus_anatinus_LEPR  ENEKR----- ---------- ---------- ---------- ----------
Xenopus_tropicalis_LEPR        ELTEA----- ---------- ---------- ---------- ----------
Anolis_carolinensis_LEPR       EME-N----- ---------- ---------- ---------- ----------
Chrysemys_picta_bellii_LEPR    RSE-K----- ---------- ---------- ---------- ----------
Latimeria_chalumnae_LEPR       EREK------ ---------- ---------- ---------- ----------
Danio_rerio_LEPR               -R-------- ---------- ---------- ---------- ----------
Carassius_carassius_LEPR       -R-------- ---------- ---------- ---------- ----------
Takifugu_rubripes_LEPR         -R-------- ---------- ---------- ---------- ----------
Orizias_latipes_LEPR           -K-------- ---------- ---------- ---------- ----------
Salmo_salar_LEPR               -RGGD----- ---------- ---------- ---------- ----------
Oreochromis_mossambicus_LEPR   -R-------- ---------- ---------- ---------- ----------
Tachysurus_fulvidraco_LEPR     -R-------- ---------- ---------- ---------- ----------
Epinephelus_coioides_LEPR      -R-------- ---------- ---------- ---------- ----------
Anguilla_anguilla_LEPRA        -RGRP----- ---------- ---------- ---------- ----------
Anguilla_anguilla_LEPRB        -RGRP----- ---------- ---------- ---------- ----------
Lepisosteus_oculatus           -KRKP----- ---------- ---------- ---------- ----------
Homo_sapiens_GCSFR             MAPSHAPELH LKHIGKTWAQ LEWVPEPPEL GKSPLTHYTI FWTNAQNQSF
Danio_rerio_GCSFR              TAPSDAPELN YEEISRSHLK FHWGQIPLEK RNGIIQGYRF YFWHNKNEIK
```

```
                                851
Homo_sapiens_LEPR               ---------- -----QSDAG LYVIVPVII- ---------- ----------
Macaca_mulatta_LEPR             ---------- -----QNDAG LYVIVPVII- ---------- ----------
Bos_taurus_LEPR                 ---------- -----QHDAD LYVIVPIII- ---------- ----------
Sus_scrofa_LEPR                 ---------- -----RNDAG LYVIVPIII- ---------- ----------
Rattus_novegicus_LEPR           ---------- -----QNDAG LYVIVPIII- ---------- ----------
Gallus_gallus_LEPR              ---------- -----QTSSN LYMVLPIVI- ---------- ----------
Ornithorhynchus_anatinus_LEPR   ---------- -----QSDAG LYVIVPIII- ---------- ----------
Xenopus_tropicalis_LEPR         ---------- -----PKDAG LYVILPVIS- ---------- ----------
Anolis_carolinensis_LEPR        ---------- -----HNDVN VYVILPLVI- ---------- ----------
Chrysemys_picta_bellii_LEPR     ---------- -----RNDAG LYVILPIVI- ---------- ----------
Latimeria_chalumnae_LEPR        ---------- -----IKSDG SYIVLPIII- ---------- ----------
Danio_rerio_LEPR                ---------- ------GDPA AYILLLIIA- ---------- ----------
Carassius_carassius_LEPR        ---------- ------GGPA AYILLLIIA- ---------- ----------
Takifugu_rubripes_LEPR          ---------- ------RDPA IYMMLMMIS- ---------- ----------
Orizias_latipes_LEPR            ---------- ------RNPA AYMIIMSIS- ---------- ----------
Salmo_salar_LEPR                ---------- -----A-GPA AYMLLMIIA- ---------- ----------
Oreochromis_mossambicus_LEPR    ---------- ------GDPK AYMMLMIIS- ---------- ----------
Tachysurus_fulvidraco_LEPR      ---------- ------SDPA AYMLLMIIA- ---------- ----------
Epinephelus_coioides_LEPR       ---------- ------GDPA AYMMLMIIS- ---------- ----------
Anguilla_anguilla_LEPRA         ---------- -----RGDHA AYMLLLIIT- ---------- ----------
Anguilla_anguilla_LEPRB         ---------- -----SAQHA AYALLLIIA- ---------- ----------
Lepisosteus_oculatus            ---------- -----KGEQA AYILLLLIA- ---------- ----------
Homo_sapiens_GCSFR              SAILNASSRG FVLHGLEPAS LYHIHLMAAS QAGATNSTVL TLMTLTPEGS
Danio_rerio_GCSFR               E-I-MTTETS VEVKDLQPHT KYHALLSICT KGGCVNGSFS TLTTERLDGI

                                901
Homo_sapiens_LEPR               ---------S SSILLLGTL- ---------- LISHQRMKKL FWEDVPNPKN
Macaca_mulatta_LEPR             ---------S SSILLLGTL- ---------- LILHQRMKKL FWEDVPNPKN
Bos_taurus_LEPR                 ---------S SSILLLGIL- ---------- SVSHQRMKKL FWEDVPNPKN
Sus_scrofa_LEPR                 ---------S SSILLLGTL- ---------- LMSHQRMKKL FWEDVPNPKN
Rattus_novegicus_LEPR           ---------S SCVLLLGTL- ---------- LISHQRMKKL FWDDVPNPKN
Gallus_gallus_LEPR              ---------S TSVLLLGAL- ---------- LVSHRRMKKL LWEDVPNPKN
Ornithorhynchus_anatinus_LEPR   ---------S SSVLLFGTL- ---------- LISHQRMKKV FWEDVPNPKN
Xenopus_tropicalis_LEPR         ---------F SVFLLMGTI- ---------- LISHQRMKKL FWKDVPNPKH
Anolis_carolinensis_LEPR        ---------S CSVLLFGGF- ---------- LILHQRMKTL FWDDVPNPKN
Chrysemys_picta_bellii_LEPR     ---------S SFVLLLGTL- ---------- LISQQRMKKL FWEDVPNPKN
Latimeria_chalumnae_LEPR        ---------S SLILLFGTL- ---------- LVSQQRMRKL LWEDVPNPQN
Danio_rerio_LEPR                ---------F LSVVLFVTL- ---------- MMSQNQMKKL MWKDVPNPNK
Carassius_carassius_LEPR        ---------F LSVVLFVTL- ---------- LMSQNQMRKL TWKDVPNPNN
Takifugu_rubripes_LEPR          ---------F LSIVLLISL- ---------- ILSQNQMKKL MWKDVPNPNQ
Orizias_latipes_LEPR            ---------F LCIL-LLTL- ---------- VLTQNQIKRN ---LVPNPKK
Salmo_salar_LEPR                ---------F LSIVLFVTL- ---------- VISQNHMKKF MWKDVPNPNN
Oreochromis_mossambicus_LEPR    ---------F LFIILFVTL- ---------- VLSQNQMKKF VSKDVPNPRK
Tachysurus_fulvidraco_LEPR      ---------F LFVVLFVTL- ---------- IISQNQLKKL MWRDVPNPNN
Epinephelus_coioides_LEPR       ---------F LSIVLFVTL- ---------- ILTQNQMKRF VWKDVPNPNK
Anguilla_anguilla_LEPRA         ---------F LSVVLFVTL- ---------- AVSQNQMRKL VWKDVPNPNN
Anguilla_anguilla_LEPRB         ---------F MSVVLFLTL- ---------- AASQRQMMKL VWKDVPNPNN
Lepisosteus_oculatus            ---------F LSVVLFVTL- ---------- AVSQHQMKKL VWKDVPNPNN
Homo_sapiens_GCSFR              ELHIIL--G- ----LFGLLL LLTCLCGTAW LCCSPNRKNP LWPSVPDPAH
Danio_rerio_GCSFR               EMVIFVIPAC IGASLLVIII VFTCFGK--- ---QERVKMC LWPIIPDPAN

                                951
Homo_sapiens_LEPR               CSWAQGLNFQ K--------- ---------- ---------- --PETFEHLF
Macaca_mulatta_LEPR             CSWAQGLNFQ KIRGFVMLPR LVLNSQAQVI HPPRPPKVLE LQPETFEHLF
Bos_taurus_LEPR                 CSWAQGLNFQ K--------- ---------- ---------- --PETFEHLF
Sus_scrofa_LEPR                 CSWAQGLNFQ K--------- ---------- ---------- --PETFEHLF
Rattus_novegicus_LEPR           CSWAQGLNFQ K--------- ---------- ---------- --PETFEHLF
Gallus_gallus_LEPR              CSWAQGVDFQ Q--------- ---------- ---------- --PETFEHLF
Ornithorhynchus_anatinus_LEPR   CSWAQGVNFQ K--------- ---------- ---------- --PESFEQLF
Xenopus_tropicalis_LEPR         CSWAQGVNFE K--------- ---------- ---------- --PDTLENLF
Anolis_carolinensis_LEPR        CSWAQGVNFQ K--------- ---------- ---------- --PETFEHLF
Chrysemys_picta_bellii_LEPR     CSWAQGVNFQ K--------- ---------- ---------- --PETFEHLF
Latimeria_chalumnae_LEPR        CSWAQGVNFQ K--------- ---------- ---------- --PETLDDLF
Danio_rerio_LEPR                CSWAKGMDFR Q--------- ---------- ---------- --IDTMESLF
Carassius_carassius_LEPR        CSWAKGMDFR Q--------- ---------- ---------- --IDTMENLF
Takifugu_rubripes_LEPR          CSWARGIDLN ---------- ---------- ---------- ----AFDHMF
Orizias_latipes_LEPR            CSWAKGIDFQ K--------- ---------- ---------- --VDTFD-LF
Salmo_salar_LEPR                CSWAQGIDFG K--------- ---------- ---------- --ADTMEQLF
Oreochromis_mossambicus_LEPR    CSWAKGIDFK K--------- ---------- ---------- --VDTFDYLF
Tachysurus_fulvidraco_LEPR      CSWAKGIDFK K--------- ---------- ---------- --LD--GNLF
Epinephelus_coioides_LEPR       CSWAKGLDLK K--------- ---------- ---------- --ADNFDHLF
Anguilla_anguilla_LEPRA         CSWAQGVDFR K--------- ---------- ---------- --AETIESLF
Anguilla_anguilla_LEPRB         CSWAQGVDFR K--------- ---------- ---------- --AEAVENLF
Lepisosteus_oculatus            CSWAQGVDFK R--------- ---------- ---------- --AETIENLF
Homo_sapiens_GCSFR              SSLGSWVPTI ---------- ---------- ---------- ----MEEDAF
Danio_rerio_GCSFR               SSIKRWTTTD ---------- ---------- ---------- ----SLQGLP
```

```
                                 1001
Homo_sapiens_LEPR                IKHTASVT-C GPLLL----- -----EPETI SEDISVDTSW KNKDEMMPTT
Macaca_mulatta_LEPR              IKHTASVT-C GPLLL----- -----EPETI SEDISVDTSW KNKDEMVPTT
Bos_taurus_LEPR                  IKHTESTT-F GPLLL----- -----EPETI SEDISVDTSW KNKDEMVPAT
Sus_scrofa_LEPR                  IKHTESVT-F GPLLL----- -----EPETI SEDISVDTSW KNKDEMVPPT
Rattus_novegicus_LEPR            TKHAESVI-F GPLLL----- -----EPEPV SEEISVDTAW KNKDEMVPAA
Gallus_gallus_LEPR               VKHPEAMS-F EPLLL----- -----EPEIV LEDISVTKAL EQEDTQDFLV
Ornithorhynchus_anatinus_LEPR    TKPPEAVA-F GPLLL----- -----EPEAV SEDVTVDSPW SGEGEQYSVA
Xenopus_tropicalis_LEPR          MKHHKHPANG SPFLF----- -----EPEAV FEDLSIDKQV PHEIIDNIPA
Anolis_carolinensis_LEPR         LKHPEALS-F GPLLL----- -----EPEIV LEDVTIDKAR NNEEKQDLRA
Chrysemys_picta_bellii_LEPR      IKHPEAIS-F GPLLL----- -----EPEIV LEDINVAKAL KSEDKQDLLA
Latimeria_chalumnae_LEPR         VRPHGKFA-I SPFLP----- -----ATETV SEAFSIEKII HIEQRKGATA
Danio_rerio_LEPR                 P-HSEGLT-A CPLLL----- -----VSESI CEVEIIEKPH PLTIENV---
Carassius_carassius_LEPR         P-HSEGLT-A CPLLL----- -----VSESI CEVEIIEKPH PVTLEHE---
Takifugu_rubripes_LEPR           H-PPEGFP-A WPLLL----- -----PPEKI SNLVIVDKAD LSALSTP---
Orizias_latipes_LEPR             Q-PAEGLQ-I CPLLP----- -----SDNII SKVIIMEKVE KRAF----ME
Salmo_salar_LEPR                 L-HPEGLP-A WPLLL----- -----VSETI SQATIMEKTG PPTSVP----
Oreochromis_mossambicus_LEPR     R-PREGLP-V WPLLM----- -----PSENI SQVIIVDKVL T----TALIQ
Tachysurus_fulvidraco_LEPR       S-HHEGLT-A CPLLP----- -----TSENV CEVEIVEKLF VLEDDQE---
Epinephelus_coioides_LEPR        Q-PAESLS-A WPLLL----- -----PAENI SKVVIVDEVD LSALTTALIQ
Anguilla_anguilla_LEPRA          R-HPERLI-S CPLLL----- -----ESETI SEAVIVEKTS PAVQNN----
Anguilla_anguilla_LEPRB          R-HPERLT-S CPLLL----- -----EMETI SEAVIVEKAH PKAASEK---
Lepisosteus_oculatus             K-HPKRVT-S CPLLL----- -----ESETI SEAVILEKMK LGSQEQT---
Homo_sapiens_GCSFR               QLPGLGTP-- -PI---TKLT VLEEDEKKPV --------PW E---------
Danio_rerio_GCSFR                AFKEDKDP-- -VLVYLSHLS LLDMTEKEPF KSGYVKENQW PDDLN-----

                                 1051
Homo_sapiens_LEPR                VVSL-L---- ---S--TTDL EKGSVCISDQ F------NSV NFSE------
Macaca_mulatta_LEPR              VVSL-L---- ---S--TTDL EKGSVCISDQ F------NSV NFSE------
Bos_taurus_LEPR                  TDALLL---- ---T--TPDL EKGSICISDQ C------SSA QFSE------
Sus_scrofa_LEPR                  TVSLLL---- ---T--TPDL EKSSICISDQ R------SSA HFSE------
Rattus_novegicus_LEPR            MVSLLL---- ---T--TPDS TRGSICISDQ C------NSA NFSG------
Gallus_gallus_LEPR               LDSTFT---- ---K--PEDS EHDSACPSSH F------SGR SSLE------
Ornithorhynchus_anatinus_LEPR    HASLLL---- ---LAGNGKA QPGSACSSGP R------AGS SFSE------
Xenopus_tropicalis_LEPR          VTSLFT---- ---V--SEEP DHDSACESSN F------SSG CAFE------
Anolis_carolinensis_LEPR         IDSLFA---- ---T--IQDL EHDSACSSGH F------NSA SLSE------
Chrysemys_picta_bellii_LEPR      VDSMFT---- ---T--IQDS EHDSACSSSH F------SNS LSE-------
Latimeria_chalumnae_LEPR         FTSICR---- ---K--NHEI DADSACFSSF F------NSD SIDS------
Danio_rerio_LEPR                 --------KD NEEL---LSG DKTTTDSGL- --------QG DSSE------
Carassius_carassius_LEPR         --------KD NVVL-TYNSG DKTTTSSAL- --------LA DSSE------
Takifugu_rubripes_LEPR           -------PDP SVASSVRLHG EPDSPVGQAW PEESHLLPGG DRSSPPNLDY
Orizias_latipes_LEPR             TQLMSLNDDS VTSSSACLAP PFERSCLDA- ---------S APSSQ-SLD-
Salmo_salar_LEPR                 --------DK DLIPASSPAL CVDS-EVPG- --------LP EEEE------
Oreochromis_mossambicus_LEPR     NPL----PDH ADALAGSHSP GFDLNVDQF- -MENETLPVG GPSSAVDLDT
Tachysurus_fulvidraco_LEPR       --------EK ALLH-RSVDT EAKSNNLSS- --------IE GSLD------
Epinephelus_coioides_LEPR        VPLVSLTPDP DTALSISLPP GYDSEADQAQ ATESEVLLSG APSLAHDTDA
Anguilla_anguilla_LEPRA          --------ER DQVLGKAGQG EMAS-SLPP- --------CG NSEE------
Anguilla_anguilla_LEPRB          --------DR AAWAEKALH- ---------- ---------- ----------
Lepisosteus_oculatus             --------TS DASLGKKQEL DEDS-THSA- --------CQ YNEE------
Homo_sapiens_GCSFR               ---------- ---------- ---------- --------SH NSSETCGLPT
Danio_rerio_GCSFR                ---------- ---------- ---------- --------IH ADTQSCDLEL

                                 1101
Homo_sapiens_LEPR                ---------- ---------- --AEGTEVTY EAESQRQPFV KYATLISNSK
Macaca_mulatta_LEPR              ---------- ---------- --AEGTEVTC EDESQRQPFV KYATLISNSK
Bos_taurus_LEPR                  ---------- ---------- --AESTDITC EDESRRQPSV KYATLLSNSK
Sus_scrofa_LEPR                  ---------- ---------- --AESMEITR EDENRRQPSI KYATLLSSPK
Rattus_novegicus_LEPR            ---------- ---------- --AQSTQGTC EDECQSQPSV KYATLVSNVK
Gallus_gallus_LEPR               ---------- ---------- --CSP-SDPT SGETASQSNI KYATVITNSR
Ornithorhynchus_anatinus_LEPR    ---------- ---------- --GRGPG-SP WRAARRPSNV KYATLVNNSK
Xenopus_tropicalis_LEPR          ---------- ---------- --TDHQ--EM VYSSICQSSI EYATIMNNTQ
Anolis_carolinensis_LEPR         ---------- ---------- --SVC-DVET SRGMTGQSNV KYATIITNSM
Chrysemys_picta_bellii_LEPR      ---------- ---------- --SSH-DDKV SEGITRQSNI KYATIISNLK
Latimeria_chalumnae_LEPR         ---------- ---------- --ARN--EKA YPESTGQSNI KYATILNISE
Danio_rerio_LEPR                 ---------- ---------A --LEASTAAP TPETSGQSSV TYSTILLSDQ
Carassius_carassius_LEPR         ---------- ---------P LSLETSTAAP TPETSGQSSV TYSTILLSDQ
Takifugu_rubripes_LEPR           PTGSAPDDG- ---------- -----SCPAG VTDSSAQSSV IYTAVLLCGP
Orizias_latipes_LEPR             --------E- ---------- --ANQADPIV PVDSSTSSSV RYAKLLLPCL
Salmo_salar_LEPR                 ---------- ---------- TL-QLPDLPR SLESSAQPSV TYATVLLSDD
Oreochromis_mossambicus_LEPR     LTSSSSRADL QPADPSVNQH PGST------ --ENSGQSSV TYTAVLVSNP
Tachysurus_fulvidraco_LEPR       ---------- ---------P LSLDTSTVSA SPDTSGQSSV RYSTILVFDQ
Epinephelus_coioides_LEPR        LTSSSPTTDQ LQLIHPLAEQ PGSTDSSAQS SAQNSAQSSV TYATVLLPDM
Anguilla_anguilla_LEPRA          ---------- ---------P PVQETSLSPG SPDDSAQSRI SYAMVLFPGT
Anguilla_anguilla_LEPRB          ---------- ---------- ---AAVTPFS SAGSSAQSSV AYATVLPAAE
Lepisosteus_oculatus             ---------- ---------S LVQERSMHST TPERSAQSII QYATVLLPDV
Homo_sapiens_GCSFR               LVQ------- ---------T YVLQ-GDPRA VSTQPQSQSG TSDQVLYGQL
Danio_rerio_GCSFR                E--------- ---------- ---------- ------RESV PYATVLFSTP
```

```
                              1151
Homo_sapiens_LEPR             -----PSE-T GEE-QGLINS SVTKCFSSKN S---PLKDSF SNS-------
Macaca_mulatta_LEPR           -----PSE-T DEE-QGLINS SVTKCFSSKN S---PLKDSF SNS-------
Bos_taurus_LEPR               -----SGE-T EEE-QGLINS SVSKCFLSNN S---PPKDSS SKR-------
Sus_scrofa_LEPR               -----SGE-T EQE-QELVSS LVSRCFSSSN S---LPKESF SNS-------
Rattus_novegicus_LEPR         -----TVE-T DEE-QGAIHS SVSQCIARKH S---PLRQSF SSN-------
Gallus_gallus_LEPR            -----SGG-L YEQ-NKNPRC HFDGCFLAED S---LAAGAC SGS-------
Ornithorhynchus_anatinus_LEPR -----SGG-L DEQ-DRGASG SLGRGFSSKS S---LLRGAQ DGE-------
Xenopus_tropicalis_LEPR       -----QCR-K YSSERKTSLS SFDGCLLGNS S---MVIGN- ----------
Anolis_carolinensis_LEPR      -----SGG-L YEP-PKDLSS SLDRGFIGHH S---LASASF SSS-------
Chrysemys_picta_bellii_LEPR   -----SSG-L YEE-QKNLSG SFNGCFLGED S---LVTDPF -SR-------
Latimeria_chalumnae_LEPR      -----SSE-L CEQ-QKGLSS THAFGNIP-- ----SSDSDI SCN-------
Danio_rerio_LEPR              -----PSQ-L QKQQESLSSS SDEGNFSANN S---DISGSF PGG-------
Carassius_carassius_LEPR      -----PSL-L RKQQESLSSS SDEGNFSANN S---DISGSF PGG-------
Takifugu_rubripes_LEPR        K-QQQQHHHL HDKDCSCSSS SDEGNFSANN S---DISASF NGG-------
Orizias_latipes_LEPR          KQEKQP--GN PDKDGSGSNS SDEGNFSANN S---EISESS PTG-------
Salmo_salar_LEPR              -----PHH-L YKQEGSLSSS SDEGNFSGNN S---DISGSF PGG-------
Oreochromis_mossambicus_LEPR  SQDQQPPIHR PYKDGSGNSS SDEGNFSANN S---DISGSF PGG-------
Tachysurus_fulvidraco_LEPR    -----PVL-Q RKQQESLSSS SDEGNFSANN S---DISGSF PGG-------
Epinephelus_coioides_LEPR     -KQEQPPIHL HYKDGSGSSS SDEGNFSANN S---DISGSF PGG-------
Anguilla_anguilla_LEPRA       -----PGL-L YKQQESLSSS SDEGNFSANN S---DISGSF PGG-------
Anguilla_anguilla_LEPRB       -----GGRAR RRPQESLSSC SDEGNFSADT S---DMSGSY PGA-------
Lepisosteus_oculatus          -----PIH-L YKQQKSISSS SDEGNFSANN S---DISGSF PNN-------
Homo_sapiens_GCSFR            LG-------- ------SPTS PGPGHYLRCD STQPLLAGLT PSP--KSYEN
Danio_rerio_GCSFR             Y--------- ------QINS SNPPAYVRSE STQPLLAGDE PGSPPPPYEN

                              1201
Homo_sapiens_LEPR             SWEIEAQAFF ILSDQHPNII SPHL------ --TFSEGLDE LLKLEGNFPE
Macaca_mulatta_LEPR           SWEIEAQAFF ILSDQRPNII LPHL------ --TFSEGLDE LLRLEGNFPE
Bos_taurus_LEPR               SWEIETQAFF ILSDQHPNII SPHL------ --PFSEGLDE LLKLEGNFPE
Sus_scrofa_LEPR               SWEIETQAFF ILSDQHPNMT SPHL------ --SFSEGLDE LMKFEGNFPK
Rattus_novegicus_LEPR         SWEIEAQAFF LLSDHPPNVI SPQL------ --SFS-GLDE LLELEGNFPE
Gallus_gallus_LEPR            SWELGNEAFL LLPDQPGSQP CKT--LS--- -LISSEGFSE PSDQDDAFTD
Ornithorhynchus_anatinus_LEPR SEA------- -----RPP-- -----YP--- -LPSSSGLPE AVAQGVRRPR
Xenopus_tropicalis_LEPR       -HDVDKQTLV FLAGLHTKQP DKMSCNS--- -TVSSEGFSE PLDHEDSFLE
Anolis_carolinensis_LEPR      SWAMGNQGFV ILPECHQTLP RKSLSLS--- --VSSEGFSE LSEQDKAFTG
Chrysemys_picta_bellii_LEPR   SWEVGNQAFL ILPDQHPSQA SKTISLS--- -VVSSEGFSE PSDHDDTFSD
Latimeria_chalumnae_LEPR      QWESEKQITL QLEEIKPIGI NKITFCS--- -SNSSEGFSE PSNEEENDLE
Danio_rerio_LEPR              LWDLEN---- ---------S NPRHSSS--- -YNSVEEFSE TSEPDYEASE
Carassius_carassius_LEPR      LWDLENHVCS -------DSA NPRHSSS--- -YNSVEEFSD TSDQDYEASE
Takifugu_rubripes_LEPR        LWELD----- ---------- VPRRSCC--- -YNSTEELSE KPEQGD--RD
Orizias_latipes_LEPR          LWELDSCHSA -------EMD DQRRFCS--- -YASEGELSE ISEHEA-VME
Salmo_salar_LEPR              LWELEISHSG TGE----SDL DPRHSCS--- -YNSVEEFSE TSEQEDEALG
Oreochromis_mossambicus_LEPR  LWELESCRGL -------EMD DPRRSCS--- -YNSVEELSQ NSDQEE-EHE
Tachysurus_fulvidraco_LEPR    LCELERQSSS -------DAI NPRNSCS--- -YNSVEEFSE TSEQEDEASE
Epinephelus_coioides_LEPR     LWELDSCRGG -------EID DPRRSCS--- -YNSVEELSE TSEQEDEEEE
Anguilla_anguilla_LEPRA       LWELENPPSN --------DS DPRHSCC--- -YNSAEEFSD TSDQEDEVLD
Anguilla_anguilla_LEPRB       LWEPEGMPSN ---------- -PRHPCH--- SCASTEEFSE NSDQEDQSLD
Lepisosteus_oculatus          LWDVENQSCK -NS----AQE NPRNICS--- -FNSTEELSE TSDQDDYVLD
Homo_sapiens_GCSFR            LWFQAS---- ---------- -PL---GTLV TPAPSQEDDC VFGPLLNFPL
Danio_rerio_GCSFR             VPRGGAV--- ---------- SALNRFSAFS QSTQSEESDE L---WEEFPM

                              1251
Homo_sapiens_LEPR             ENND------ -KKSIYYLGV TSIKKRESGV LLTDK----- ----------
Macaca_mulatta_LEPR           ENND------ -EKSIYYLGV TSIKKRESGV LLTDK----- ----------
Bos_taurus_LEPR               ENNN------ -ERPVYYLGV TSIKKRESDV FLTNE----- ----------
Sus_scrofa_LEPR               EHND------ -ERSVYYLGV TSIKKRESDV FLTDE----- ----------
Rattus_novegicus_LEPR         ENHG------ -EKSVYYLGV SSGNKRENDM LLTDE----- ----------
Gallus_gallus_LEPR            GGSP------ -ERGLHYLGI TSLGKRENDI FLTES----- ----------
Ornithorhynchus_anatinus_LEPR DGGP------ -GQPLYYPSL PLGRSGQNEA LVAES----- ----------
Xenopus_tropicalis_LEPR       ADGL------ -ERNLYYLEF GSIQQCGQQD CYSEK----- ----------
Anolis_carolinensis_LEPR      EDSL------ -EGGMFYLGM SPFERQETDL FLTES----- ----------
Chrysemys_picta_bellii_LEPR   GDSP------ -ERSLYYLGL TSIKKNENDI FLTEN----- ----------
Latimeria_chalumnae_LEPR      EYSP------ -ERNLCYLGL TSNEEEEKES LMKAD----- ----------
Danio_rerio_LEPR              NTGL------ -AKDLYYLEM TGEEKEEEEE EEEEEEEEDE PE----EGQS
Carassius_carassius_LEPR      NTGV------ -AKDLYYLEV NEEEDEDEEE -------IKE TQ----GEQE
Takifugu_rubripes_LEPR        VG-------- EEKDLYYIGA DYGDEDEESE EEL------- -NAKLIQTVP
Orizias_latipes_LEPR          QR-------- REQTLCYLQI GYPDEDEESA EEVQREEEEK RKEQPAKDAS
Salmo_salar_LEPR              GERDGGIEVI EEKDLYYLGM GYQEESEGEE EEEEKEEEDT GAMLLKEVMV
Oreochromis_mossambicus_LEPR  VR-------- REKDLYYLGM DYPAEDEESE KEDGQSEDEE AKVELLKSTP
Tachysurus_fulvidraco_LEPR    SIQV------ -SKELYYIGM NEEEEEDELE EKFKEDDATK E---------
Epinephelus_coioides_LEPR     AR-------- EEKDLYYLGM DYPAEDEESE EDKEQIE--- -DIELLKNVV
Anguilla_anguilla_LEPRA       GTGA------ -GKELYYLGM TSQSEEEEDE KQGEDEEEE- -EEVGARYPG
Anguilla_anguilla_LEPRB       GTGS------ -RQDLYYLGT ISQSEEGEEG KP-------- -------FHS
Lepisosteus_oculatus          EMCT------ -GNDLYYVGV ASNDEENGEE EKES----F- -LMENSHVPS
Homo_sapiens_GCSFR            LQGI------ RVHGMEALGS F--------- ---------- ----------
Danio_rerio_GCSFR             LRSL------ EVNHI----- ---------- ---------- ----------
```

```
                              1301
Homo_sapiens_LEPR               -SRVSCPFPA PCLFTDIRVL QDSCSHFVEN NINLG----- TSSKKTFASY
Macaca_mulatta_LEPR             -SRVLCPFPA PCLFTDIRVL QDSCSHFVEN NFNLG----- TSSKKTFASY
Bos_taurus_LEPR                 -SRVLSPFPA HCLFTDIRIL QDSCSHLVEN NFNLG----- TSGQKTFVSY
Sus_scrofa_LEPR                 -SRVRCPFPA HCLFADIKIL QESCSHLVEN NFNLG----- TSGQKTFVSY
Rattus_novegicus_LEPR           -AGVLCPFPA HCLFSDIRIL QESCSHFVEN NLNLG----- TSGK-NFVPY
Gallus_gallus_LEPR              -SRLMCHFHT ADLLRGVGFL QNTPPNL--N AFIQS-S--- ---IKAIVPY
Ornithorhynchus_anatinus_LEPR   -PG-LTPGPS AADPGGGGAP RDAGSEF--N PFVEQDPQMG APGKRTFVSY
Xenopus_tropicalis_LEPR         -PLGTFPFQE NISYKEIDFK KDKASEFIDN YDIK------ NSFKKAFLCY
Anolis_carolinensis_LEPR        -SNMACQFHT SALIGSIRFP QNIASNLNLN PFIS----RH ETSVQTFISY
Chrysemys_picta_bellii_LEPR     -SRVMCHLHT NGLFKDMGFL QDISSDL--N PFIKN-SLKY ENSVKTFVPY
Latimeria_chalumnae_LEPR        -SNISCQFQT NGQFNTTEYP EDNTLSNFDS IFKVS-LNIK LSPKKNMRSY
Danio_rerio_LEPR                KNKRVMGVNP RPLLESQ--- ---NS----- -----TASNS NNMSHSIPLY
Carassius_carassius_LEPR        KNEIVVRVDA RPLLESK--- ---DS----- -----TSVDS NNISHSIPLY
Takifugu_rubripes_LEPR          LNSEGCSAES RRLLE----- ---------- --------LT ESKCDFSPLY
Orizias_latipes_LEPR            LNGK------ ---------- ---------- ------DFFV PLICDLSSQY
Salmo_salar_LEPR                LGREGSSVES IPLLGSQ--- ---DSMFS-- ---EYSDEGL VVGMRSVPLY
Oreochromis_mossambicus_LEPR    LNRGHCSLEL HPLLGQD--- ---NPS---- ---EPGILPS PSTCGFAMPY
Tachysurus_fulvidraco_LEPR      ---------- ---------- ---------- -------QIS DSGDSNVPLY
Epinephelus_coioides_LEPR       LSREDCSVEL HPLLSPE--- ---DSS---- ---E---LLL ASTRGFSSLY
Anguilla_anguilla_LEPRA         EDAQELPLES SPLLGRW--- ---------- ---DPRLDRT DAAAKGPPLY
Anguilla_anguilla_LEPRB         ESTLGRCLES SPLLGQQ--- ---------- ---EPWSHGE ERSGKGVPLY
Lepisosteus_oculatus            QDRDEIMQES NPLLGCH--- ---SFL---- ---NVKVNKK DVPAKNIPLY
Homo_sapiens_GCSFR              ---------- ---------- ---------- ---------- ----------
Danio_rerio_GCSFR               ---------- ---------- ---------- ---------- ----------

                              1351
Homo_sapiens_LEPR               MPQFQTCSTQ THKIMENK-- -MCDLTV
Macaca_mulatta_LEPR             MPQFQTCSTQ THKIMENK-- -MCDLTV
Bos_taurus_LEPR                 MPQFQTCSTQ TQKIMENK-- -MCDLTV
Sus_scrofa_LEPR                 MPQFQTCSTQ TQKIMENK-- -MYDLTV
Rattus_novegicus_LEPR           MPQFQSCSTH SHKIIENK-- -MCDLTV
Gallus_gallus_LEPR              VPQFQMTAAK VQETTENS-- -C-----
Ornithorhynchus_anatinus_LEPR   MPQFQRSTVR AQEAAESK-- -TCHLSA
Xenopus_tropicalis_LEPR         MPQFQTHSIK LPGEMESE-- -TLN---
Anolis_carolinensis_LEPR        MPQFQPLAIK LHEKAGGK-- -A-----
Chrysemys_picta_bellii_LEPR     MPQFQATTIK VQETTETK-- -T-----
Latimeria_chalumnae_LEPR        MPQFQIKAAK I--------- -------
Danio_rerio_LEPR                LPQFRSECIN PT-------- -------
Carassius_carassius_LEPR        LPQFRTECIN PP-------- -------
Takifugu_rubripes_LEPR          LPQFRTAPSC TRQLSAKPQE GRCHP--
Orizias_latipes_LEPR            MPQYRTAA-Y RSQLV----- -------
Salmo_salar_LEPR                LPQFRTVPSS PLKAQDSAHQ L------
Oreochromis_mossambicus_LEPR    LPQFRTAT-C TAQHTQREP- ---QL--
Tachysurus_fulvidraco_LEPR      LPQFQTAAIK LLREPAGNST IQL----
Epinephelus_coioides_LEPR       LPQFRTAP-Y TRQLTAQPHD GKPQQ--
Anguilla_anguilla_LEPRA         VPQFRTASRK SQQAKAKERS GVESLQV
Anguilla_anguilla_LEPRB         MPQFQTVVTE SQSTKGL--- -------
Lepisosteus_oculatus            MPQFQTSSNK ILKAKAQ--- -------
Homo_sapiens_GCSFR              ---------- ---------- -------
Danio_rerio_GCSFR               ---------- ---------- -------
```
