# Supplementary material for: Duplicated Leptin Receptors in Two Species of Eel Bring New Insights into the Evolution of the Leptin System in Vertebrates
Source: PLoS One. 2015 May 6;10(5):e0126008. doi: 10.1371/journal.pone.0126008 (PMC4422726; doi:10.1371/journal.pone.0126008)
Supplement: S4 Table — (DOCX) [file pone.0126008.s021.docx]

| Name | PCR Type | 5’ - 3’ sequence | amplicon size (bp) | TM °C | PCR Type |
| --- | --- | --- | --- | --- | --- |
| Leptin1 F | Classic | GTTCCAGGACACACTCTCACC | 555 | 61.8 | Classic |
| Leptin1 R | Classic | CCTCAGAGGCGATCAACACTT |  | 60.2 | Classic |
| Leptin1 qF | qPCR | TCACTGCACTTCCCTCCTCT | 154 | 59.4 | qPCR |
| Leptin1 qR | qPCR | CACCATGTTAGGAGAAATCTGGA |  | 58.9 | qPCR |
| Leptin2 F | Classic | TGCAGGAGTTCAGACCCAGA | 554 | 59.4 | Classic |
| Leptin2 R | Classic | GCGAGGGTCAGCAGCTCT |  | 60.4 | Classic |
| Leptin2 qF | qPCR | GATGGCGCAGACCACCAT | 100 | 58.2 | qPCR |
| Leptin2 qR | qPCR | GATGTCCGGGATCAGCTC |  | 58.2 | qPCR |
| LEPRA F1 | Classic | CATCCTGTGCTGGGTTGA | 2031 | 56 | Classic |
| LEPRA R1 | Classic | ATGGGCTCTCCTTCGGTATT |  | 57.3 | Classic |
| LEPRA F2 | Classic | TCTGCTCCGTCCTCATTTGT | 654 | 57.3 | Classic |
| LEPRA R2 | Classic | GATTCTGGACTGGGCTGAGT |  | 57.7 | Classic |
| LEPRA RACE F1 | RACE PCR | AAGTCTTGGGCAAAGCCGGACAGG | - | 70.7 | RACE PCR |
| LEPRA RACE F2 | RACE PCR | CCGGTCCAGGAGACATCCCTTTCC | - | 70.3 | RACE PCR |
| LEPRA RACE R1 | RACE PCR | CCAACACAGGACACTCCACAGCAGGA | - | 71.1 | RACE PCR |
| LEPRA RACE R2 | RACE PCR | TCTTGCAGCGCACCTGGACAGTGTA | - | 70.7 | RACE PCR |
| LEPRA qF | qPCR | GGGTAGAAGGAAGGGAGCAG | 164 | 61.4 | qPCR |
| LEPRA qR | qPCR | CTCATAGACAGGCAGTTCAGG |  | 59.8 | qPCR |
| LEPRB F1 | Classic | CGATGTTCGTTACTCCTCCAA | 1276 | 57.9 | Classic |
| LEPRB R1 | Classic | CCAGGGAAAGGTGTATGTGG |  | 59.4 | Classic |
| LEPRB F2 | Classic | GGTCAGGGTTCTGGAGTGAA | 1274 | 59.4 | Classic |
| LEPRB R2 | Classic | GTCCTCCTGGTCGGAGTTCT |  | 61.4 | Classic |
| LEPRB RACE F1 | RACE PCR | TGCCCTCCTGCTGATTATCGCCTTC | - | 70.6 | RACE PCR |
| LEPRB RACE F2 | RACE PCR | CGAGGGCAACTTCTCTGCCGACAC | - | 70.8 | RACE PCR |
| LEPRB RACE R1 | RACE PCR | GACTGGCTCCACTCGCTCCACAGGC | - | 71.2 | RACE PCR |
| LEPRB RACE R2 | RACE PCR | GGCTTGCCCAGGATCTTACAGCGCACC | - | 71.0 | RACE PCR |
| LEPRB qF | qPCR | ATGGGTCAGGGTTCTGGAGT | 118 | 59.4 | qPCR |
| LEPRB qR | qPCR | TTCCGTTCTCGGTCATCTTT |  | 56.3 | qPCR |
