# Supplementary material for: Duplicated Leptin Receptors in Two Species of Eel Bring New Insights into the Evolution of the Leptin System in Vertebrates
Source: PLoS One. 2015 May 6;10(5):e0126008. doi: 10.1371/journal.pone.0126008 (PMC4422726; doi:10.1371/journal.pone.0126008)
Supplement: S5 Table — (DOCX) [file pone.0126008.s022.docx]

**Table S2. Comparaison of leptin amino acid sequences**

|  | European eel leptin 1 | European eel leptin 2 |
| --- | --- | --- |
| European eel leptin 1 | - | 51.4% |
| European eel leptin 2 | 51.4% | - |
| Japanese leptin 1 | 97.7% | 52% |
| Japanese leptin 2 | 52% | 97.1% |
| Zebrafish leptina | 30.2% | 32.4% |
| Zebrafish leptinb | 28.8% | 31.4% |
| Medaka leptina | 24.7% | 25.9% |
| Medaka leptinb | 23.7% | 27.4% |
| Fugu leptin | 22.7% | 28.8% |
| Spotted gar leptin | 47.7% | 50.9% |
| Coelacanth leptin | 29.1% | 31.8% |
| Human leptin | 25.7% | 23.7% |
