# Supplementary material for: Duplicated Leptin Receptors in Two Species of Eel Bring New Insights into the Evolution of the Leptin System in Vertebrates
Source: PLoS One. 2015 May 6;10(5):e0126008. doi: 10.1371/journal.pone.0126008 (PMC4422726; doi:10.1371/journal.pone.0126008)
Supplement: S7 Table — (DOCX) [file pone.0126008.s024.docx]

**Table S6. Comparaison of LEPR amino acid sequences**

|  | European eel LEPRa | European eel LEPRb |
| --- | --- | --- |
| European eel LEPRa | - | 49% |
| European eel LEPRb | 49% | - |
| Japanese eel LEPRa | 98.9% | 49.4% |
| Japanese eel LEPRb | 49% | 98.3% |
| Zebrafish LEPR | 38.6% | 35.9% |
| Medaka LEPR | 33.7% | 31.7% |
| Salmon LEPR | 43.7% | 41.2% |
| Fugu LEPR | 37.8% | 35.6% |
| Coelacanth LEPR | 32.3% | 30.4% |
| Human LEPR | 30.3% | 29.3% |
