# Supplementary material for: Duplicated Leptin Receptors in Two Species of Eel Bring New Insights into the Evolution of the Leptin System in Vertebrates
Source: PLoS One. 2015 May 6;10(5):e0126008. doi: 10.1371/journal.pone.0126008 (PMC4422726; doi:10.1371/journal.pone.0126008)
Supplement: S8 Table — (DOCX) [file pone.0126008.s025.docx]

**Table S7. Comparaison of partial LEPR amino acid sequences**

|  | European eel LEPRa | European eel LEPRb |
| --- | --- | --- |
| European eel LEPRa | - | 51.9% |
| European eel LEPRb | 51.9% | - |
| Japanese eel LEPRa | 98.9% | 52.2% |
| Japanese eel LEPRb | 51.9% | 98.5% |
| Zebrafish LEPR | 40% | 37.8% |
| Medaka LEPR | 34.8% | 32.9% |
| Salmon LEPR | 45.9% | 43.6% |
| Fugu LEPR | 39% | 36.9% |
| Spotted gar LEPR | 53.2% | 47% |
| Coelacanth LEPR | 34.2% | 31.9% |
| Human LEPR | 32.3% | 31.6% |
